# Supplementary material for: Antigen- and scaffold-specific antibody responses to protein nanoparticle immunogens
Source: Cell Rep Med. 2022 Sep 26;3(10):100780. doi: 10.1016/j.xcrm.2022.100780 (PMC9589121; doi:10.1016/j.xcrm.2022.100780)
Supplement: Document S1. Figures S1–S8, Table S1, and Data S1 [file mmc1.pdf]

Cell Reports Medicine, Volume *3*

Supplemental information

Antigen- and scaffold-specific antibody responses to protein nanoparticle immunogens

John C. Kraft, Minh N. Pham, Laila Shehata, Mitch Brinkkemper, Seyhan Boyoglu-Barnum, Kaitlin R. Sprouse, Alexandra C. Walls, Suna Cheng, Mike Murphy, Deleah Pettie, Maggie Ahlrichs, Claire Sydeman, Max Johnson, Alyssa Blackstone, Daniel Ellis, Rashmi Ravichandran, Brooke Fiala, Samuel Wrenn, Marcos Miranda, Kwinten Sliepen, Philip J.M. Brouwer, Aleksandar Antanasijevic, David Veesler, Andrew B. Ward, Masaru Kanekiyo, Marion Pepper, Rogier W. Sanders, and Neil P. King

Cell Reports Medicine, Volume *3*

Supplemental information

Antigen- and scaffold-specific antibody responses to protein nanoparticle immunogens

John C. Kraft, Minh N. Pham, Laila Shehata, Mitch Brinkkemper, Seyhan Boyoglu-Barnum, Kaitlin R. Sprouse, Alexandra C. Walls, Suna Cheng, Mike Murphy, Deleah Pettie, Maggie Ahlrichs, Claire Sydeman, Max Johnson, Alyssa Blackstone, Daniel Ellis, Rashmi Ravichandran, Brooke Fiala, Samuel Wrenn, Marcos Miranda, Kwinten Sliepen, Philip J.M. Brouwer, Aleksandar Antanasijevic, David Veesler, Andrew B. Ward, Masaru Kanekiyo, Marion Pepper, Rogier W. Sanders, and Neil P. King

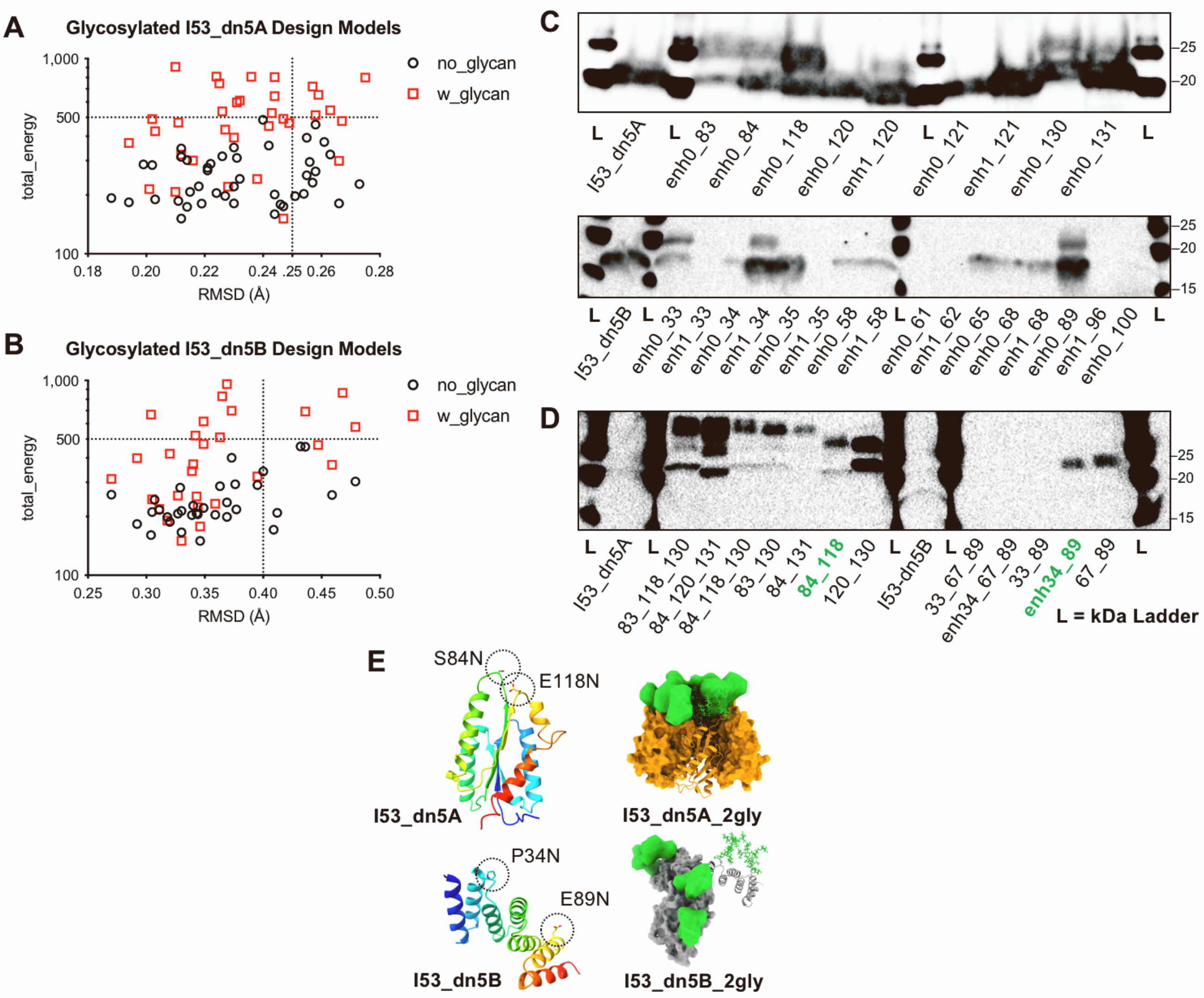

Figure S1. Design of Glycosylated I53_dn5 Nanoparticle Scaffolds, Related to Figure 1

(A and B) Rosetta total_energy vs. backbone (Cα) root mean square deviation (RMSD, Å) for design models of glycosylated I53_dn5A pentamers (A) and I53_dn5B trimers (B). Dotted lines indicate filter cut-offs for selection of designs to experimentally test for protein expression and glycosylation.

(C and D) Reducing western blots of concentrated cell supernatants for single PNGS variants (C) and combination PNGS variants (D) for glycosylated I53_dn5A pentamer and I53_dn5B trimer designs, detected using a mouse anti-myc tag primary mAb and a horse anti-mouse HRP-coupled secondary mAb. Numbers indicate the amino acid residue where an Asn was inserted. enh0, typical (non-enhanced) N-linked sequon; enh1, enhanced N-linked sequon. Glycosylated I53_dn5A and I53_dn5B variants carried forward for nanoparticle immunogen assembly and *in vivo* testing are indicated in green. L, molecular weight ladder.

(E) Schematic representations of glycosylated I53_dn5A and I53_dn5B showing the Asn insert locations on each protomer (left) and the glycosylated oligomers (right; glycans in green, I53_dn5A in orange, and I53_dn5B in gray; a single subunit of each oligomer shown as cartoon for detail).

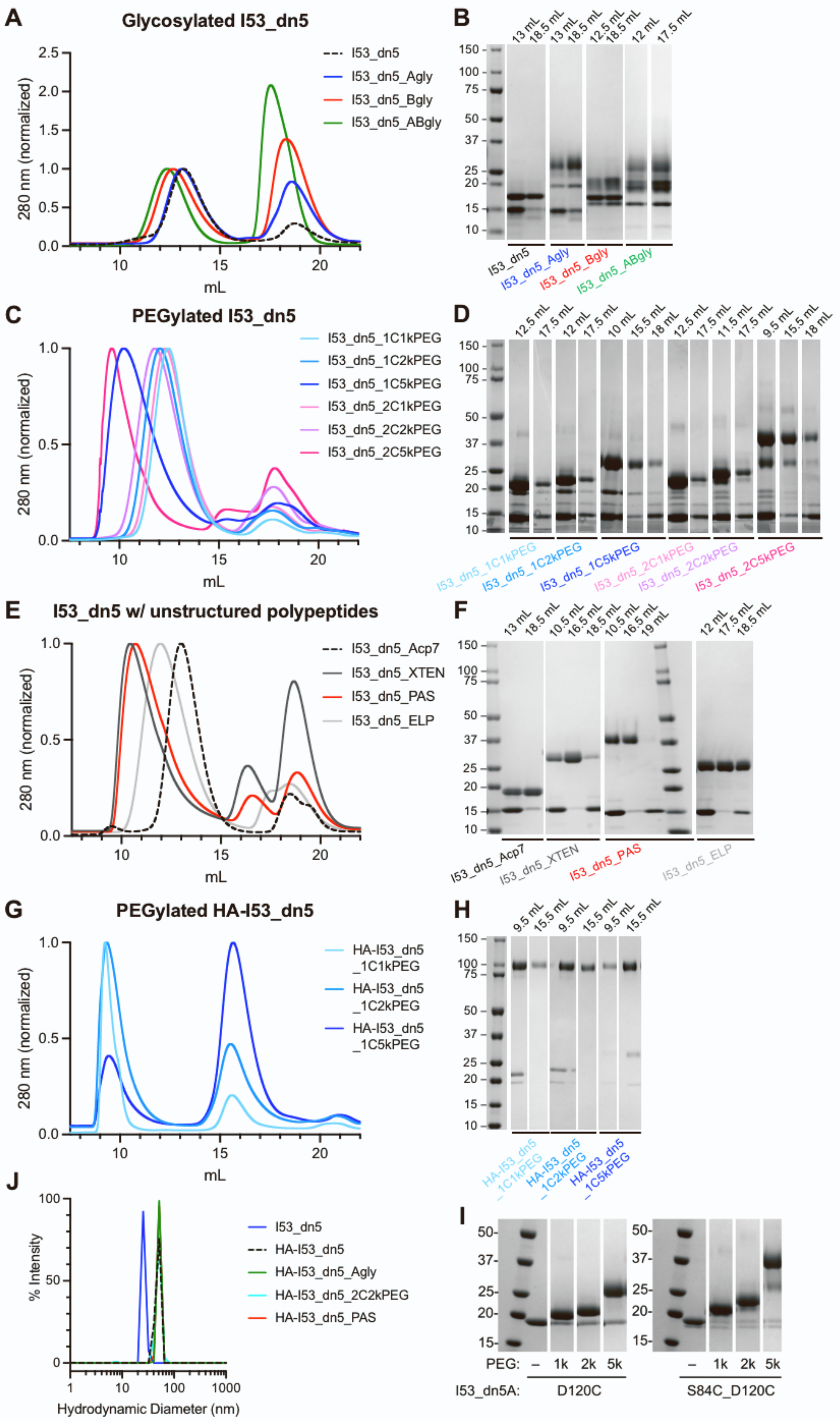
A
Glycosylated I53_dn5
I53_dn5
I53_dn5_Agly
I53_dn5_Bgly
I53_dn5_ABgly
280 nm (normalized)
mL
B
13 mL
18.5 mL
13 mL
18.5 mL
12.5 mL
18.5 mL
12 mL
17.5 mL
I53_dn5
I53_dn5_Agly
I53_dn5_Bgly
I53_dn5_ABgly
C
PEGylated I53_dn5
I53_dn5_1C1kPEG
I53_dn5_1C2kPEG
I53_dn5_1C5kPEG
I53_dn5_2C1kPEG
I53_dn5_2C2kPEG
I53_dn5_2C5kPEG
280 nm (normalized)
mL
D
12.5 mL
17.5 mL
12 mL
17.5 mL
10 mL
15.5 mL
18 mL
12.5 mL
17.5 mL
11.5 mL
17.5 mL
9.5 mL
15.5 mL
18 mL
I53_dn5_1C1kPEG
I53_dn5_1C2kPEG
I53_dn5_1C5kPEG
I53_dn5_2C1kPEG
I53_dn5_2C2kPEG
I53_dn5_2C5kPEG
E
I53_dn5 w/ unstructured polypeptides
I53_dn5_Acp7
I53_dn5_XTEN
I53_dn5_PAS
I53_dn5_ELP
280 nm (normalized)
mL
F
13 mL
18.5 mL
10.5 mL
16.5 mL
18.5 mL
10.5 mL
16.5 mL
19 mL
12 mL
17.5 mL
18.5 mL
I53_dn5_Acp7
I53_dn5_XTEN
I53_dn5_PAS
I53_dn5_ELP
G
PEGylated HA-I53_dn5
HA-I53_dn5 _1C1kPEG
HA-I53_dn5 _1C2kPEG
HA-I53_dn5 _1C5kPEG
280 nm (normalized)
mL
H
9.5 mL
15.5 mL
9.5 mL
15.5 mL
9.5 mL
15.5 mL
HA-I53_dn5 _1C1kPEG
HA-I53_dn5 _1C2kPEG
HA-I53_dn5 _1C5kPEG
J
I53_dn5
HA-I53_dn5
HA-I53_dn5_Agly
HA-I53_dn5_2C2kPEG
HA-I53_dn5_PAS
% Intensity
Hydrodynamic Diameter (nm)
I
PEG:
–
1k
2k
5k
I53_dn5A:
D120C
S84C_D120C

Figure S2. Characterization of Glycosylated, PEGylated, and PASylated I53_dn5 Nanoparticle Scaffolds, Related to Figure 1

(A, C, and E) SEC purification of the I53_dn5 scaffold masked with glycans (A), PEG (C), or unstructured polypeptides (E) after *in vitro* assembly using a Superose 6 Increase 10/300 GL column. The nanoparticles elute at 9-15 mL and residual, unassembled components elute at larger volumes. In addition to the peak shifts being consistent with the molecular weight of the masking agent, in most cases modest effects on the *in vitro* assembly efficiency were also observed.

(B, D, and F) Reducing SDS-PAGE of SEC-purified I53_dn5 scaffold masked with glycans (D), PEG (D), or unstructured polypeptides (F) and residual, unassembled components. The presence of more unassembled components in the 18.5 mL peak for I53_dn5_Bgly compared to I53_dn5_Agly indicates that the I53_dn5B_2gly component has the lower nanoparticle assembly efficiency (A and B). Similarly, 5 kDa PEG, XTEN, and PAS polypeptides all have larger amounts of unassembled components in the 15-20 mL elution volumes compared to the smaller 1 and 2 kDa PEG and ELP polypeptide, indicating that these larger masking agents impeded nanoparticle assembly efficiency the most (C-F). From the SDS-PAGE presented in panel (D), we estimate PEG conjugation efficiency was >90% in all cases.

(G) SEC purification of PEGylated HA-I53_dn5 nanoparticle immunogens after *in vitro* assembly using a Superose 6 Increase 10/300 GL column. The nanoparticle immunogen elutes at the void volume. Residual, unassembled components elute around 15-18 mL. Note the declining *in vitro* assembly efficiency as the PEG molecular weight increases, suggesting larger PEG sterically hinders nanoparticle assembly when HA is fused to the I53_dn5B trimer.

(H) Reducing SDS-PAGE of SEC-purified PEGylated HA-I53_dn5 nanoparticle immunogens and residual, unassembled components. Only excess HA-I53_dn5B trimer was detected in the residual, unassembled component peak for both HA-I53_dn5_1C1kPEG and HA-I53_dn5_1C2kPEG immunogens, confirming complete nanoparticle assembly. However, both HA-I53_dn5B trimer and I53_dn5A_1C5kPEG pentamer were present in the 15.5 mL unassembled component peak for HA-I53_dn5_1C5kPEG immunogen, indicating that 5 kDa PEG on the I53_dn5A pentamer impeded efficient nanoparticle assembly.

(I) Reducing SDS-PAGE of I53_dn5A_D120C and I53_dn5A_S84C_D120C pentamers coupled to 1, 2, or 5 kDa PEG. Note the larger molecular weight shifts when PEG is coupled to I53_dn5A_S84C_D120C compared to I53_dn5A_D120C due to the presence of two unpaired cysteines (10 vs. 5 cysteines per pentamer, respectively). We estimate PEG conjugation efficiency was >90% in all cases.

(J) Dynamic light scattering of SEC-purified nanoparticle immunogens, including unmodified I53_dn5.

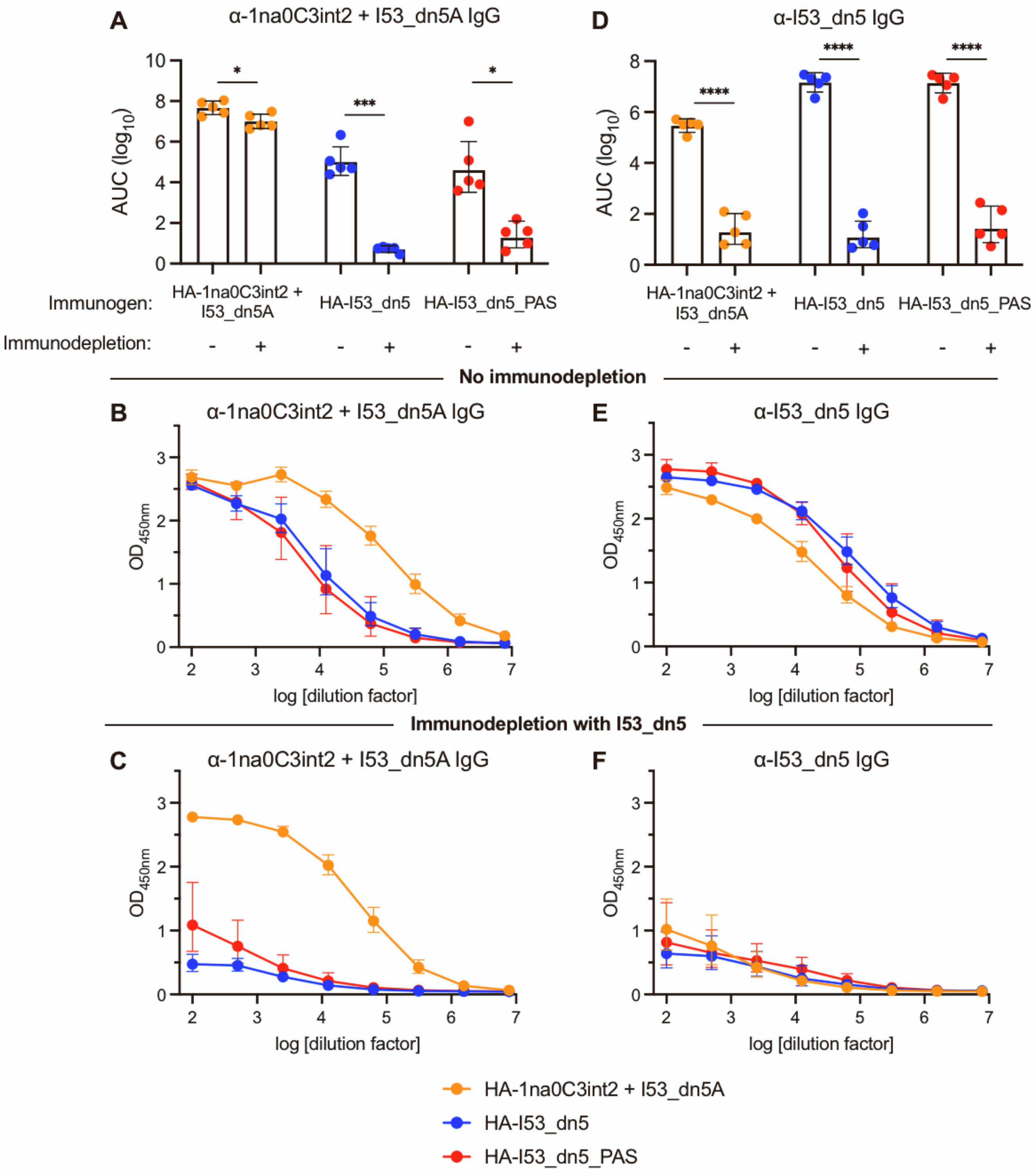
A
α-1na0C3int2 + I53_dn5A IgG
AUC (log10)
*
***
*
D
α-I53_dn5 IgG
****
****
****
Immunogen:
HA-1na0C3int2 + I53_dn5A
HA-I53_dn5
HA-I53_dn5_PAS
Immunodepletion:
- + - + - +
No immunodepletion
B
α-1na0C3int2 + I53_dn5A IgG
OD450nm
log [dilution factor]
E
α-I53_dn5 IgG
Immunodepletion with I53_dn5
C
α-1na0C3int2 + I53_dn5A IgG
F
α-I53_dn5 IgG
HA-1na0C3int2 + I53_dn5A
HA-I53_dn5
HA-I53_dn5_PAS

Figure S3. HA-I53_dn5 and HA-I53_dn5_PAS Nanoparticle Immunogens Elicit Minimal Antibodies Against Epitopes on the Interior Surface of the Nanoparticle Scaffold, Related to Figure 2

(A-F) Post-2nd boost (week 10) anti-unassembled HA-I53_dn5 nanoparticle components (HA-1na0C3int2 trimer + I53_dn5A pentamer) (A-C) and anti-I53_dn5 nanoparticle (D-F) serum IgG binding titers in BALB/c mice, measured by ELISA and plotted as the area under the curve (AUC) (A and D) for each serum dilution series (B, C, E, and F). Week 10 sera from mice immunized with either HA-1na0C3int2 + I53_dn5A unassembled components, HA-I53_dn5 nanoparticles, or HA-I53_dn5_PAS nanoparticles were analyzed for antibody titer against unassembled HA-I53_dn5 nanoparticle components (HA-1na0C3int2 trimer + I53_dn5A pentamer) (A-C) or I53_dn5 nanoparticles (D-F) before (B and E) or after (C and F) immunodepletion with I53_dn5. (A and D) Each symbol represents an individual animal and the geometric mean AUC and the geometric mean standard deviation from each group is indicated by the bar and error bar, respectively (N=5 mice/group).

P values between groups were determined by Brown-Forsythe and Welch one-way ANOVA test, with Dunnett's T3 multiple comparisons test. *$p < 0.05$; **$p < 0.01$; ***$p < 0.001$; ****$p < 0.0001$.

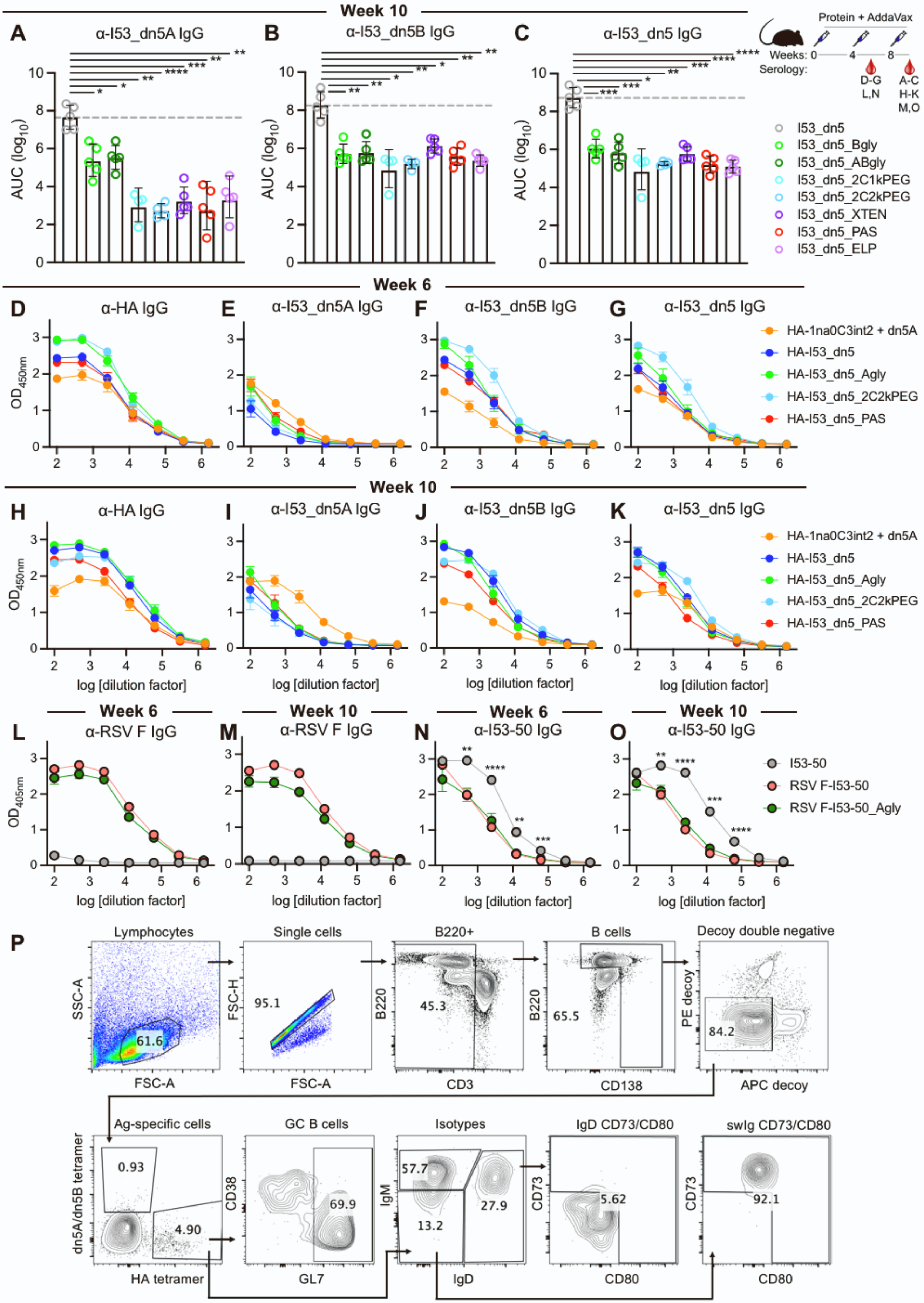

Week 10
A
α-I53_dn5A IgG
B
α-I53_dn5B IgG
C
α-I53_dn5 IgG
AUC (log10)
Protein + AddaVax
Weeks: 0 4 8
Serology:
D-G L,N
A-C H-K M,O
I53_dn5
I53_dn5_Bgly
I53_dn5_ABgly
I53_dn5_2C1kPEG
I53_dn5_2C2kPEG
I53_dn5_XTEN
I53_dn5_PAS
I53_dn5_ELP
Week 6
D
α-HA IgG
E
α-I53_dn5A IgG
F
α-I53_dn5B IgG
G
α-I53_dn5 IgG
OD450nm
HA-1na0C3int2 + dn5A
HA-I53_dn5
HA-I53_dn5_Agly
HA-I53_dn5_2C2kPEG
HA-I53_dn5_PAS
Week 10
H
α-HA IgG
I
α-I53_dn5A IgG
J
α-I53_dn5B IgG
K
α-I53_dn5 IgG
log [dilution factor]
Week 6
Week 10
L
α-RSV F IgG
M
α-RSV F IgG
N
α-I53-50 IgG
O
α-I53-50 IgG
OD405nm
I53-50
RSV F-I53-50
RSV F-I53-50_Agly
P
Lymphocytes
Single cells
B220+
B cells
Decoy double negative
SSC-A
FSC-A
FSC-H
B220
CD3
CD138
PE decoy
APC decoy
61.6
95.1
45.3
65.5
84.2
Ag-specific cells
GC B cells
Isotypes
IgD CD73/CD80
swIg CD73/CD80
dn5A/dn5B tetramer
HA tetramer
CD38
GL7
IgM
IgD
CD73
CD80
0.93
4.90
69.9
57.7
13.2
27.9
5.62
92.1

Figure S4. Masking the I53_dn5 Scaffold Reduces Scaffold-specific Antibody Responses when no Glycoprotein Antigen is Present, but Scaffold Masking does not Enhance Antigen-specific Responses when I53_dn5 and I53-50 Scaffolds Display a Glycoprotein Antigen, Related to Figure 2

(A-C) Post-2nd boost (week 10) anti-I53_dn5A pentamer (A), anti-I53_dn5B trimer (B), and anti-I53_dn5 nanoparticle (C) serum IgG binding titers in BALB/c mice, measured by ELISA and plotted as the area under the curve (AUC) for each serum dilution series. Each symbol represents an individual animal and the geometric mean AUC and the geometric mean standard deviation from each group is indicated by the bar and error bar, respectively (N=5 mice/group). The gray dashed line represents levels for the unmodified I53_dn5 nanoparticle for comparison. The inset depicts the study timeline and the blood collection time point that each data panel represents. *P* values between groups were determined by Brown-Forsythe and Welch one-way ANOVA test, with Dunnett's T3 multiple comparisons test. $*p < 0.05$; $**p < 0.01$; $***p < 0.001$; $****p < 0.0001$.

(D-K) Post-1st boost (week 6) (D-G) and post-2nd boost (week 10) (H-K) anti-H1MI15 influenza hemagglutinin (D and H), anti-I53_dn5A pentamer (E and I), anti-I53_dn5B trimer (F and J), and anti-I53_dn5 nanoparticle (G and K) serum IgG ELISA curves in BALB/c mice. Each symbol represents the geometric mean absorbance at 450 nm +/- geometric mean SD (N=5 mice/group).

(L-O) Post-1st boost (week 6) (L and N) and post-2nd boost (week 10) (M and O) anti-DS-Cav1 RSV F protein (L and M) and anti-I53-50 nanoparticle (N and O) IgG ELISA curves in BALB/c mice. Each symbol represents the geometric mean absorbance at 450 nm +/- geometric mean SD (N=5 mice/group). *P* values between the 405 nm absorption values for I53-50 and RSV F-I53-50 at the indicated serum dilutions were determined by unpaired *t* tests. $*p < 0.05$; $**p < 0.01$; $***p < 0.001$; $****p < 0.0001$.

(P) Representative gating strategy for evaluating I53_dn5A-, I53_dn5B-, and HA-specific B cells, germinal center (GC) precursors and B cells ($CD38^{+/-}GL7^{+}$), and B cell isotypes. Top row, gating strategy for measuring numbers of live, non-doublet B cells. Bottom row, representative data from a mouse immunized with HA-I53_dn5 formulated with AddaVax. $HA^{+}CD38^{+/-}GL7^{+}$ cells that did not bind decoys were counted as antigen-specific GC precursors and B cells. GC precursors and B cells were further analyzed to characterize B cell receptor isotypes.

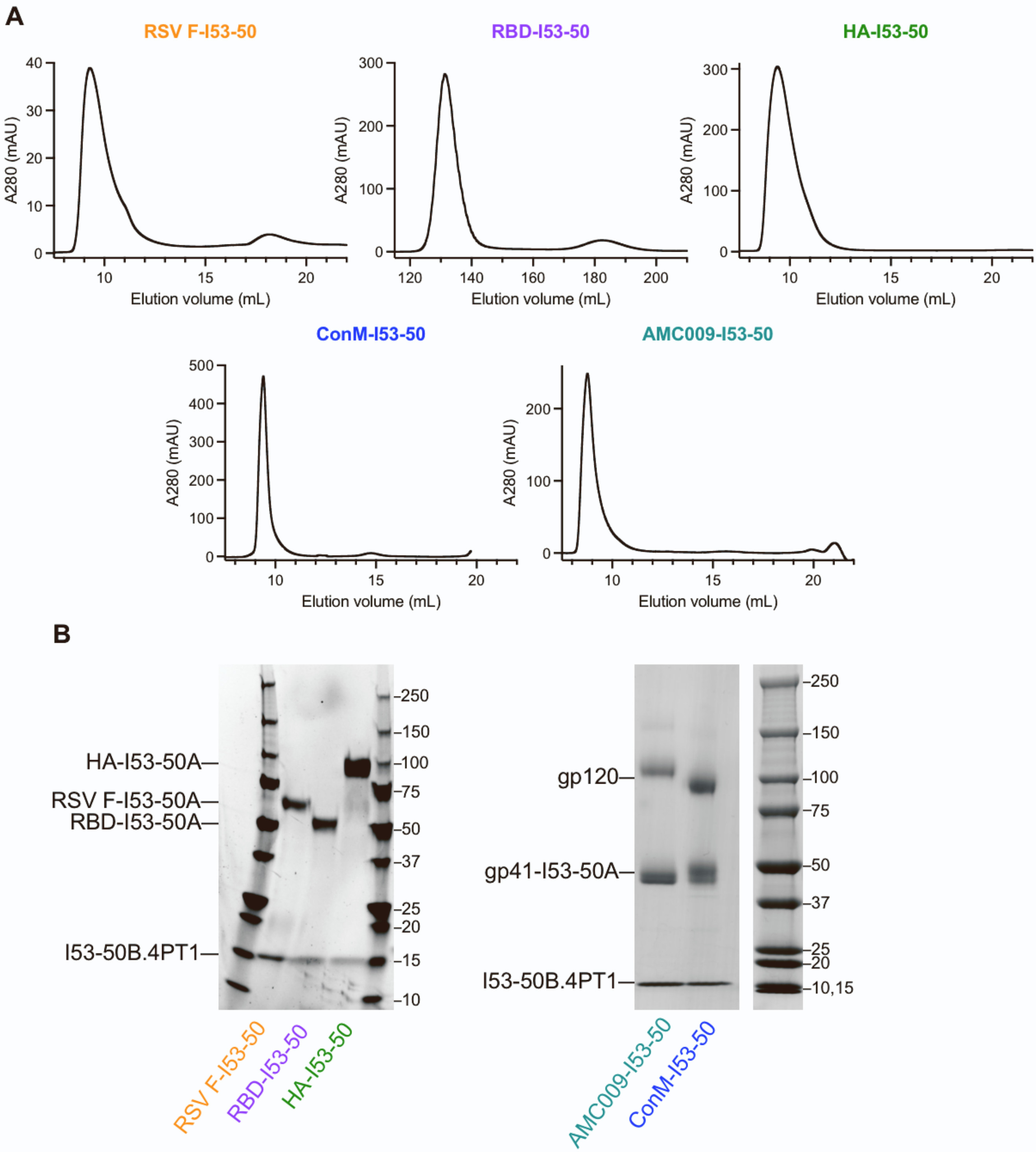

Figure S5. SEC Purification and SDS-PAGE of I53-50-based Nanoparticle Immunogens, Related to Figure 3
(A and B) SEC chromatograms from purification of the RSV F-I53-50, RBD-I53-50, HA-I53-50, ConM-I53-50, and AMC009-I53-50 nanoparticle immunogens after *in vitro* assembly using a HiLoad 26/600 Superdex 200 pg column for RBD-I53-50 and a Superose 6 Increase 10/300 GL column for the other nanoparticle immunogens (A), and SDS-PAGE of these nanoparticle immunogens after SEC purification (B).

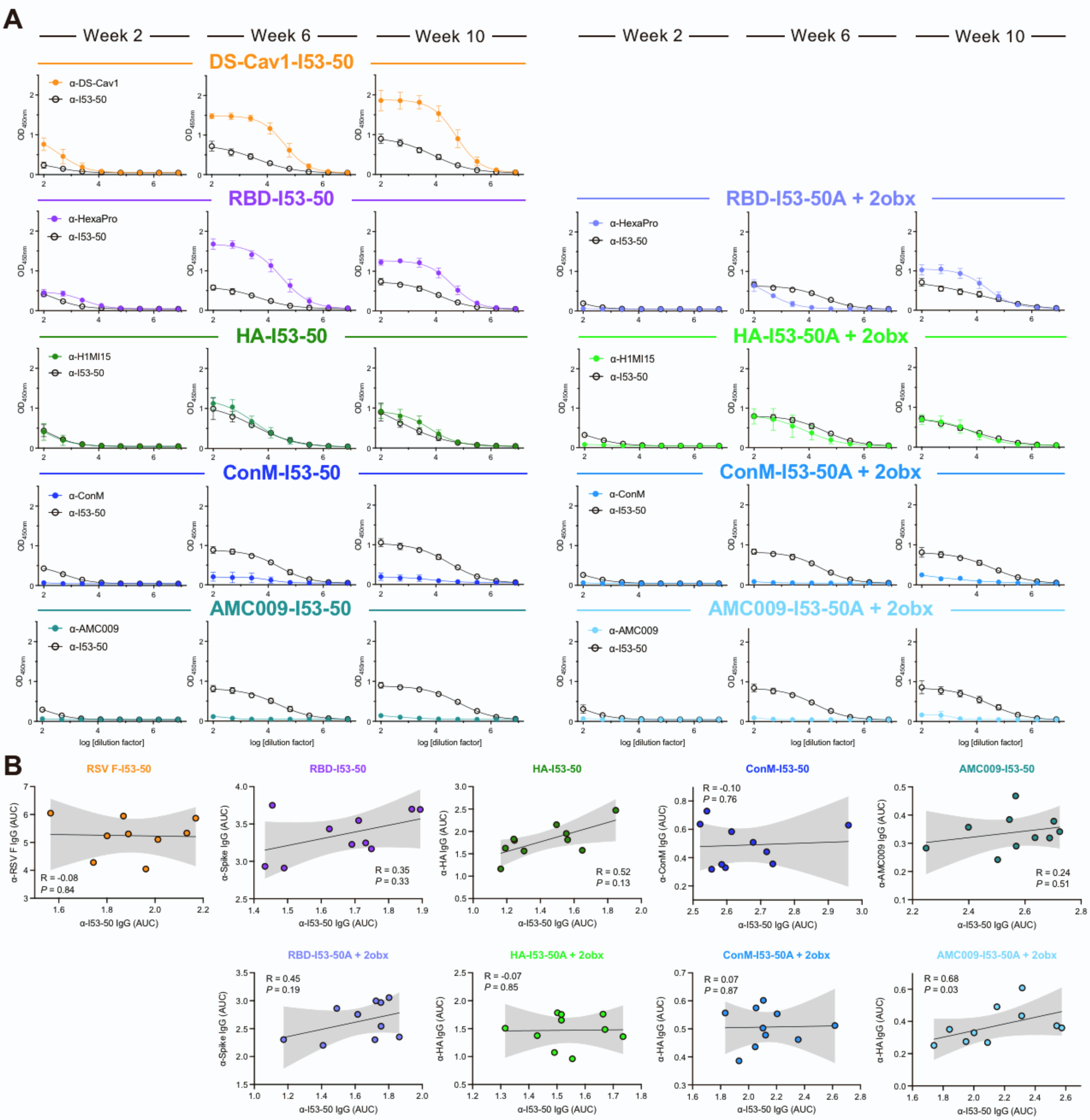

Figure S6. Individual ELISA Curves for a Series of Different I53-50-based Nanoparticle Immunogens and Non-assembling Controls, Related to Figure 3

(A) Post-prime (week 2), post-1st boost (week 6), and post-2nd boost (week 10) antigen-specific (solid colored circles) and anti-I53-50 nanoparticle (open black circles) serum IgG ELISA curves in BALB/c mice. Antigen-specific IgG titers were measured by Ni-NTA-capture ELISA. Each symbol represents the geometric mean absorbance at 450 nm +/- geometric mean SD (N=10 mice/group). Immunogen labels are listed at the top of their respective ELISA plots.

(B) Spearman's correlations between post-2nd boost (week 10) anti-antigen and anti-I53-50 scaffold serum IgG titers (AUC) for each individual immunogen. Shaded areas represent 95% confidence intervals of the plotted linear regression line. Each symbol represents a mouse (N=10 per immunogen).

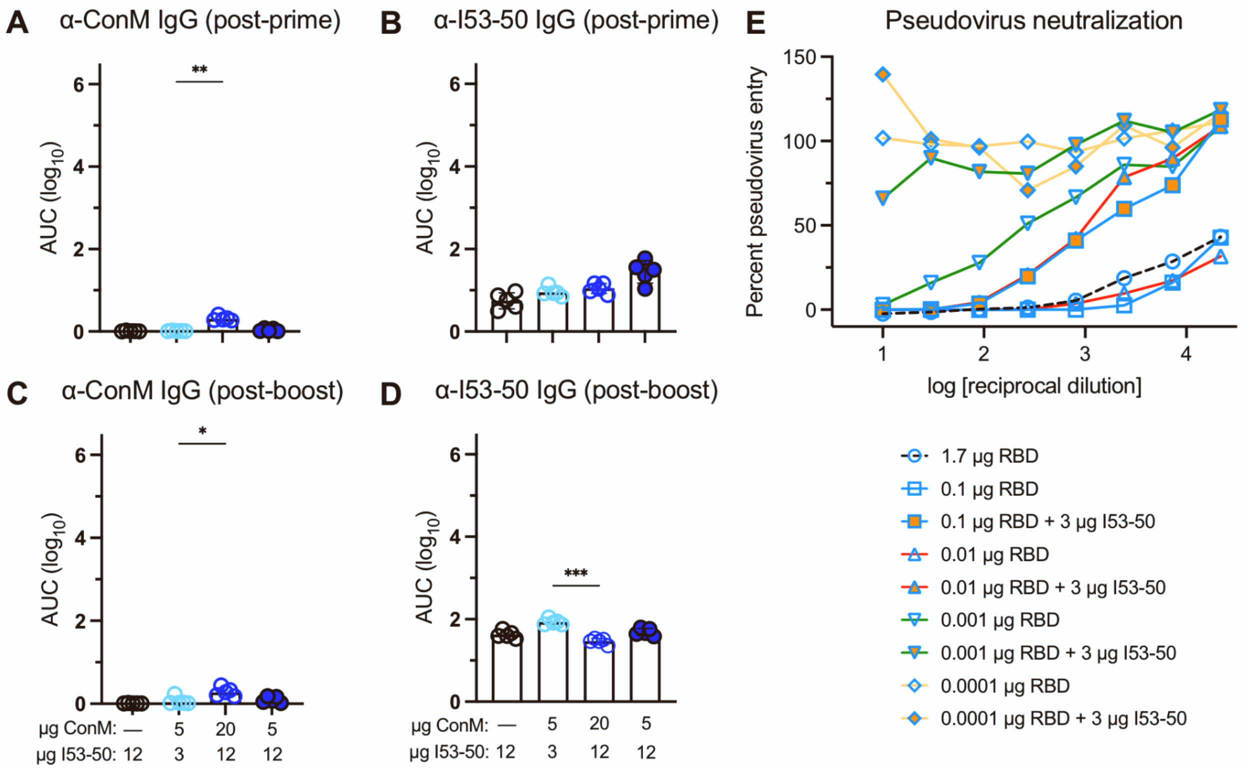

Figure S7. Anti-ConM and Anti-I53-50 Antibody Responses at Different ConM-I53-50 Doses and with Co-delivered Excess I53-50 Scaffold, Related to Figure 5

(A-D) Post-prime (week 2) (A and B) and post-boost (week 5) (C and D) anti-HIV-1 Env (ConM) (A and C) and anti-I53-50 scaffold (B and D) serum IgG binding titers in BALB/c mice immunized with the protein doses indicated at the bottom, measured by ELISA and plotted as the area under the curve (AUC) for each serum dilution series. Each symbol represents an individual animal and the geometric mean AUC from each group is indicated by the bar (N=5 mice/group). *P* values between groups were determined by Brown-Forsythe and Welch one-way ANOVA test, with Dunnett's T3 multiple comparisons test. *$p < 0.05$; **$p < 0.01$; ***$p < 0.001$; ****$p < 0.0001$.

(E) Percent pseudovirus entry curves used for calculating reciprocal IC_{50} titer (log_{10}) values in Figure 5J. Each symbol represents the arithmetic mean of N=5 mice/group.

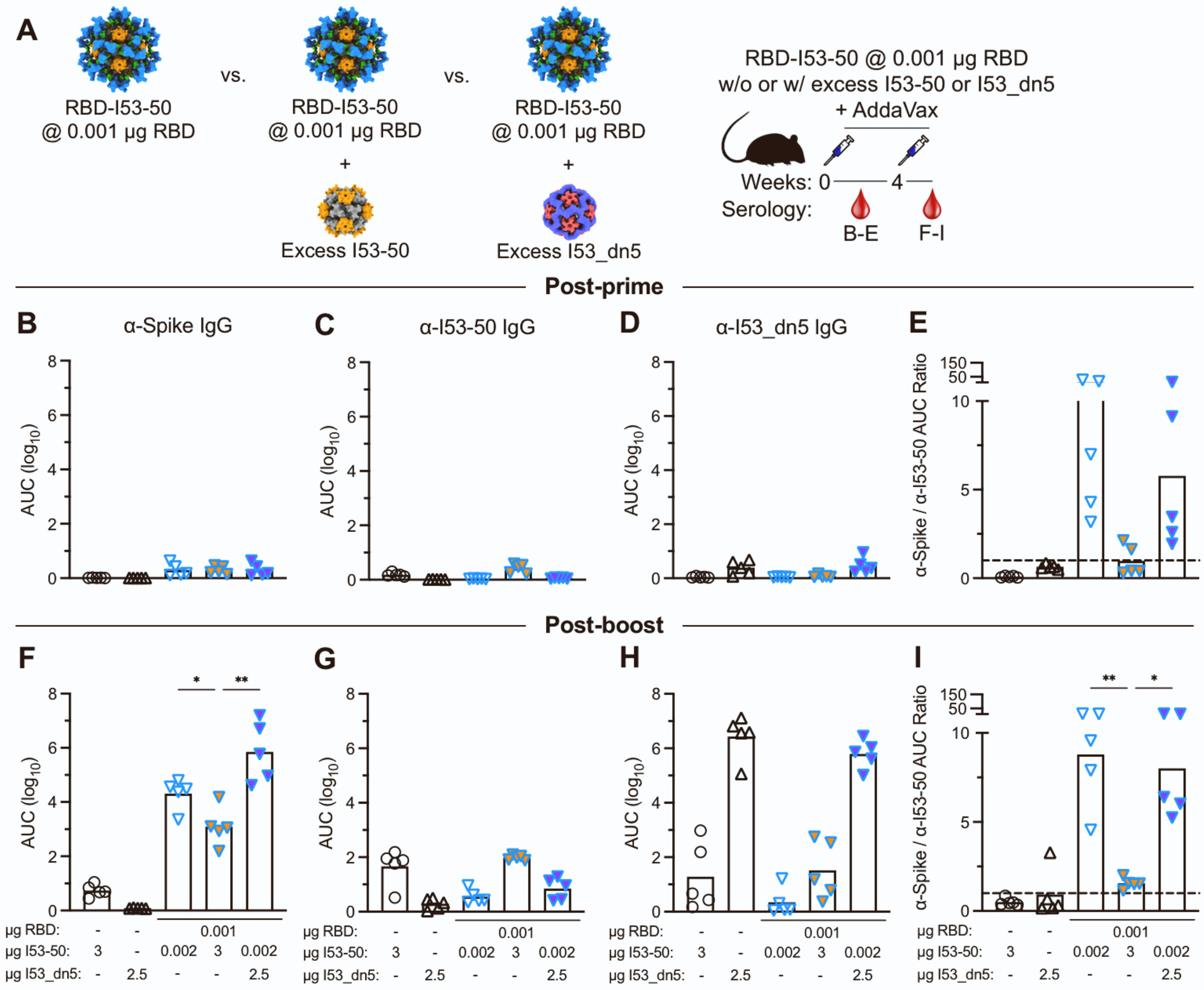

Figure S8. Heterologous Scaffold-specific Antibody Responses do not Competitively Inhibit Antigen-specific Responses when Heterologous Scaffold is Provided in Excess, Related to Figure 5

(A) (Left) Schematic representation of the homologous I53-50 and heterologous I53_dn5 scaffold competition experimental design where mice were immunized with the RBD-I53-50 nanoparticle immunogen at an RBD dose of 0.001 µg in the absence of excess scaffold, or co-delivered with excess homologous I53-50 or heterologous I53_dn5 nanoparticle scaffold. (Right) Schematic depicting the study timeline and blood collection time points that each data panel represents.

(B-I) Post-prime (week 2) (B-E) and post-boost (week 6) (F-I) anti-SARS-CoV-2 Spike (B and F), anti-I53-50 scaffold (C and G), and anti-I53_dn5 scaffold serum IgG binding titers in BALB/c mice immunized with the protein doses indicated at the bottom of F-I, measured by ELISA and plotted as the area under the curve (AUC) for each serum dilution series. Each symbol represents an individual animal and the geometric mean AUC from each group is indicated by the bar (N=5 mice/group).

(E and I) Ratio of the post-prime (week 2) (E) and post-boost (week 6) (I) Spike-specific to I53-50 scaffold-specific binding antibody titers (AUC). The black dashed line indicates a ratio of 1.

P values between groups were determined by Brown-Forsythe and Welch one-way ANOVA test, with Dunnett's T3 multiple comparisons test. *p < 0.05; **p < 0.01; ***p < 0.001; ****p < 0.0001.

Table S1. Amino acid sequences for proteins used in this study, Related to STAR Methods

Non-antigen-bearing nanoparticle components

>I53_dn5A pentamer
MGKYDGSKLRIGILHARWNAEIILALVLGALKRLQEFGVKRENIIIETVPGSFELPYGSKLFVEKQKRLGKPLDAIIPIGVLIKGSTMHFEY
ICDSTTHQLMKLNFELGIPVIFGVLTCLTDEQAEARAGLIEGKMHNHGEDWGAAAVEMATKFNLEHHHHHH

>I53_dn5A_1cys pentamer
MGKYDGSKLRIGILHARGNAEIILALVLGALKRLQEFGVKRENIIIETVPGSFELPYGSKLFVEKQKRLGKPLDAIIPIGVLIRGSTPHFDY
IADSTTHQLMKLNFELGIPVIFGVITADTCEQAEARAGLIEGKMHNHGEDWGAAAVEMATKFNGGWELQLEGSHHHHHH

>I53_dn5A_2cys pentamer
MGKYDGSKLRIGILHARGNAEIILALVLGALKRLQEFGVKRENIIIETVPGSFELPYGSKLFVEKQKRLGKPLDAIIPIGVLIRGCTPHFDY
IADSTTHQLMKLNFELGIPVIFGVITADTCEQAEARAGLIEGKMHNHGEDWGAAAVEMATKFNGGWELQLEGSHHHHHH

>I53_dn5Acp7_ELP1 pentamer
MGSHHHHHHGSDEQAEERAGTKAGNHGEDWGAAAVEMATKFNGSGGSGKYDGSKLRIGILHARGNAEIILELVLGALKRLQEFGVKRENIII
ETVPGSFELPYGSKLFVEKQKRLGKPLDAIIPIGVLIRGSTAHFDYIADSTTHQLMKLNFELGIPVIFGVLTTESGGSVPGAGVPGVGVPGV
GVPGAGVPGVGVPGAGVPGVGVPGAGVPGVGVPGVGVPGAGVPGVG

>I53_dn5Acp7_ELP2 pentamer
MGSHHHHHHGSDEQAEERAGTKAGNHGEDWGAAAVEMATKFNGSGGSGKYDGSKLRIGILHARGNAEIILELVLGALKRLQEFGVKRENIII
ETVPGSFELPYGSKLFVEKQKRLGKPLDAIIPIGVLIRGSTAHFDYIADSTTHQLMKLNFELGIPVIFGVLTTESGGSVPGAGVPGAGVPGA
GVPGAGVPGAGVPGAGVPGAGVPGAGVPGAGVPGAGVPGAGVPGAG

>I53_dn5Acp7_PAS pentamer
MGSHHHHHHGSDEQAEERAGTKAGNHGEDWGAAAVEMATKFNGSGGSGKYDGSKLRIGILHARGNAEIILELVLGALKRLQEFGVKRENIII
ETVPGSFELPYGSKLFVEKQKRLGKPLDAIIPIGVLIRGSTAHFDYIADSTTHQLMKLNFELGIPVIFGVLTTESGGSASPAAPAPASPAAP
APSAPAAASPAAPAPASPAAPAPSAPAAASPAAPAPASPAAPAPSAPAA

>I53_dn5A pentamer (I53_dn5A.2, optimized for mammalian cell secretion)
MDSKGSSQKGSRLLLLLVVSNLLLPQGVLAKYDGSKLRIGILHARGNAEIILELVLGALKRLQEFGVKRENIIIETVPGSFELPYGSKLFVE
KQKRLGKPLDAIIPIGVLIRGSTAHFDYIADSTTHQLMKLNFELGIPVIFGVLTTESDEQAEERAGTKAGNHGEDWGAAAVEMATKFNLEEQ
KLISEEDLHHHHHH

>I53_dn5A_2gly pentamer
MDSKGSSQKGSRLLLLLVVSNLLLPQGVLAKYDGSKLRIGILHARGNAEIILELVLGALKRLQEFGVKRENIIIETVPGSFELPYGSKLFVE
KQKRLGKPLDAIIPIGVLIRGNDTHFDYIADSTTHQLMKLNFELGIPVIFGVLTTNSTEQAEERAGTKAGNHGEDWGAAAVEMATKFNLEEQ
KLISEEDLHHHHHH

>I53_dn5B trimer
MEEAELAYLLGELAYKLGEYRIAIRAYRIALKRDPNNAEAWYNLGNAYYKQGRYREAIEYYQKALELDPNNAEAWYNLGNAYYERGEYEEAI
EYYRKALRLDPNNADAMQNLLNAKMREEGGWELQGSLEHHHHHH

>I53_dn5B_2gly trimer
MDSKGSSQKGSRLLLLLVVSNLLLPQGVLAEEAELAYLLGELAYKLGEYRIAIRAYRIALKYDNLTAEAWYNLGNAYYKQGRYREAIEYYQK
ALELDPNNAEAWYNLGNAYYERGEYENATEYYRKALRLDPNNADAMQNLLNAKMREELEEQKLISEEDLHHHHHH

>I53-50A trimer

MKMEELFKKHKIVAVLRANSVEEAIEKAVAVFAGGVHLIEITFTVPDADTVIKALSVLKEKGAIIGAGTVTSVEQCRKAVESGAEFIVSPHL
DEEISQFCKEKGVFYMPGVMTPTELVKAMKLGHTILKLFPGEVVGPQFVKAMKGPFPNVKFVPTGGVNLDNVCEWFKAGVLAVGVGSALVKG
TPDEVREKAKAFVEKIRGCLEEQKLISEEDLHHHHHH

>I53-50A_4gly trimer
MDSKGSSQKGSRLLLLLVVSNLLLPQGVLAEELFKKHKIVAVLRANSVEEAIEKAVAVFAGGVHLIEITFTVPNATTVIKALSVLKEKGAII
GAGTVTSVEYANETVESGAEFIVSPHLDEEISNFTKEKGVFYMPGVMTPTELVKAMKLGHTILKLFPGEVVGPQFVKAMKGPFHNATFVPTG
GVNLDNVCEWFKAGVLAVGVGSALVKGTPDEVREKAKAFVEKIRGCLEEQKLISEEDLHHHHHH

>I53-50B.4PT1 pentamer
MNQHSHKDHETVRIAVVRARWHAEIVDACVSAFEAAMRDIGGDRFAVDVFDVPGAYEIPLHARTLAETGR
YGAVLGTAFVVNGGIYRHEFVASAVINGMMNVQLNTGVPVLSAVLTPHNYDKSKAHTLLFLALFAVKGME
AARACVEILAAREKIAAGSLEHHHHHH

>2obx pentamer
MNQHSHKDYETVRIAVVRARWHADIVDQCVSAFEAEMADIGGDRFAVDVFDVPGAYEIPLHARTLAETGR
YGAVLGTAFVVNGGIYRHEFVASAVIDGMMNVQLSTGVPVLSAVLTPHNYHDSAEHHRFFFEHFTVKGKE
AARACVEILAAREKIAAGSLEHHHHHH

Antigen-bearing nanoparticle components and ELISA antigens

>H1MI15-I53_dn5B trimer (A/Michigan/45/2015 HA 1-676 Y98F no lkr dn5B.SA.WELQ-H)
MKAILVVLLYTFTTANADTLCIGYHANNSTDTVDTVLEKNVTVTHSVNLLEDKHNGKLCKLRGVAPLHLGKCNIAGWILGNPECESLSTASS
WSYIVETSNSDNGTCFPGDFINYEELREQLSSVSSFERFEIFPKTSSWPNHDSNKGVTAACPHAGAKSFYKNLIWLVKKGNSYPKLNQSYIN
DKGKEVLVLWGIHHPSTTADQQSLYQNADAYVFVGTSRYSKKFKPEIATRPKVRDQEGRMNYYWTLVEPGDKITFEATGNLVVPRYAFTMER
NAGSGIIISDTPVHDCNTTCQTPEGAINTSLPFQNIHPITIGKCPKYVKSTKLRLATGLRNVPSIQSRGLFGAIAGFIEGGWTGMVDGWYGY
HHQNEQGSGYAADLKSTQNAIDKITNKVNSVIEKMNTQFTAVGKEFNHLEKRIENLNKKVDDGFLDIWTYNAELLVLLENERTLDYHDSNVK
NLYEKVRNQLKNNAKEIGNGCFEFYHKCDNTCMESVKNGTYDYPKYSEEAKLNREKIDGVSAEEEAELAYLLGELAYKLGEYRIAIRAYRIAL
KRDPNNAEAWYNLGNAYYKQGRYREAIEYYQKALELDPNNAEAWYNLGNAYYERGEYEEAIEYYRKALRLDPNNADAMQNLLNAKMREEGGW
ELQHHHHHH

>H1MI15-I53-50A trimer (A/Michigan/45/2015 HA 1-676 Y98F)
MDSKGSSQKGSRLLLLLVVSNLLLPQGVLADTLCIGYHANNSTDTVDTVLEKNVTVTHSVNLLEDKHNGKLCKLRGVAPLHLGKCNIAGWIL
GNPECESLSTASSWSYIVETSNSDNGTCFPGDFINYEELREQLSSVSSFERFEIFPKTSSWPNHDSNKGVTAACPHAGAKSFYKNLIWLVKK
GNSYPKLNQSYINDKGKEVLVLWGIHHPSTTADQQSLYQNADAYVFVGTSRYSKKFKPEIATRPKVRDQEGRMNYYWTLVEPGDKITFEATG
NLVVPRYAFTMERNAGSGIIISDTPVHDCNTTCQTPEGAINTSLPFQNIHPITIGKCPKYVKSTKLRLATGLRNVPSIQSRGLFGAIAGFIE
GGWTGMVDGWYGYHHQNEQGSGYAADLKSTQNAIDKITNKVNSVIEKMNTQFTAVGKEFNHLEKRIENLNKKVDDGFLDIWTYNAELLVLLE
NERTLDYHDSNVKNLYEKVRNQLKNNAKEIGNGCFEFYHKCDNTCMESVKNGTYDYPKYSEEAKLNREKIDGVSAGSGGSGGSGGSGGSEKA
AKAEEEAARKMEELFKKHKIVAVLRANSVEEAIEKAVAVFAGGVHLIEITFTVPDADTVIKALSVLKEKGAIIGAGTVTSVEQCRKAVESGAE
FIVSPHLDEEISQFCKEKGVFYMPGVMTPTELVKAMKLGHTILKLFPGEVVGPQFVKAMKGPFPNVKFVPTGGVNLDNVCEWFKAGVLAVGV
GSALVKGTPDEVREKAKAFVEKIRGCTELEEQKLISEEDLHHHHHH

>H1MI15-foldon trimer (A/Michigan/45/2015 HA 1-676 Y98F FAH)
MDSKGSSQKGSRLLLLLVVSNLLLPQGVLADTLCIGYHANNSTDTVDTVLEKNVTVTHSVNLLEDKHNGKLCKLRGVAPLHLGKCNIAGWIL
GNPECESLSTASSWSYIVETSNSDNGTCFPGDFINYEELREQLSSVSSFERFEIFPKTSSWPNHDSNKGVTAACPHAGAKSFYKNLIWLVKK
GNSYPKLNQSYINDKGKEVLVLWGIHHPSTTADQQSLYQNADAYVFVGTSRYSKKFKPEIATRPKVRDQEGRMNYYWTLVEPGDKITFEATG
NLVVPRYAFTMERNAGSGIIISDTPVHDCNTTCQTPEGAINTSLPFQNIHPITIGKCPKYVKSTKLRLATGLRNVPSIQSRGLFGAIAGFIE
GGWTGMVDGWYGYHHQNEQGSGYAADLKSTQNAIDKITNKVNSVIEKMNTQFTAVGKEFNHLEKRIENLNKKVDDGFLDIWTYNAELLVLLE
NERTLDYHDSNVKNLYEKVRNQLKNNAKEIGNGCFEFYHKCDNTCMESVKNGTYDYPKYSEEAKLNREKIDGVGSGYIPEAPRDGQAYVRKD
GEWVLLSTFLGSGLNDIFEAQKIEWHEGHHHHHH

>H1MI15-1na0C3_int2 trimer (A/Michigan/45/2015 HA 1-676 Y98F no lkr 1na0C3_int2.SA.WELQ-H)
MKAILVVLLYTFTTANADTLCIGYHANNSTDTVDTVLEKNVTVTHSVNLLEDKHNGKLCKLRGVAPLHLGKCNIAGWILGNPECESLSTASS
WSYIVETSNSDNGTCFPGDFINYEELREQLSSVSSFERFEIFPKTSSWPNHDSNKGVTAACPHAGAKSFYKNLIWLVKKGNSYPKLNQSYIN
DKGKEVLVLWGIHHPSTTADQQSLYQNADAYVFVGTSRYSKKFKPEIATRPKVRDQEGRMNYYWTLVEPGDKITFEATGNLVVPRYAFTMER
NAGSGIIISDTPVHDCNTTCQTPEGAINTSLPFQNIHPITIGKCPKYVKSTKLRLATGLRNVPSIQSRGLFGAIAGFIEGGWTGMVDGWYGY
HHQNEQGSGYAADLKSTQNAIDKITNKVNSVIEKMNTQFTAVGKEFNHLEKRIENLNKKVDDGFLDIWTYNAELLVLLENERTLDYHDSNVK
NLYEKVRNQLKNNAKEIGNGCFEFYHKCDNTCMESVKNGTYDYPKYSEEAKLNREKIDGVSAEEAELAYLLGELAYKLGEYRIAIRAYRIAL
KRDPNNAEAWYNLGNAYYKQGDYDEAIEYYQKALELDPNNAEAWYNLGNAYYKQGDYDEAIEYYQKALELDPNNAEAKQNLGNAKQKQGGGW
ELQHHHHHH

>SARS-CoV-2_RBD-I53-50A trimer (16GS linker, using wild type RBD from Wuhan-Hu-1)
MGILPSPGMPALLSLVSLLSVLLMGCVAETGTRFPNITNLCPFGEVFNATRFASVYAWNRKRISNCVADYSVLYNSASFSTFKCYGVSPTKL
NDLCFTNVYADSFVIRGDEVRQIAPGQTGKIADYNYKLPDDFTGCVIAWNSNNLDSKVGGNYNYLYRLFRKSNLKPFERDISTEIYQAGSTP
CNGVEGFNCYFPLQSYGFQPTNGVGYQPYRVVVLSFELLHAPATVCGPKKSTGGSGGSGSGGSGGSGSEKAAKAEEAARKMEELFKKHKIVA
VLRANSVEEAIEKAVAVFAGGVHLIEITFTVPDADTVIKALSVLKEKGAIIGAGTVTSVEQARKAVESGAEFIVSPHLDEEISQFAKEKGVF
YMPGVMTPTELVKAMKLGHTILKLFPGEVVGPQFVKAMKGPFPNVKFVPTGGVNLDNVAEWFKAGVLAVGVGSALVKGTPDEVREKAKAFVE
KIRGATEGGSHHHHHHHH

>SARS-CoV-2_SpikeHexaPro-foldon trimer
MFVFLVLLPLVSSQCVNLTTRTQLPPAYTNSFTRGVYYPDKVFRSSVLHSTQDLFLPFFSNVTWFHAIHVSGTNGTKRFDNPVLPFNDGVYF
ASTEKSNIIRGWIFGTTLDSKTQSLLIVNNATNVVIKVCEFQFCNDPFLGVYYHKNNKSWMESEFRVYSSANNCTFEYVSQPFLMDLEGKQG
NFKNLREFVFKNIDGYFKIYSKHTPINLVRDLPQGFSALEPLVDLPIGINITRFQTLLALHRSYLTPGDSSSGWTAGAAAYYVGYLQPRTFL
LKYNENGTITDAVDCALDPLSETKCTLKSFTVEKGIYQTSNFRVQPTESIVRFPNITNLCPFGEVFNATRFASVYAWNRKRISNCVADYSVL
YNSASFSTFKCYGVSPTKLNDLCFTNVYADSFVIRGDEVRQIAPGQTGKIADYNYKLPDDFTGCVIAWNSNNLDSKVGGNYNYLYRLFRKSN
LKPFERDISTEIYQAGSTPCNGVEGFNCYFPLQSYGFQPTNGVGYQPYRVVVLSFELLHAPATVCGPKKSTNLVKNKCVNFNFNGLTGTGVL
TESNKKFLPFQQFGRDIADTTDAVRDPQTLEILDITPCSFGGVSVITPGTNTSNQVAVLYQDVNCTEVPVAIHADQLTPTWRVYSTGSNVFQ
TRAGCLIGAEHVNNSYECDIPIGAGICASYQTQTNSPGSASSVASQSIIAYTMSLGAENSVAYSNNSIAIPTNFTISVTTEILPVSMTKTSV
DCTMYICGDSTECSNLLLQYGSFCTQLNRALTGIAVEQDKNTQEVFAQVKQIYKTPPIKDFGGFNFSQILPDPSKPSKRSPIEDLLFNKVTL
ADAGFIKQYGDCLGDIAARDLICAQKFNGLTVLPPLLTDEMIAQYTSALLAGTITSGWTFGAGPALQIPFPMQMAYRFNGIGVTQNVLYENQ
KLIANQFNSAIGKIQDSLSSTPSALGKLQDVVNQNAQALNTLVKQLSSNFGAISSVLNDILSRLDPPEAEVQIDRLITGRLQSLQTYVTQQL
IRAAEIRASANLAATKMSECVLGQSKRVDFCGKGYHLMSFPQSAPHGVVFLHVTYVPAQEKNFTTAPAICHDGKAHFPREGVFVSNGTHWFV
TQRNFYEPQIITTDNTFVSGNCDVVIGIVNNTVYDPLQPELDSFKEELDKYFKNHTSPDVDLGDISGINASVVNIQKEIDRLNEVAKNLNES
LIDLQELGKYEQGSGYIPEAPRDGQAYVRKDGEWVLLSTFLGRSLEVLFQGPGHHHHHHHHSAWSHPQFEKGGGSGGGGSGGSAWSHPQFEK

>RSV-F_DS-Cav1-I53-50A trimer
MELLILKANAITTILTAVTFCFASGQNITEEFYQSTCSAVSKGYLSALRTGWYTSVITIELSNIKENKCNGTDAKVKLIKQELDKYKNAVTE
LQLLMQSTPATNNRARRELPRFMNYTLNNAKKTNVTLSKKRKRRFLGFLLGVGSAIASGVAVCKVLHLEGEVNKIKSALLSTNKAVVSLSNG
VSVLTFKVLDLKNYIDKQLLPILNKQSCSISNIETVIEFQQKNNRLLEITREFSVNAGVTTPVSTYMLTNSELLSLINDMPITNDQKKLMSN
NVQIVRQQSYSIMCIIKEEVLAYVVQLPLYGVIDTPCWKLHTSPLCTTNTKEGSNICLTRTDRGWYCDNAGSVSFFPQAETCKVQSNRVFCD
TMNSLTLPSEVNLCNVDIFNPKYDCKIMTSKTDVSSSVITSLGAIVSCYGKTKCTASNKNRGIIKTFSNGCDYVSNKGVDTVSVGNTLYYVN
KQEGKSLYVKGEPIINFYDPLVFPSDEFDASISQVNEKINQSLAFIRKSDELLGSGGSGSGSGGSEKAAKAEEAARKMEELFKKHKIVAVLR
ANSVEEAIEKAVAVFAGGVHLIEITFTVPDADTVIKALSVLKEKGAIIGAGTVTSVEQCRKAVESGAEFIVSPHLDEEISQFCKEKGVFYMP
GVMTPTELVKAMKLGHTILKLFPGEVVGPQFVKAMKGPFPNVKFVPTGGVNLDNVCEWFKAGVLAVGVGSALVKGTPDEVREKAKAFVEKIR
GCTELEHHHHHH

>RSV-F_DS-Cav1-foldon trimer
MELLILKANAITTILTAVTFCFASGQNITEEFYQSTCSAVSKGYLSALRTGWYTSVITIELSNIKENKCNGTDAKVKLIKQELDKYKNAVTE
LQLLMQSTPATNNRARRELPRFMNYTLNNAKKTNVTLSKKRKRRFLGFLLGVGSAIASGVAVCKVLHLEGEVNKIKSALLSTNKAVVSLSNG
VSVLTFKVLDLKNYIDKQLLPILNKQSCSISNIETVIEFQQKNNRLLEITREFSVNAGVTTPVSTYMLTNSELLSLINDMPITNDQKKLMSN
NVQIVRQQSYSIMCIIKEEVLAYVVQLPLYGVIDTPCWKLHTSPLCTTNTKEGSNICLTRTDRGWYCDNAGSVSFFPQAETCKVQSNRVFCD
TMNSLTLPSEVNLCNVDIFNPKYDCKIMTSKTDVSSSVITSLGAIVSCYGKTKCTASNKNRGIIKTFSNGCDYVSNKGVDTVSVGNTLYYVN

KQEGKSLYVKGEPIINFYDPLVFPSDEFDASISQVNEKINQSLAFIRKSDELLSAIGGYIPEAPRDGQAYVRKDGEWVLLSTFLENLYFQSS
AWSHPQFEKGGGSGGGSGGSAWSHPQFEKGSGSGSGLNDIFEAQKIEWHEGSGSGSHHHHHHHH

>HIVenv(AMC009)-I53-50A trimer

ADKLWVTVYYGVPVWKDACTTLFCASDAKAYDTEKRNVWATHCCVPTDPNPQEVVLENVTENFNMWKNDMVEQMHEDIISLWDQSLKPCVKL
TPLCVTLNCTDYVGNATNASTTNATGGIGGTVERGEIKNCSFNITTSLRDKVQKEYALFYKLDIVPIDNDNTNNTYRLINCNTTVIKQACPK
VSFEPIPIHYCAPAGFAILKCNDKKFNGTGPCTNVSTVQCTHGIRPVVSTQLLLNGSLAEKEVIIRSQNFTNNAKVIIVQLNESVVINCTRP
NNNTVKSIHIAPGQWFYYTGAIIGDIRQAHCNISRVKWNNTLKQIATKLREQFKNKTIAFNQSSGGDPEIVMHSFNCGGEFFYCNTTQLFNS
TWNDTEVSNYTDITHITLPCRIKQIINMWQRVGQAMYAPPIRGQIRCSSNITGLLLTRDGGSNENKTSETETFRPAGGDMRDNWRSELYKYK
VVKIEPLGVAPTRCKRRVVQRRRRRRAVGAIGAVSLGFLGAAGSTMGAASMTLTVQARQLLSGIVQQQNNCLRAPECQQHMLKDTHWGIKQL
QARVLAVEHYLRDQQLLGIWGCSGKLICCTAVPWNNTWSNRSLDMIWNNMTWIEWEREIDNYTGLIYNLLEESQNQQEKNEQELLELDGSGG
SGGSGGSGGSEKAAKAEEAARKMEELFKKHKIVAVLRANSVEEAIEKAVAVFAGGVHLIEITFTVPDADTVIKALSVLKEKGAIIGAGTVTS
VEQCRKAVESGAEFIVSPHLDEEISQFCKEKGVFYMPGVMTPTELVKAMKLGHTILKLFPGEVVGPQFVKAMKGPFPNVKFVPTGGVNLDNV
CEWFKAGVLAVGVGSALVKGTPDEVREKAKAFVEKIRGCTE

>HIVenv(ConM)-I53-50A trimer

AENLWVTVYYGVPVWKDAETTLFCASDAKAYDTEKRNVWATHCCVPTDPNPQEIVLENVTENFNMWKNNMVEQMHTDIISLWDQSLKPCVKL
TPLCVTLNCTDVNATNNTTNNEEIKNCSFNITTELRDKKKKVYALFYKLDVVPIDDNNSYRLINCNTSAITQACPKVSFEPIPIHYCAPAGF
AILKCNDKKFNGTGPCKNVSTVQCTHGIKPVVSTQLLLNGSLAEEEIIIRSENITNNAKTIIVQLNESVEINCTRPNNNTRKSIRIGPGQWF
YATGDIIGDIRQAHCNISRTKWNKTLQQVAKKLREHFNKTIIFNPSSGGDLEITTHSFNCGGEFFYCNTSELFNSTWNGTNNTITLPCRIKQ
IINMWQRVGQAMYAPPIEGKIRCTSNITGLLLTRDGGNNNTETFRPGGGDMRDNWRSELYKYKVVKIEPLGVAPTRCKRRVVERRRRRRAVG
IGAVFLGFLGAAGSTMGAASMTLTVQARNLLSGIVQQQSNLLRAPECQQHLLQLTVWGIKQLQARVLAVERYLKDQQLLGIWGCSGKLICCT
NVPWNSSWSNKSQDEIWDNMTWMEWDKEINNYTDIIYSLIEESQNQQEKNEQELLALDGSGGSGGSGGSGGSEKAAKAEEAARKMEELFKKH
KIVAVLRANSVEEAIEKAVAVFAGGVHLIEITFTVPDADTVIKALSVLKEKGAIIGAGTVTSVEQCRKAVESGAEFIVSPHLDEEISQFCKE
KGVFYMPGVMTPTELVKAMKLGHTILKLFPGEVVGPQFVKAMKGPFPNVKFVPTGGVNLDNVCEWFKAGVLAVGVGSALVKGTPDEVREKAK
AFVEKIRGCTE

>HIVenv(AMC009)-8xHis trimer

ADKLWVTVYYGVPVWKDACTTLFCASDAKAYDTEKRNVWATHCCVPTDPNPQEVVLENVTENFNMWKNDMVEQMHEDIISLWDQSLKPCVKL
TPLCVTLNCTDYVGNATNASTTNATGGIGGTVERGEIKNCSFNITTSLRDKVQKEYALFYKLDIVPIDNDNTNNTYRLINCNTTVIKQACPK
VSFEPIPIHYCAPAGFAILKCNDKKFNGTGPCTNVSTVQCTHGIRPVVSTQLLLNGSLAEKEVIIRSQNFTNNAKVIIVQLNESVVINCTRP
NNNTVKSIHIAPGQWFYYTGAIIGDIRQAHCNISRVKWNNTLKQIATKLREQFKNKTIAFNQSSGGDPEIVMHSFNCGGEFFYCNTTQLFNS
TWNDTEVSNYTDITHITLPCRIKQIINMWQRVGQAMYAPPIRGQIRCSSNITGLLLTRDGGSNENKTSETETFRPAGGDMRDNWRSELYKYK
VVKIEPLGVAPTRCKRRVVQRRRRRRAVGAIGAVSLGFLGAAGSTMGAASMTLTVQARQLLSGIVQQQNNCLRAPECQQHMLKDTHWGIKQL
QARVLAVEHYLRDQQLLGIWGCSGKLICCTAVPWNNTWSNRSLDMIWNNMTWIEWEREIDNYTGLIYNLLEESQNQQEKNEQELLELDGSGS
GGSGHHHHHHHH

>HIVenv(ConM)-8xHis trimer

AENLWVTVYYGVPVWKDAETTLFCASDAKAYDTEKRNVWATHCCVPTDPNPQEIVLENVTENFNMWKNNMVEQMHTDIISLWDQSLKPCVKL
TPLCVTLNCTDVNATNNTTNNEEIKNCSFNITTELRDKKKKVYALFYKLDVVPIDDNNSYRLINCNTSAITQACPKVSFEPIPIHYCAPAGF
AILKCNDKKFNGTGPCKNVSTVQCTHGIKPVVSTQLLLNGSLAEEEIIIRSENITNNAKTIIVQLNESVEINCTRPNNNTRKSIRIGPGQWF
YATGDIIGDIRQAHCNISRTKWNKTLQQVAKKLREHFNKTIIFNPSSGGDLEITTHSFNCGGEFFYCNTSELFNSTWNGTNNTITLPCRIKQ
IINMWQRVGQAMYAPPIEGKIRCTSNITGLLLTRDGGNNNTETFRPGGGDMRDNWRSELYKYKVVKIEPLGVAPTRCKRRVVERRRRRRAVG
IGAVFLGFLGAAGSTMGAASMTLTVQARNLLSGIVQQQSNLLRAPECQQHLLQLTVWGIKQLQARVLAVERYLKDQQLLGIWGCSGKLICCT
NVPWNSSWSNKSQDEIWDNMTWMEWDKEINNYTDIIYSLIEESQNQQEKNEQELLALDGSGSGGSGHHHHHHHH

Data S1. Rosetta XML code for protein glycosylation, Related to STAR Methods

```
<ROSETTASCRIPTS>
  <SCOREFXNS>
       <ScoreFunction name="sfx_clean" weights="beta" symmetric="0" /> //function to obtain a score
  </SCOREFXNS>
```

```
  <RESIDUE_SELECTORS>
       <Index name="select_i_enh0" resnums="%%resi_enh0%%" /> //select the residue(s) to glycosylate (these
residues are a "non-enhanced" sequon)
              Not name="not_resis" selector="select" /> //all other residues not selected
       <Index name="select_i_enh1" resnums="%%resi_enh1%%" /> //select the residue(s) to glycosylate (these
residues are an "enhanced" sequon)
              Not name="not_resis" selector="select" /> //all other residues not selected
       Index name="select_i-2" resnums="%%enhresi%%" /> //select the residue i-2 from the N-linked
glycosylation site
       Index name="select_i_score" resnums="%%iresi%%" /> //select Asn residue i that is N-liked
glycosylated to get its score
  </RESIDUE_SELECTORS>
  <FILTERS>
       EnergyPerResidue name="total_energy_per_res_filter_i" scorefxn="sfx_clean" energy_cutoff="10000"
resnums="%%iresi%%" /> // tests the energy of a particular residue, or interface, or whole protein, or a set
of residues; energy must be less than 10000
       EnergyPerResidue name="total_energy_per_res_filter_i-2" scorefxn="sfx_clean" energy_cutoff="10000"
resnums="%%enhresi%%" /> // tests the energy of a particular residue, or interface, or whole protein, or a
set of residues; energy must be less than 10000
       EnergyPerResidue name="fa_atr_per_res_filter" scorefxn="sfx_clean" score_type="fa_atr"
energy_cutoff="10000" resnums="%%resi%%" />
       EnergyPerResidue name="fa_rep_per_res_filter" scorefxn="sfx_clean" score_type="fa_rep"
energy_cutoff="10000" resnums="%%resi%%" />
       EnergyPerResidue name="fa_dun_per_res_filter" scorefxn="sfx_clean" score_type="fa_dun"
energy_cutoff="10000" resnums="%%resi%%" />
       EnergyPerResidue name="fa_elec_per_res_filter" scorefxn="sfx_clean" score_type="fa_elec"
energy_cutoff="10000" resnums="%%resi%%" />
  </FILTERS>

  <MOVERS>
       <CreateGlycanSequonMover name="create_motif_enh0" residue_selector="select_i_enh0"
basic_enhanced_n_sequon="0" design_x_positions="1" pack_neighbors="1" scorefxn="sfx_clean" />
       <CreateGlycanSequonMover name="create_motif_enh1" residue_selector="select_i_enh1"
basic_enhanced_n_sequon="1" design_x_positions="1" pack_neighbors="1" scorefxn="sfx_clean" />
       <SimpleGlycosylateMover name="glycosylate_enh0" residue_selector="select_i_enh0"
glycosylation="a-D-Manp-(1->3)-[a-D-Manp-(1->3)-[a-D-Manp-(1->6)]-a-D-Manp-(1->6)]-[b-d-GlcpNAc-(1->4)]-b-D-M
anp-(1->4)-b-D-GlcpNAc-(1->4)-[a-L-Fucp-(1->6)]-b-D-GlcpNAc-" strip_existing="1" />
       <SimpleGlycosylateMover name="glycosylate_enh1" residue_selector="select_i_enh1"
glycosylation="a-D-Manp-(1->3)-[a-D-Manp-(1->3)-[a-D-Manp-(1->6)]-a-D-Manp-(1->6)]-[b-d-GlcpNAc-(1->4)]-b-D-M
anp-(1->4)-b-D-GlcpNAc-(1->4)-[a-L-Fucp-(1->6)]-b-D-GlcpNAc-" strip_existing="1" />
       <SymMinMover name="bb_min" scorefxn="sfx_clean" bb="1" chi="1" jump="0"
type="lbfgs_armijo_nonmonotone" tolerance="0.005" max_iter="100" />
       <GlycanTreeModeler name="tree_modeler" quench_mode="false" rounds="1" layer_size="1" window_size="0"
hybrid_protocol="1" shear="1" use_gaussian_sampling="1" glycan_sampler_rounds="150" />
       GlycanSampler name="tree_sampler" kt="0" rounds="1" pack_distance="5.0" scorefxn="sfx_clean" />
       GlycanRelaxMover name="basic_relax" />
       GlycanTreeRelax name="tree_relax" quench_mode="false" rounds="1" layer_size="2" window_size="1"/>
  </MOVERS>

  <PROTOCOLS>
       // wiggle backbone to loosen up a bit
       <Add mover_name="bb_min" />

       // generate sequon (enhanced or not) and add glycan
       <Add mover_name="create_motif_enh0" />
       <Add mover_name="create_motif_enh1" />

       // wiggle backbone to loosen up a bit
       <Add mover_name="bb_min" />

       // filter to extract energy of residues
       Add filter_name="total_energy_per_res_filter_i" />
       Add filter_name="total_energy_per_res_filter_i-2" />
       Add filter_name="fa_atr_per_res_filter" />
```

```
      Add filter_name="fa_rep_per_res_filter" />
      Add filter_name="fa_dun_per_res_filter" />
      Add filter_name="fa_elec_per_res_filter" />

      // add glycan and model glycan
      <Add mover_name="glycosylate_enh0" />
      <Add mover_name="glycosylate_enh1" />
      <Add mover_name="tree_modeler" />
      Add mover_name="tree_sampler" />
      Add mover_name="basic_relax" />
      Add mover_name="tree_relax" />
  </PROTOCOLS>
</ROSETTASCRIPTS>
```
