# Supplementary material for: Antigen- and scaffold-specific antibody responses to protein nanoparticle immunogens
Source: Cell Rep Med. 2022 Sep 26;3(10):100780. doi: 10.1016/j.xcrm.2022.100780 (PMC9589121; doi:10.1016/j.xcrm.2022.100780)
Supplement: Document S2. Article plus supplemental information [file mmc2.pdf]

Cell Reports
Medicine

Article

# Antigen- and scaffold-specific antibody responses to protein nanoparticle immunogens

## Graphical abstract

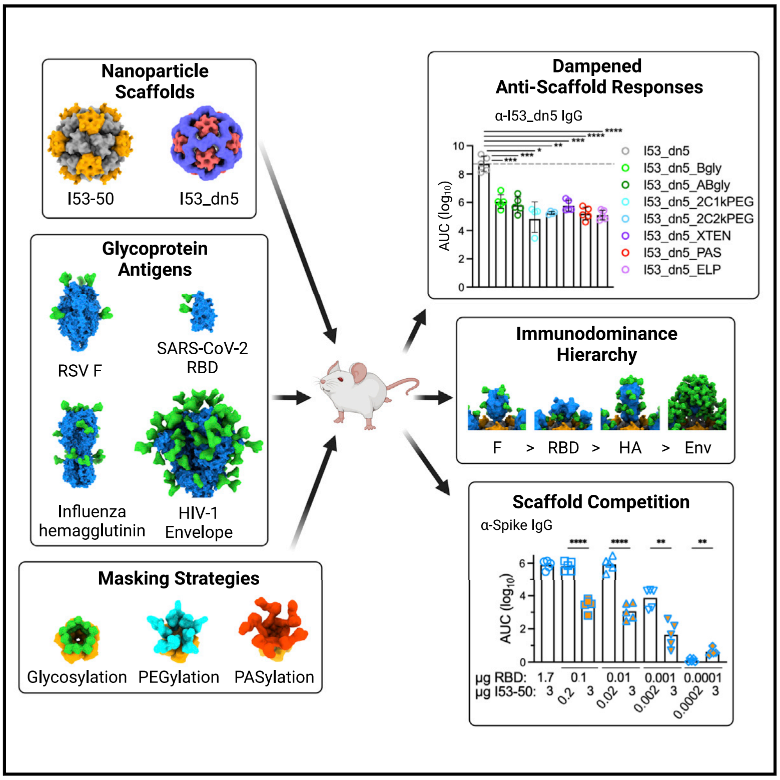


## Highlights

- Physical masking of scaffolds does not enhance antigen (Ag)-specific antibody responses
- In a series of NP immunogens, only HIV-1 Env is subdominant to the scaffold
- Scaffold-specific antibody responses can competitively inhibit Ag-specific responses
- Immunodominant Ag responses are unlikely to be suppressed by scaffold responses

## Authors

John C. Kraft, Minh N. Pham, Laila Shehata, ..., Marion Pepper, Rogier W. Sanders, Neil P. King

## Correspondence

neil@ipd.uw.edu

## In brief

Kraft et al. define relationships between anti-scaffold and antigen-specific antibody responses elicited by protein nanoparticle immunogens. Overall, anti-scaffold antibody responses are unlikely to suppress antigen-specific antibody responses for protein nanoparticle immunogens in which the antigen is immunodominant over the scaffold.

Kraft et al., 2022, Cell Reports Medicine *3*, 100780
October 18, 2022 © 2022 The Author(s).
https://doi.org/10.1016/j.xcrm.2022.100780

CellPress

Cell Reports Medicine

CellPress
OPEN ACCESS

Article

# Antigen- and scaffold-specific antibody responses to protein nanoparticle immunogens

John C. Kraft,[1,2] Minh N. Pham,[1,2] Laila Shehata,[3] Mitch Brinkkemper,[5] Seyhan Boyoglu-Barnum,[4] Kaitlin R. Sprouse,[1] Alexandra C. Walls,[1,8] Suna Cheng,[1,2] Mike Murphy,[1,2] Deleah Pettie,[1,2] Maggie Ahlrichs,[1,2] Claire Sydeman,[1,2] Max Johnson,[1,2] Alyssa Blackstone,[1,2] Daniel Ellis,[1,2] Rashmi Ravichandran,[1,2] Brooke Fiala,[1,2] Samuel Wrenn,[1,2] Marcos Miranda,[1,2] Kwinten Sliepen,[5] Philip J.M. Brouwer,[5] Aleksandar Antanasijevic,[6] David Veesler,[1,8] Andrew B. Ward,[6] Masaru Kanekiyo,[4] Marion Pepper,[3] Rogier W. Sanders,[5,7] and Neil P. King[1,2,9,*]

[1]Department of Biochemistry, University of Washington, Seattle, WA 98195, USA
[2]Institute for Protein Design, University of Washington, Seattle, WA 98195, USA
[3]Department of Immunology, University of Washington, Seattle, WA 98195, USA
[4]Vaccine Research Center, National Institute of Allergy and Infectious Diseases, National Institutes of Health, Bethesda, MD 20892, USA
[5]Department of Medical Microbiology, Amsterdam UMC, University of Amsterdam, Amsterdam Infection and Immunity Institute, 1105 AZ Amsterdam, the Netherlands
[6]Department of Integrative Structural and Computational Biology, The Scripps Research Institute, La Jolla, CA 92037, USA
[7]Department of Microbiology and Immunology, Weill Medical College of Cornell University, New York, NY 10021, USA
[8]Howard Hughes Medical Institute, Seattle, WA 98195, USA
[9]Lead contact
*Correspondence: neil@ipd.uw.edu
https://doi.org/10.1016/j.xcrm.2022.100780

## SUMMARY

Protein nanoparticle scaffolds are increasingly used in next-generation vaccine designs, and several have established records of clinical safety and efficacy. Yet the rules for how immune responses specific to nanoparticle scaffolds affect the immunogenicity of displayed antigens have not been established. Here we define relationships between anti-scaffold and antigen-specific antibody responses elicited by protein nanoparticle immunogens. We report that dampening anti-scaffold responses by physical masking does not enhance antigen-specific antibody responses. In a series of immunogens that all use the same nanoparticle scaffold but display four different antigens, only HIV-1 envelope glycoprotein (Env) is subdominant to the scaffold. However, we also demonstrate that scaffold-specific antibody responses can competitively inhibit antigen-specific responses when the scaffold is provided in excess. Overall, our results suggest that anti-scaffold antibody responses are unlikely to suppress antigen-specific antibody responses for protein nanoparticle immunogens in which the antigen is immunodominant over the scaffold.

## INTRODUCTION

We are currently in the midst of a technological revolution in the field of vaccinology. Three general capabilities are being combined to provide information to the immune system that potently and safely stimulates immunity: (1) highly adaptable vaccine delivery platforms such as mRNA[2,3] and self-assembling protein nanoparticle scaffolds,[4–11] (2) high-throughput structural studies of protective monoclonal antibodies (mAbs) interacting with their target antigens that identify key targets for antigen design (i.e., "reverse vaccinology 2.0"),[12,13] and (3) increasingly sophisticated tools and platforms for "structure-based vaccine design": the design of novel antigens and immunogens inspired by these structural studies.[14–21] The successful integration of these technologies and approaches enabled the development of highly efficacious severe acute respiratory syndrome coronavirus 2 (SARS-CoV-2) vaccines in less than 1 year from the identification of the virus,[22–24] an achievement unprecedented in the history of vaccinology.

Self-assembling protein nanoparticles are ideal scaffolds for increasing the magnitude and quality of antibody responses through multivalent antigen presentation.[5,8,16,25,26] Recently, computationally designed two-component nanoparticles[27–30] have emerged as a versatile platform for multimerizing complex oligomeric viral glycoprotein antigens. Two-component nanoparticle vaccines for respiratory syncytial virus (RSV),[8] influenza,[10] and SARS-CoV-2[9,31] have rapidly advanced to clinical trials; the latter recently met its primary endpoint in a phase 3 clinical trial[32] and was approved by the Korean Ministry of Food and Drug Safety for use in individuals 18 years and older. In addition to the often highly potent antigen-specific responses, antibodies are also elicited against the protein nanoparticle scaffolds themselves. It remains unclear how these anti-scaffold responses shape the immunogenicity of the displayed antigens, especially in the context of repeated immunizations: do they interfere with antigen-specific responses, play a beneficial role, or are they largely irrelevant?


This is an open access article under the CC BY license (http://creativecommons.org/licenses/by/4.0/).

In general, for many class I viral glycoprotein antigens displayed on protein nanoparticles (with the exception of HIV-1 Env trimers[33,34]), antigen-specific antibody titers are higher than scaffold-specific titers. For instance, hemagglutinin (HA) displayed on ferritin elicited higher anti-HA titers than anti-ferritin titers,[5] and ferritin-specific T follicular helper (Tfh) cell responses were minimal compared with HA-specific Tfh cell responses.[35] Similarly, DS-Cav1-I53-50, a two-component nanoparticle vaccine for RSV, elicited antigen-specific antibody titers 2-fold higher than the anti-scaffold titers; furthermore, pre-immunization with the bare I53-50 nanoparticle scaffold did not diminish antigen-specific (i.e., binding) or viral neutralization titers, suggesting that pre-existing anti-scaffold immunity had no adverse effects on antigen-specific immune responses.[8] Moreover, in non-human primates (NHPs) given two doses of a two-component nanoparticle displaying 60 copies of the SARS-CoV-2 spike receptor-binding domain (RBD), post-boost anti-scaffold antibody titers positively correlated with anti-spike antibody titers and viral neutralizing activity, suggesting that anti-scaffold antibody responses did not interfere with antigen-specific antibody responses.[36] Clinical studies of RTS,S, a protein nanoparticle vaccine for malaria that induces strong antibody responses against the hepatitis B surface antigen scaffold,[4,37] found that pre-existing anti-scaffold antibody titers either did not significantly affect[37–39] or actually increased[40] the levels of antigen-specific antibody titers after immunization. In contrast, pre-existing anti-scaffold responses for vaccine nanoparticles displaying subdominant HIV-1 Env have been reported to interfere with elicitation of antiviral immunity. In a study that compared the immunogenicity of HIV-1 Env-ferritin nanoparticles formulated with several different adjuvants, post-prime anti-ferritin antibody titers and post-boost neutralizing antibody titers were negatively correlated, suggesting interference of pre-existing anti-ferritin responses with antiviral responses.[34] However, adjuvant-mediated perturbation of immunodominant epitopes on Env and inconsistent doses of Env (and thus ferritin) among the study groups could have confounded this negative association.

It has been proposed that the potential interference of anti-scaffold responses with antigen-specific responses could be mitigated through B cell epitope masking strategies such as glycosylation or PEGylation that would dampen anti-scaffold responses and potentially redirect immune responses to the antigen.[41,42] However, in practice, glycan masking of epitopes on protein immunogens does not appear to substantially increase the magnitude of immune responses toward target epitopes. Instead, responses are reduced against glycan-masked surfaces, which increases the proportion—but not the absolute amount—of on-target antibody responses.[43–49] Similarly, masking a heterologous trimerization domain with four N-linked glycans that was fused to HIV-1 Env and influenza HA trimers strongly reduced the anti-trimerization domain antibody responses but had no impact on the antibody titers against Env or HA.[46] This is in contrast to other masking studies that operate solely on the antigen to shield off-target immunodominant surfaces[43,44,47–54] or subregions within a target epitope.[55] As for PEGylation, an approach called "protect, modify, deprotect" used poly-ethylene glycol (PEG) to focus immune responses to the conserved stem region of HA and boost cross-reactive antibody titers: antibody is first bound to the desired epitope, the immunogen is non-specifically PEGylated to reduce immunogenicity of all other surfaces, and then the antibody is removed to expose the desired epitope.[56] Although much work has focused on masking antigens, protein nanoparticles have also been glycosylated[57] and PEGylated (e.g., cowpea mosaic virus,[58,59] adenovirus,[60–62] ferritin,[63] dihydrolipoamide acetyltransferase complex,[64] and Qβ[65]), but not in the context of a displayed antigen. Therefore, it remains unknown if sterically hindering B cell receptor (BCR) accessibility via scaffold masking can provide an antigen-specific immunofocusing benefit.

Here, we address questions about the role of anti-scaffold responses in shaping the immunogenicity of protein nanoparticle immunogens through (1) physically masking the nanoparticle scaffold using three different approaches, (2) studying how antigen immunodominance impacts anti-scaffold responses, and (3) assessing immunogenic competition between the displayed antigen and nanoparticle scaffold. We found that while scaffold masking did not enhance antigen-specific antibody responses, the relative immunodominance and dose of the nanoparticle scaffold and antigen are key factors in shaping antigen-specific immunogenicity for protein nanoparticle immunogens.

## RESULTS

### Design and characterization of HA-I53_dn5 nanoparticle immunogens with a glycosylated, PEGylated, or PASylated nanoparticle scaffold

To test the impact of masking the nanoparticle scaffold on antigen-specific antibody responses, we selected as our model scaffold the I53_dn5 protein nanoparticle[30] due to its robust self-assembly and stability and its use as the scaffold for a mosaic nanoparticle influenza vaccine in clinical testing.[10,66] I53_dn5 is a 25-nm, two-component nanoparticle with icosahedral symmetry constructed from 12 pentameric and 20 trimeric building blocks. We compared three different approaches to masking I53_dn5 surfaces: glycosylation, PEGylation, and genetic fusion of unstructured polypeptides rich in Pro, Ala, and Ser (i.e., PASylation[67]).

To introduce NxT/S potential N-linked glycosylation sites (PNGS) into the exposed surfaces of the I53_dn5A pentamer and the I53_dn5B trimer, we used a custom "sugarcoat" protocol that we recently developed[1] as part of the Rosetta macromolecular modeling and design software.[68,69] Sequences corresponding to design models containing a single inserted NxT/S sequon, modeled with and without a Man9 glycan tree present, that had a Rosetta "total_energy" <500, as well as <0.25 Å and <0.40 Å backbone (Cα) root-mean-square deviation (RMSD) compared with the parent I53_dn5A and I53_dn5B design models, respectively, were tested for protein expression and glycosylation (Figures S1A and S1B). Reducing western blot analysis of cell culture supernatants showed variable expression and glycosylation for nine I53_dn5A and sixteen I53_dn5B variants that contained a single NxT/S sequon (Figure S1C). For I53_dn5A, six variants that expressed better than the parent sequence and/or exhibited migration indicating glycosylation were considered further. For I53_dn5B, three variants exhibited partial glycosylation. Next, variants that contained combinations

CellPress
OPEN ACCESS

of these validated single NxT/S PNGS were tested for expression, glycosylation, and nanoparticle assembly competency. A single variant of I53_dn5A (I53_dn5A_2gly; 84-NDT-86, 118-NST-120) and I53_dn5B (I53_dn5B_2gly; 32-YDNLT-36, 89-NAT-91), each with two glycans per protomer, exhibited the most efficient expression, glycosylation, and nanoparticle assembly based on reducing western blot analysis of cell culture supernatants and size exclusion chromatography (SEC) (Figures S1D and S2A). These two glycosylated variants also assembled with each other to form glycosylated I53_dn5 particles (I53_dn5_ABgly) bearing 240 glycans on the exterior surface, although with somewhat reduced efficiency (Figures S2A and S2B). To produce HA-bearing nanoparticle immunogens with a glycosylated scaffold, I53_dn5A_2gly was mixed with a genetic fusion of an H1 HA (H1/A/Michigan/45/2015, "MI15") and the I53_dn5B trimer (HA-I53_dn5B) *in vitro* with a slight molar excess of the trimeric components (Figure 1A), and it was purified via SEC (Figure 1D). PNGase F digestion of N-linked glycans followed by reducing SDS-PAGE analysis verified glycosylation of the I53_dn5A_2gly component of the SEC-purified HA-I53_dn5_Agly particles (Figure 1G). Dynamic light scattering (DLS) (Figures 1D and S2J) and negative stain transmission electron microscopy (nsTEM) (Figure 1J) analysis verified assembly into monodisperse nanoparticle immunogens with the intended icosahedral architecture.

To specifically couple PEG to precise locations on the I53_dn5 nanoparticle surface, we designed I53_dn5A pentamer variants with surface-exposed cysteines to enable PEG-maleimide conjugation.[70] We did not design I53_dn5B trimer cysteine knock-ins due to the potential for coupled PEG to occlude membrane-proximal epitopes on fused antigens (e.g., the conserved HA stem region). Seven I53_dn5A cysteine knock-ins were designed with either one or two surface-exposed cysteines per protomer. Two designs (I53_dn5A_D120C, I53_dn5A_S84C_D120C) had acceptable expression (>100 mg/L of bacterial expression media); did not aggregate during 4°C storage; coupled efficiently to 1, 2, and 5 kDa PEG; and assembled into PEGylated I53_dn5 nanoparticles based on SEC purification (Figures S2C and S2I). However, I53_dn5A_D120C pentamers with a single conjugated 5 kDa PEG per subunit did not efficiently assemble with HA-bearing I53_dn5B trimers (Figure S2G). By contrast, PEGylated HA-I53_dn5 immunogens with 1 or 2 kDa PEG coupled to the ten thiol groups on each I53_dn5A_S84C_D120C pentamer (Figure 1B) were found to form monodisperse particles based on SEC, DLS, and nsTEM (Figures 1E, 1K, and S2J), and these were carried forward for *in vivo* testing.

An alternative physical masking approach to PEGylation is the genetic fusion of hydrophilic unstructured polypeptides, such as XTENylation and PASylation[67,71,72]; these have been expressed on ferritin to extend its circulation time *in vivo.*[73,74] To express XTEN and proline/alanine-rich sequence (PAS) polypeptides on the outer surface of the I53_dn5A pentamer, we first designed a circularly permuted variant of I53_dn5A, called I53_dn5Acp7, with the N and C termini both facing outward. I53_dn5 nanoparticle formation was observed via SEC when XTEN, PAS, and another unstructured polypeptide known as ELP[75] were fused to the C terminus of the I53_dn5Acp7 pentamer (Figure S2E). PASylated I53_dn5Acp7 (I53_dn5A_PAS) assembled, albeit inefficiently, with HA-I53_dn5B trimers (Figure 1C) to form monodisperse nanoparticle immunogens based on SEC, DLS, and nsTEM, with nsTEM revealing some presence of unassembled components in the nanoparticle sample (Figures 1F, 1I, 1L,and S2J), and they were used for *in vivo* studies.

Overall, based on SEC and SDS-PAGE analysis, the larger PAS polypeptide and PEG molecules impeded efficient nanoparticle assembly (Figures 1F, 1I, S2C–S2H) more than smaller PEG and glycans did (Figures 1D, 1E, 1G, 1H, S2A–S2D, and S2G–S2H). This trend of less efficient nanoparticle assembly with bulkier masking groups is consistent with the estimated molecular weight of each masking moiety on the I53_dn5A pentamer (and hydrodynamic diameter of assembled I53_dn5 nanoparticles with the respective masked I53_dn5A): 9-mannose N-linked glycan, 1,884 Da (31 nm); 2 kDa PEG, 2,000 Da (31 nm); 63 amino acid PAS polypeptide, 5,187 Da (38 nm), suggesting that the presence of larger, flexible masking agents interferes with nanoparticle assembly. However, we note that very large glycoprotein antigens such as HIV-1 Env and SARS-CoV-2 spike (approximately 120 and 170 kDa per monomer including glycans, respectively) are able to efficiently assemble into I53_dn5 and similarly sized I53-50 nanoparticles,[31,33] presumably because they are not quite so dynamic.

## Masking the I53_dn5 nanoparticle scaffold does not enhance anti-HA antibody responses

We first tested how effectively these three different surface masking strategies dampen antibody responses against the I53_dn5 nanoparticle without any viral glycoprotein antigen present. After three immunizations of 0.6 μg protein adjuvanted with AddaVax, the presence of glycans on either the I53_dn5B trimer (I53_dn5_Bgly) or both the I53_dn5A pentamer and I53_dn5B trimer (I53_dn5_ABgly) significantly reduced anti-I53_dn5A pentamer antibody responses compared with immunization with unmodified I53_dn5 nanoparticle (Figure S4A). Anti-I53_dn5A pentamer antibody titers were even further reduced when 10 chains of 1 or 2 kDa PEG (I53_dn5_2C1kPEG, I53_dn5_2C2kPEG) or five unstructured polypeptides (I53_dn5_XTEN, I53_dn5_PAS, I53_dn5_ELP) masked each I53_dn5A pentamer in the nanoparticle immunogen (Figure S4A). All scaffold masking approaches significantly reduced anti-I53_dn5B trimer and anti-I53_dn5 nanoparticle IgG titers compared with those elicited by unmodified I53_dn5 particles (Figures S4B and S4C). In summary, all three masking strategies reduced antibody responses against the I53_dn5B trimer and assembled I53_dn5 nanoparticle, with PEG and unstructured polypeptides masking the I53_dn5A pentamer more efficiently than glycans.

We next assessed the immunological impact of masking the nanoparticle scaffold when the HA antigen was presented on the nanoparticle. Scaffold-masked HA-I53_dn5 nanoparticle immunogens were formed by assembling HA-I53_dn5B with I53_dn5A pentamers bearing either 10 glycans (HA-I53_dn5_Agly), 10 linear 2-kDa PEG chains (HA-I53_dn5_2C2kPEG), or five unstructured PAS polypeptides (HA-I53_dn5_PAS) (Figures 1A–1C). Based on our finding that scaffold masking reduced anti-particle responses when no viral glycoprotein antigen was displayed (Figures S4A–S4C), we hypothesized that

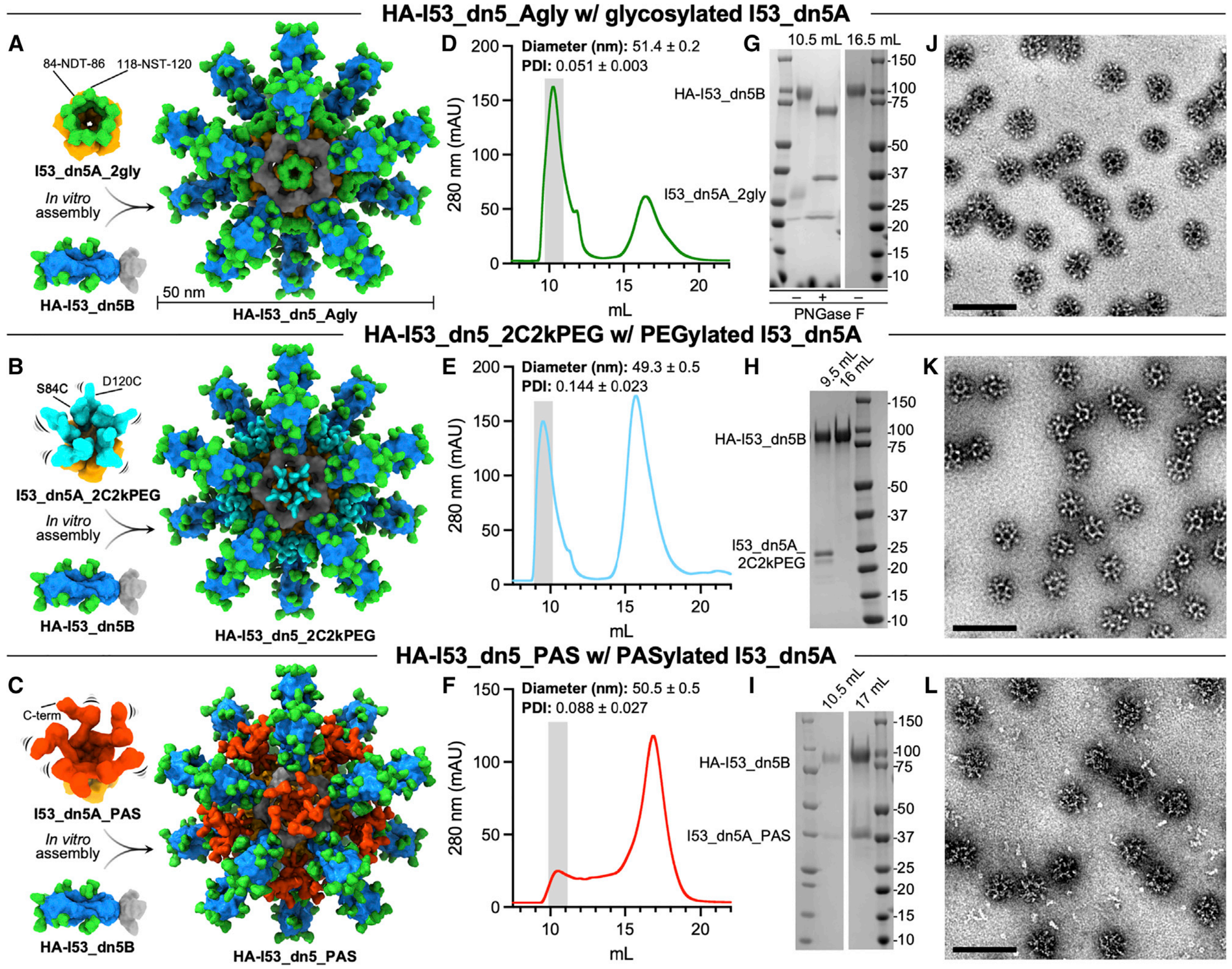


**Figure 1. Design and characterization of HA-I53_dn5 nanoparticle immunogens with a glycosylated, PEGylated, or PASylated scaffold**

(A–C) (Left) Structural models of the glycosylated pentameric I53_dn5A_2gly (I53_dn5A in orange, glycans in green at PNGS 84-NDT-86 and 118-NST-120) (A), PEGylated pentameric I53_dn5A_2C2kPEG (2-kDa PEG in teal at Cys84 and Cys120) (B), PASylated pentameric I53_dn5A_PAS (63-amino acid C-terminal "PAS" polypeptide in red) (C), and trimeric HA-I53_dn5B (HA in blue, glycans in green, and I53_dn5B in gray) components. (Right) Upon mixing *in vitro*, 20 trimeric and 12 pentameric components spontaneously assemble to form nanoparticle immunogens with icosahedral symmetry. Each nanoparticle displays 20 HA trimers and is approximately 50 nm in diameter.

(D–F) SEC purification of the HA-I53_dn5_Agly (D), HA-I53_dn5_2C2kPEG (E), and HA-I53_dn5_PAS (F) nanoparticle immunogens after *in vitro* assembly using a Superose 6 Increase 10/300 GL column. The nanoparticle immunogen elutes at the void volume of the column (gray bar). Residual, unassembled trimeric HA-I53_dn5B component elutes around 16.5 mL. The diameter and polydispersity index (PDI) of SEC-purified nanoparticles measured by DLS is reported at the top of the SEC chromatogram; DLS plots are shown in Figure S2J.

(G–I) Reducing SDS-PAGE of SEC-purified HA-I53_dn5_Agly (without and with enzymatic cleavage of glycans by ~35-kDa PNGase F) (G), HA-I53_dn5_2C2kPEG (H), and HA-I53_dn5_PAS (I) nanoparticle immunogens and residual, unassembled trimeric HA-I53_dn5B and pentameric I53_dn5A_PAS (I) components.

(J–L) Representative electron micrographs of negatively-stained HA-I53_dn5_Agly (J), HA-I53_dn5_2C2kPEG (K), and HA-I53_dn5_PAS (L) nanoparticles. Scale bars, 100 nm.

shielding surface-exposed epitopes on the nanoparticle scaffold could potentially focus and enhance the immune response to HA by reducing competition in germinal centers from scaffold-specific B cells.[76] However, following three immunizations of 0.9 μg HA (1.5 μg total protein) with AddaVax, anti-HA IgG titers were not enhanced for any of the scaffold-masked immunogens compared with the HA-I53_dn5 immunogen (Figures 2A and S4H). At the same time, when HA was present on the particle, scaffold masking had a much smaller—in some cases indiscernible—effect on reducing anti-scaffold IgG titers (Figures 2B–2D and S4I–S4K), suggesting immunodominance of HA over the underlying I53_dn5 scaffold. Similarly, for a different icosahedral nanoparticle immunogen in which half of the 20 trimers displayed prefusion RSV F antigen and

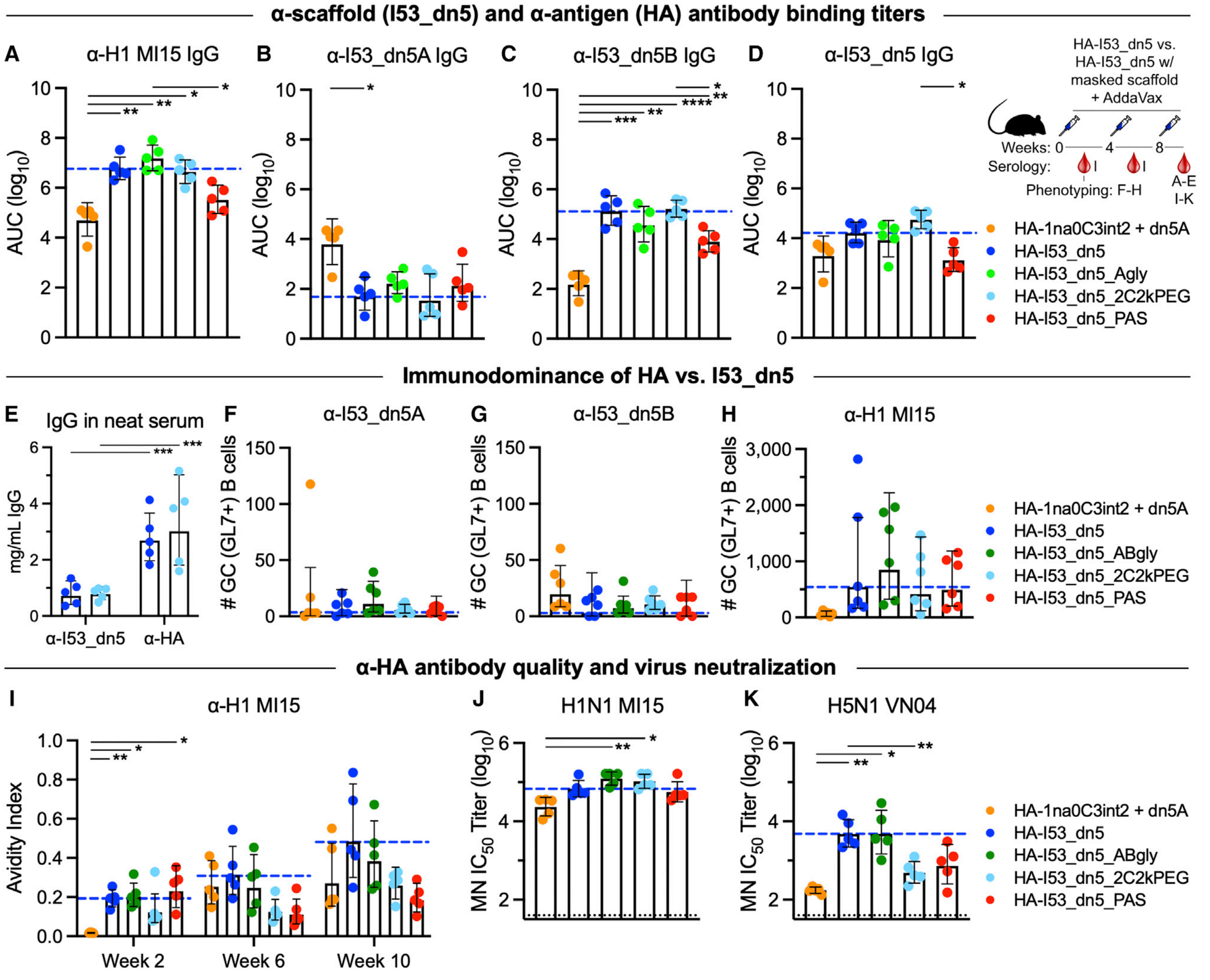


**Figure 2. Glycosylating, PEGylating, or PASylating the nanoparticle scaffold of HA-I53_dn5 immunogens does not enhance anti-HA antibody responses**

(A–D) Post-second boost (week 10) anti-H1 MI15 hemagglutinin (A), anti-I53_dn5A pentamer (B), anti-I53_dn5B trimer (C), and anti-I53_dn5 nanoparticle (D) serum IgG binding titers in BALB/c mice, measured by enzyme linked immunosorbent assay (ELISA) and plotted as the area under the curve (AUC) for each serum dilution series. Each symbol represents an individual animal, and the geometric mean AUC and the geometric mean SD from each group is indicated by the bar and error bar, respectively (N = 5 mice/group). The inset depicts the study timeline and the blood collection time point that each data panel represents.

(E) Post-second boost (week 10) anti-I53_dn5 nanoparticle and anti-H1 MI15 hemagglutinin serum IgG levels (mg/mL) elicited by HA-I53_dn5 and HA-I53_dn5_2C2kPEG nanoparticle immunogens in BALB/c mice, measured by ELISA.

(F–H) Number of I53_dn5A pentamer$^+$ (F), I53_dn5B trimer$^+$ (G), and H1 MI15 hemagglutinin$^+$ (H) lymph node GC precursors and B cells (CD38$^{+/-}$GL7$^+$) detected for each immunization group in BALB/c mice. N = 6 across two experiments for each group. (I) Post-prime (week 2), post-first boost (week 6), and post-second boost (week 10) anti-H1 MI15 hemagglutinin geometric mean Ab avidity index. The mouse immunization study was repeated twice, and representative data are shown.

(J and K) Serum microneutralization (MN) titers against vaccine-matched H1N1 (J) and heterosubtypic H5N1 (K) viruses.

The blue dashed line represents levels for the HA-I53_dn5 immunogen for comparison, and the dotted black line represents the lower limit of detection of the assay. Mouse immunization studies were repeated twice, and representative data are shown. p values between groups were determined by Brown-Forsythe and Welch one-way ANOVA test, with Dunnett's T3 multiple comparisons test. *$p < 0.05$; **$p < 0.01$; ***$p < 0.001$; ****$p < 0.0001$.

the other half bore 12 glycans per trimer (120 glycans per particle), the presence of the glycans did not dampen the anti-I53-50 scaffold IgG responses and also did not enhance anti-F responses relative to a corresponding non-glycosylated immunogen (Figures S4L–S4O). However, the presence of prefusion F on the nanoparticle immunogens significantly reduced antibody responses against the I53-50 nanoparticle compared with immunization with unmodified I53-50 (Figures S4N–S4O), again suggesting immunodominance of the displayed antigen.

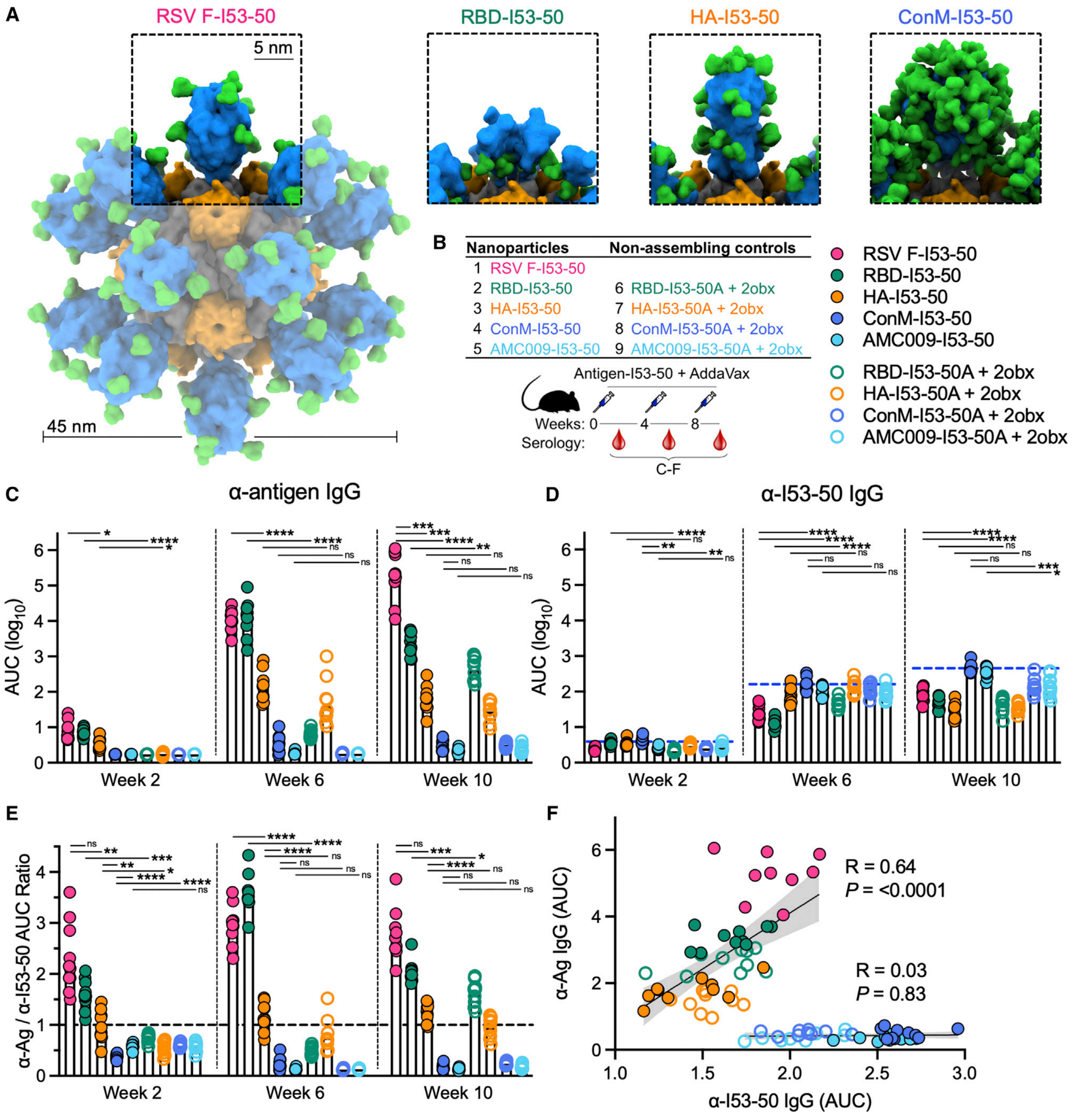


**Figure 3. Only HIV-1 Env is subdominant to the nanoparticle scaffold in a series of different nanoparticle immunogens that all use the same I53-50 scaffold**

(A) Schematic representation of the series of nanoparticle immunogens used in this study that all use the same I53-50 scaffold, highlighting the structural differences in the displayed antigen for each immunogen (antigen in blue, glycans in green, I53-50A trimeric component in gray, I53-50B pentameric component in orange).

(B) Table listing the nanoparticle and non-assembling control immunogens and schematic depicting the study timeline and blood collection time points that each data panel represents.

(C and D) Antigen-specific (C) and I53-50 scaffold-specific (D) serum IgG binding titers in BALB/c mice immunized with the color-coded immunogens listed in the table in (B), measured by ELISA and plotted as the area under the curve (AUC) for each serum dilution series. Antigen-specific IgG titers were measured by Ni-NTA-capture ELISA for more accurate comparison among immunogen groups. Each symbol represents an individual animal, and the geometric mean AUC from each group is indicated by the bar (N = 10 mice/group). The blue dashed line in (D) represents levels for the ConM-I53-50 immunogen for comparison.

(E) Ratio of the antigen-specific (C) to I53-50 scaffold-specific (D) binding antibody AUC titers. The black dashed line indicates a ratio of 1.

*(legend continued on next page)*

Interestingly, a non-assembling control immunogen in which the trimeric component lacked the computationally designed interface that drives nanoparticle assembly (HA-1na0C3int2 + I53_dn5A; [30]) elicited significantly higher anti-I53_dn5A pentamer titers (Figures 2B, S4E, and S4I) and significantly lower anti-HA (Figures 2A, S4D, and S4H) and anti-I53_dn5B (Figures 2C, S4F, and S4J) titers than HA-I53_dn5 nanoparticles. The fact that the immunogenicity of I53_dn5A was enhanced when the pentamer was physically separated from HA-I53_dn5B further suggested that in the nanoparticle context HA (1) is immunodominant over and outcompetes responses to the I53_dn5A pentamer or (2) sterically occludes BCR access to I53_dn5A. This immunodominance of HA and suppression of antibody responses to other proteins in closeness, proximity to HA is consistent with influenza neuraminidase (NA) being more immunogenic when not co-delivered with HA.[77]

To further characterize the magnitude of the response to various parts of the HA-bearing nanoparticle immunogens, we quantified antigen- and scaffold-specific IgG concentrations in neat serum as well as antigen- and scaffold-specific B cells in lymph node germinal centers (GCs). We found the amount of anti-HA IgG in undiluted serum (~3 mg/mL) was ~3-fold higher than the amount of anti-I53_dn5 IgG (~1 mg/mL) for both the HA-I53_dn5 and HA-I53_dn5_2C2kPEG immunogens (Figure 2E). In lymph nodes, the numbers of HA-specific GC ($GL7^+$) B cells were ~50-fold higher than I53_dn5A- or I53_dn5B-specific GC B cells (Figures 2F–2H and S4P). These data are consistent with our ELISA data, again indicating the displayed HA antigen is immunodominant over the underlying I53_dn5 scaffold.

We also assessed anti-HA IgG quality and binding affinity in a chaotropic ELISA that challenged serum IgG binding with 2 M NaSCN, which showed a non-significant trend of diminished antibody avidity in sera after the second and third immunizations for the non-assembling immunogen and the nanoparticle immunogens with PEG and PAS masking (Figure 2I). Instability of the HA-I53_dn5_PAS nanoparticle immunogen may be a factor here, since these nanoparticles did not fully assemble *in vitro* (Figures 1F, 1I, and 1L) and anti-HA and anti-I53_dn5 IgG titers trended toward those elicited by the non-assembling control (Figures 2A–2D). However, in an immunodepletion experiment that eliminated antibodies against the I53_dn5 nanoparticle exterior, residual antibody binding against epitopes on the interior surface of the nanoparticle or buried upon nanoparticle assembly—which could become exposed upon nanoparticle disassembly *in vivo*—were nearly completely removed from the sera of mice that received the HA-I53_dn5 and HA-I53_dn5_PAS immunogens, but not the non-assembling control immunogen (Figure S3A). This result indicates that both the HA-I53_dn5 and HA-I53_dn5_PAS immunogens are stable *in vivo* and remain intact long enough to prevent the elicitation of substantial antibody responses against epitopes on nanoparticle interior surfaces. Lastly, although microneutralization titers in immune sera against vaccine-matched virus (H1N1 MI15) were similar for all nanoparticle immunogens (Figure 2J), microneutralization titers against a heterosubtypic virus (H5/A/Vietnam/1203/04) were similar when the scaffold was masked with glycans but diminished when masked with PEG or PAS compared with non-scaffold-masked HA-I53_dn5 (Figure 2K). These data suggest that the large and structurally dynamic linear PEG and PAS polypeptides, but not branched glycans, sterically occlude the conserved antigenic site in the HA stem and/or promote particle instability/disassembly, reducing the elicitation of antibodies mediating heterosubtypic neutralization.

In summary, these three scaffold masking strategies reduced antibody responses against the I53_dn5 particle when no viral glycoprotein antigen was displayed. However, when HA and RSV F were presented on the I53_dn5 and I53-50 scaffolds, respectively, scaffold masking did not dampen anti-scaffold antibody responses and did not enhance, but in some cases diminished (e.g., PEG and PAS), antigen-specific antibody responses.

### In a series of nanoparticle immunogens that all used the same I53-50 scaffold, only HIV-1 Env was subdominant to the nanoparticle scaffold

To comparatively evaluate the immunogenicity of a range of different viral glycoprotein antigens displayed on the same protein nanoparticle scaffold and the level of anti-scaffold antibody responses elicited by each, we displayed five different viral glycoprotein antigens (prefusion RSV F, SARS-CoV-2 RBD, influenza HA, and two different native-like HIV-1 Env trimers: ConM and AMC009) separately on the two-component nanoparticle I53-50 (Figures 3A and S5). We used I53-50 as the nanoparticle scaffold for these experiments because we and others have used I53-50 to display a wide variety of antigens, including RSV F, SARS-CoV-2 RBD, and HIV-1 Env.[8,9,33] Two different native-like HIV-1 Env trimers were used because of their different immunogenicities: ConM is more immunogenic than AMC009, as the latter trimer has a denser glycan shield.[78,79] To specifically explore the role of nanoparticle formation in the relative immunogenicity of antigen and scaffold, we also prepared non-assembling control immunogens for RBD, HA, and the two Env trimers comprising a version of the I53-50B pentamer ("2obx"[80]) lacking the computationally designed interface that drives nanoparticle assembly.

Following one, two, and three immunizations with 72.4 pmol antigen (equal to 5 μg HIV-1 Env ConM and 3.0 μg I53-50 for each nanoparticle immunogen; Figure 3B), RSV F and SARS-CoV-2 RBD on I53-50 elicited the highest antigen-specific antibody titers; HA on I53-50 elicited intermediate antigen-specific titers; and both HIV-1 Env trimers (ConM and AMC009) on I53-50 elicited the lowest antigen-specific titers (Figures 3C and S6A). Thus, the antigen immunogenicity hierarchy for I53-50-scaffolded nanoparticle immunogens was RSV F > SARS-CoV-2 RBD > HA > HIV-1 Env ConM > HIV-1 Env AMC009. Comparison of the antigen-specific titers elicited by assembled

(F) Spearman's correlations between post-second boost (week 10) anti-antigen and anti-I53-50 scaffold serum IgG titers (AUC) for all immunogens on the same plot. Shaded areas represent 95% confidence intervals of the plotted linear regression line. Each symbol represents a mouse (N = 10 per immunogen).
p values between groups were determined by Brown-Forsythe and Welch one-way ANOVA test, with Dunnett's T3 multiple comparisons test. ns, non-significant; $^*p < 0.05$; $^{**}p < 0.01$; $^{***}p < 0.001$; $^{****}p < 0.0001$.

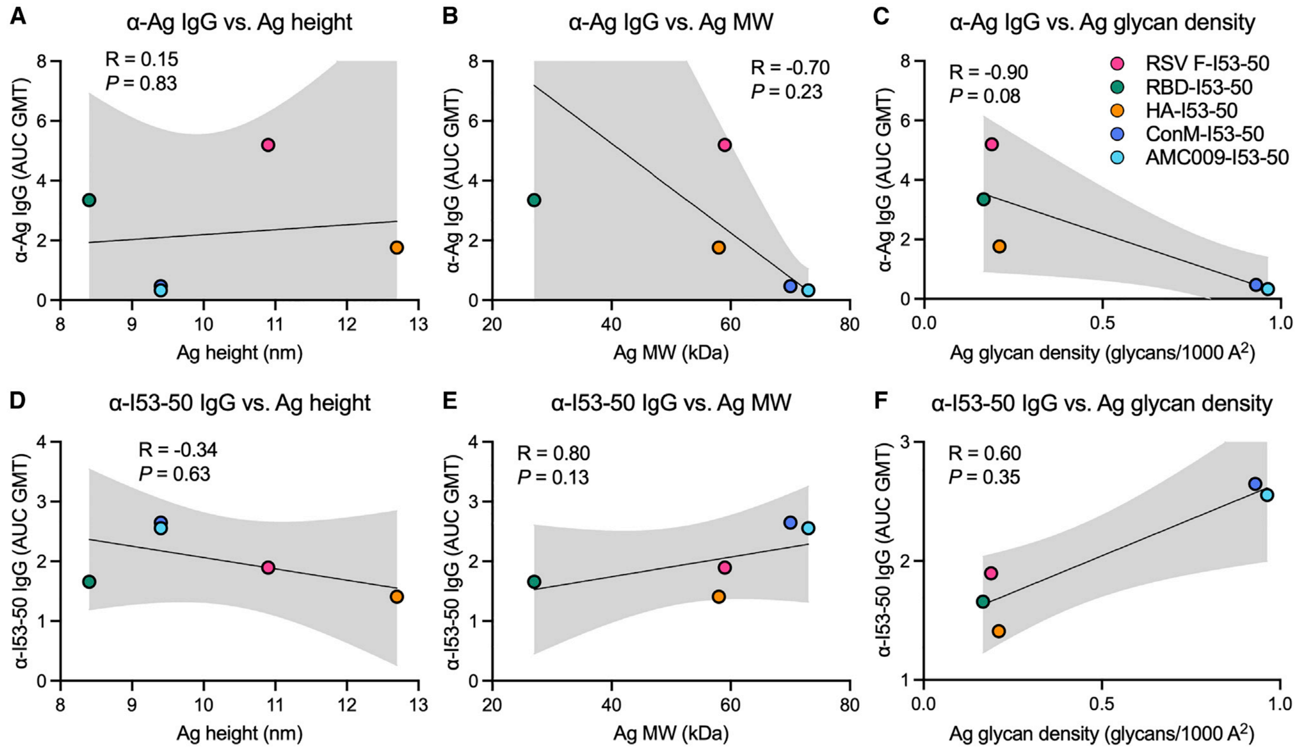


**Figure 4. Antigen- and scaffold-specific antibody responses are not correlated with antigen height, molecular weight, or glycan density**
(A–F) Spearman's correlations between post-second boost (week 10) antigen-specific (A–C) or I53-50 scaffold-specific (D–F) serum IgG levels and antigen height (A and D), molecular weight (B and E), and glycan density (C and F). Shaded areas represent 95% confidence intervals of the plotted linear regression line. Each symbol represents the antigen-specific or I53-50-specific serum IgG geometric mean AUC (AUC GMT) for the indicated nanoparticle immunogen (N = 10 mice per immunogen).

versus non-assembled nanoparticles showed that significant improvement in antigen-specific titers was only observed for RBD-I53-50, but not for HA-I53-50, ConM-I53-50, or AMC009-I53-50 (Figure 3C). Antigen-specific titers from the non-assembling controls increased after booster immunizations for the RBD and HA immunogens, but the antigen-specific titers for the HIV-1 Env non-assembling controls remained near baseline levels (Figures 3C and S6A). Conversely, anti-I53-50 titers for the ConM-I53-50 group were the highest among all immunogens at all time points (Figure 3D). This suggests that despite its relatively large size, the poorly immunogenic HIV-1 Env may impart less antigenic competition with the nanoparticle scaffold than the other more immunogenic antigens. Alternatively, more efficient trafficking of HIV-1 Env immunogens to lymph nodes due to the high oligomannose glycan density on Env[25,81,82] may be another mechanism by which the immunogenicity of the underlying I53-50 scaffold is increased. Furthermore, the ratio of antigen-specific over anti-I53-50 titers for the HIV-1 Env groups was consistently less than 1 at all time points, while the other nanoparticle immunogen groups exhibited ratios equal to or greater than 1 (Figures 3E and S6A). Therefore, for this series of nanoparticle immunogens, only HIV-1 Env was immunosubdominant to the nanoparticle scaffold.

The antigen-specific and scaffold-specific antibody titers did not correlate with the physical size of the antigen, measured by either antigen height or molecular weight (Figures 4A, 4B, 4D, and 4E), although we note that the sample size (N = 5) is small for meaningful correlation analysis. Furthermore, for the most part there are not substantial differences between the anti-scaffold responses in corresponding assembling and non-assembling groups (the exception being the RBD immunogens at week 6) (Figure 3D). These data suggest that the anti-scaffold response is not primarily determined by sterics/physical access to the scaffold surfaces. However, we cannot rule out that particle disassembly *in vivo* could be a factor here. Another potential factor driving the antigen immunogenicity hierarchy could be glycan density on the antigens; however, this failed to correlate with antigen-specific or scaffold-specific antibody titers (Figures 4C and 4F). Moreover, each immunogen failed to show a negative correlation between antigen-specific and scaffold-specific responses (Figure S6B), suggesting that anti-scaffold responses do not interfere with antigen-specific responses for any of these immunogens. Instead, when all immunogens (except Env immunogens) were grouped together, antigen-specific and scaffold-specific responses were highly positively correlated ($p < 0.0001$), while the Env immunogens grouped together exhibited no correlation ($p = 0.83$) (Figure 3F). Taken together, these data indicate that within a nanoparticle immunogen there is not zero-sum antigen competition between antigen-specific and scaffold-specific antibody responses, since

CellPress
OPEN ACCESS

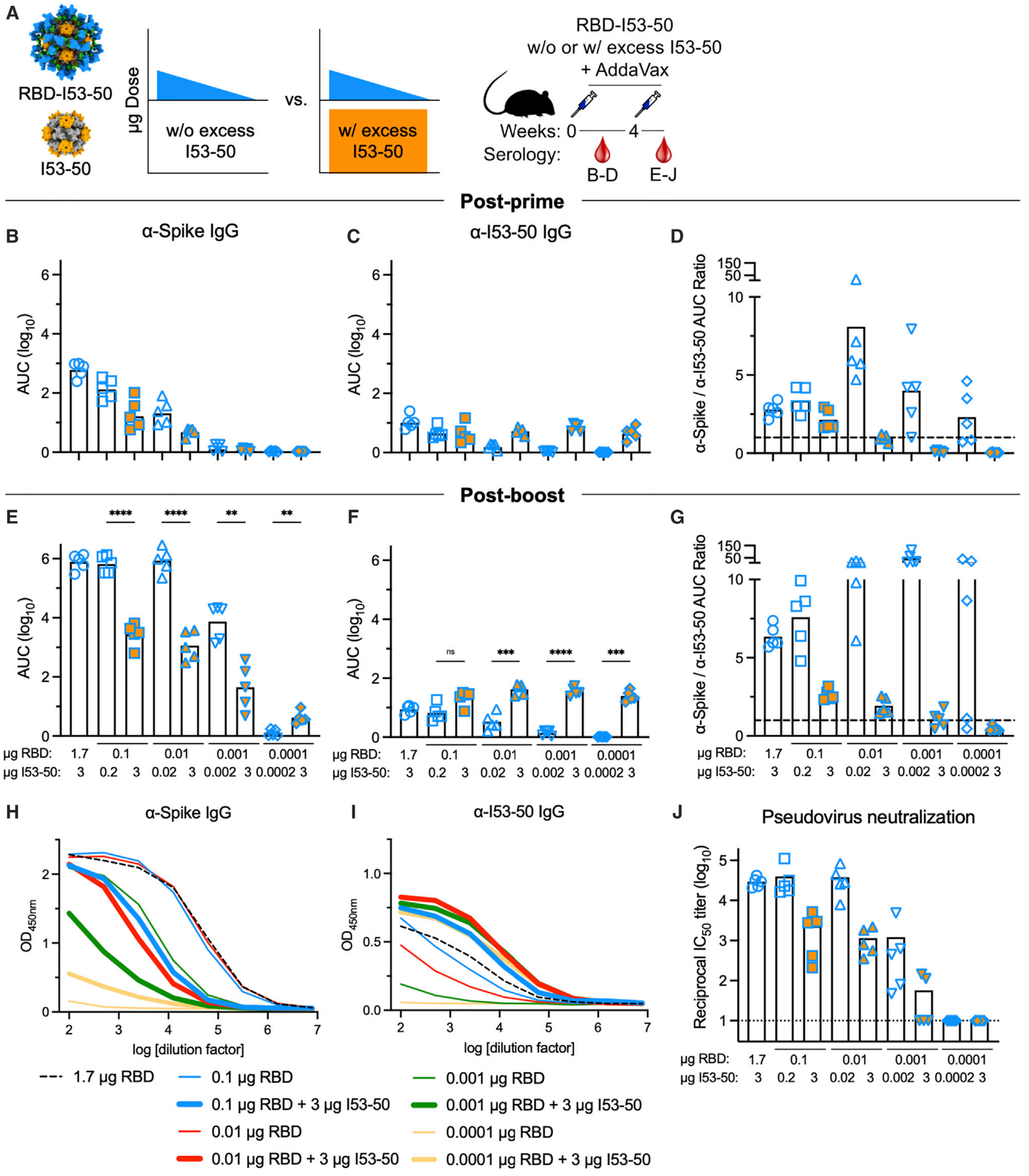


**Figure 5. Scaffold-specific antibody responses can competitively inhibit antigen-specific responses when scaffold is provided in excess**

(A) (Left) Schematic representation of the scaffold competition experimental design where mice were immunized with either varying doses of RBD-I53-50 nanoparticle immunogens (blue triangle) or varying doses of RBD-I53-50 nanoparticle immunogens co-delivered with excess I53-50 scaffold (blue triangle + orange rectangle). (Right) Schematic depicting the study timeline and blood collection time points that each data panel represents.

(B–G) Post-prime (week 2) (B–D) and post-boost (week 6) (E–G) anti-SARS-CoV-2 spike (B and E) and anti-I53-50 scaffold (C and F) serum IgG binding titers in BALB/c mice immunized with the protein doses indicated at the bottom of (E)–(G), measured by ELISA and plotted as the area under the curve (AUC) for each serum dilution series. Each symbol represents an individual animal, and the geometric mean AUC from each group is indicated by the bar (N = 5 mice/group).

*(legend continued on next page)*

an increase in one does not result in a proportional decrease in the other. Instead, there is a significant positive correlation between antigen-specific and anti-scaffold responses across this set of non-Env antigens (Figure 3F).

## Competition from excess I53-50 nanoparticle scaffold suppresses antigen-specific antibody responses

To better understand the antigen vs. scaffold immunodominance hierarchies observed above and the potential role of antigenic competition between the displayed antigen and nanoparticle scaffold, we compared antigen-specific and scaffold-specific antibody responses elicited by a 10,000-fold dose range of RBD-I53-50 co-administered with a constant dose of excess I53-50 protein. Although we were unable to observe clear evidence of antigenic competition in the experiments presented above, we hypothesized that the addition of excess I53-50—to artificially inflate the scaffold to antigen ratio—might allow us to observe suppression of antigen-specific antibody responses due to antigenic competition, similar to how excess carrier protein outcompeted and suppressed hapten-specific antibody responses.[83] We used the RBD nanoparticle immunogen in this experiment based on our finding above that the RBD is strongly immunodominant to the I53-50 scaffold (Figures 3C–3E).

We immunized mice with RBD-I53-50 comprising 1.7, 0.1, 0.01, 0.001, or 0.0001 μg RBD with or without co-administration of excess I53-50 to a total of 3 μg of the nanoparticle scaffold (Figure 5A). After a single immunization, we observed a typical dose-response effect in both the RBD-specific and anti-scaffold antibody responses, with loss of detectable antibodies at 0.0001 μg RBD and 0.002 μg I53-50, respectively (Figures 5B and 5C). The effect of co-administering excess I53-50 was already apparent post-prime. RBD-specific antibodies were 8.1- and 4.4-fold lower at the 0.1 and 0.01 μg RBD doses relative to the conditions without excess scaffold, respectively (Figure 5B), while anti-scaffold responses, in the presence of excess I53-50, were roughly constant over the entire dose range (Figure 5C). The average post-prime antigen-specific to scaffold-specific area under the curve (AUC) ratio was greater than 1 for all groups post-prime except for the 0.01, 0.001, and 0.0001 μg RBD doses with excess I53-50 (Figure 5D). Post-boost, these trends were amplified, with the exception that there was no diminution in the RBD-specific antibody responses when decreasing the RBD dose from 1.7 to 0.01 μg, although further decreases in dose led to lower anti-RBD responses (Figures 5E and 5H). Suppression of the RBD-specific antibodies by excess I53-50 was more pronounced post-boost, with decreases of 113-, 266-, and 147-fold at doses of 0.1, 0.01, and 0.001 μg RBD relative to the conditions without excess scaffold, respectively (Figures 5E and 5H). There was also a trend of reduced post-boost pseudovirus neutralization ($IC_{50}$) in the presence of excess I53-50, with decreases of 23-, 32-, and 13-fold at doses of 0.1, 0.01, and 0.001 μg RBD, respectively (Figure 5J). There was still a clear dose-response effect in the anti-scaffold responses in the absence of co-administered I53-50 (Figures 5F and 5I). Interestingly, the anti-scaffold responses with co-administered I53-50 trended higher than the 1.7 μg dose of RBD-I53-50, despite containing the same total amount of I53-50 scaffold (Figures 5F and 5I). The average post-boost antigen-specific to scaffold-specific AUC ratio was less than 1 for only the 0.001 and 0.0001 μg RBD doses with excess I53-50, whereas this AUC ratio progressively increased for 1.7, 0.1, 0.01, and 0.001 μg RBD when no excess I53-50 was present (Figure 5G). We also tried a similar competition experiment with ConM (but with a smaller dose range), but the anti-ConM antibody responses were so weak that no suppression of anti-ConM titers was detected when excess I53-50 was co-delivered (Figure S7). In addition, we tested if excess heterologous nanoparticle scaffold suppressed antigen-specific antibody responses by immunizing mice with RBD-I53-50 in the presence of excess I53_dn5 nanoparticles. Interestingly, we found that excess heterologous I53_dn5 scaffold did not suppress RBD-specific antibodies (Figures S8F and S8I), which is consistent with a report that showed that excess heterologous carrier protein did not compete with and suppress hapten-specific antibody responses.[83] Taken together, these data confirm that in the context of protein nanoparticle immunogens that display viral glycoprotein antigens, excess homologous nanoparticle scaffold, but not heterologous nanoparticle scaffold, can compete with and suppress antigen-specific binding and pseudovirus neutralizing antibody responses.

## DISCUSSION

Here, we have evaluated the role of anti-scaffold antibody responses to protein nanoparticle immunogens and their impact on antigen-specific antibody responses. Specifically, we addressed the following questions. How do the magnitudes of scaffold-specific and antigen-specific antibody responses compare? Does physically masking the nanoparticle scaffold enhance antigen-specific antibody responses? How does antigen immunodominance alter anti-scaffold responses? Lastly, can anti-scaffold responses compete with antigen-specific responses? The data reported here suggest that, despite antigenic competition between the nanoparticle scaffold and displayed

(D and G) Ratio of the post-prime (week 2) (D) and post-boost (week 6) (G) spike-specific to I53-50 scaffold-specific binding antibody titers (AUC). The black dashed line indicates a ratio of 1.

(H and I) Post-boost (week 6) spike-specific (H) and I53-50 scaffold-specific (I) serum IgG ELISA curves in BALB/c mice. Each line represents the geometric mean absorbance at 450 nm (N = 5 mice/group).

(J) Post-boost serum pseudovirus neutralization titers using a vesicular stomatitis virus (VSV) pseudotyped with the Wuhan-Hu-1 S glycoprotein harboring the D614G substitution (G614) and VeroE6 cells stably expressing TMPRSS2.[84] Each symbol represents the reciprocal half-maximal inhibitory dilution ($IC_{50}$) of an individual animal. GMT for each group is indicated by a bar (N = 5 mice/group). Representative data from duplicate measurements made with distinct batches of pseudoviruses are shown. The dotted horizontal line represents the lower limit of detection of the assay. Raw data curves are shown in Figure S7E.

p values between groups were determined by Brown-Forsythe and Welch one-way ANOVA test, with Dunnett's T3 multiple comparisons test. *$p < 0.05$; **$p < 0.01$; ***$p < 0.001$; ****$p < 0.0001$.

antigen when the former is provided in excess, scaffold masking has limited utility for enhancing antibody responses against strongly immunogenic antigens, a finding that has implications for current and future nanoparticle vaccine design.

To better understand the role of anti-scaffold immune responses, we masked the underlying I53_dn5 nanoparticle in the HA-I53_dn5 immunogen using three different approaches: glycosylation, PEGylation, and PASylation. All three approaches successfully yielded nanoparticle immunogens co-displaying a large glycoprotein antigen and the masking moieties, showcasing the robustness and versatility of computationally designed two-component nanoparticles as a multivalent display platform. However, there are limits to what can be displayed on the nanoparticle exterior: the efficiency of *in vitro* assembly was substantially reduced for the PASylated particle. We then examined how shielding the scaffold impacted anti-HA antibody responses. Overall, scaffold masking did not increase anti-HA antibody titers and in some instances (i.e., PEGylation and PASylation) appeared to occlude cross-reactive epitopes in the HA stem. The partial disassembly of HA-I53_dn5_PAS nanoparticles due to their instability may also have contributed to the reduced HA stem responses. The failure of scaffold masking to enhance antigen-specific antibody titers is consistent with an analogous study in the literature, which showed no increases in anti-Env or anti-HA antibody titers when the genetically fused GCN4-based isoleucine zipper trimerization domain was masked with glycans.[46] It is also consistent with several studies using glycan masking within antigens (rather than on heterologous domains fused to antigens) to alter the epitope specificities of polyclonal vaccine-elicited antibodies, which generally indicate that this approach yields a higher proportion, but not a higher absolute magnitude, of antibodies against unmasked epitopes.[47–49] It is likely that the lack of improved anti-HA titers is due to the immunodominance of HA[85] over the scaffold (i.e., anti-scaffold responses do not strongly interfere with anti-HA responses). This notion is consistent with the positive correlation previously observed between antigen-specific and scaffold-specific antibody titers in NHPs immunized with the immunodominant SARS-CoV-2 RBD displayed on the I53-50 scaffold, which demonstrated that pre-existing and increased anti-scaffold antibody responses did not interfere with antigen-specific responses during boosting.[36] Overall, these observations suggest that masking the scaffolds of other nanoparticle immunogens that display an immunodominant antigen may be ineffective at improving the magnitude of the antigen-specific antibody response, although we note that there may be other beneficial effects that could derive from scaffold masking, particularly with glycans.[25,82]

Several protein nanoparticle immunogens have been reported to elicit robust levels of antibodies against both the nanoparticle scaffolds and the displayed antigen (e.g., RTS,S,[39] HA-ferritin,[5] DS-Cav1-I53-50,[8] and SARS-CoV-2-RBD-I53-50[9]). Interestingly, a newer version of the recombinant malaria vaccine RTS,S known as R21, which displays the circumsporozoite protein (CSP) antigen at full valency in a highly dense array on the hepatitis B surface antigen scaffold, elicited enhanced antigen-specific antibody titers and reduced anti-scaffold responses compared with RTS,S,[86] and is a more efficacious vaccine.[87] Furthermore, anti-SARS-CoV-2 RBD antibody titers were enhanced when 60 copies of the RBD were densely displayed on a β-annulus peptide scaffold that elicited low anti-scaffold responses compared with RBD-ferritin and RBD-lumazine synthase immunogens.[88] However, it is difficult to elucidate to what extent, if any, the reduced anti-scaffold titers contributed to the enhanced antigen-specific titers compared with the increase in antigen density, a known correlate of immunogenicity in nanoparticle immunogens.[8,89] Here, we confirmed the immunodominance of prefusion RSV F, SARS-CoV-2 RBD, and influenza HA on the I53-50 scaffold, and we showed the subdominance of two variants of HIV-1 Env (ConM and AMC009) on I53-50. This study design allowed for direct comparison of the antigen-specific and scaffold-specific immune responses, and we showed that anti-scaffold antibody responses are not negatively correlated with antigen-specific responses for this set of immunogens. The subdominance of HIV-1 Env, which is consistent with studies in rabbits immunized with Env-I53-50 and Env-ferritin immunogens,[33,34] suggests that, in contrast to the other immunodominant antigens, masking the underlying scaffold may enhance anti-Env antibody responses, a question that could be explored in future work. However, caution is warranted since a previous study showed that glycosylation of an immunogenic trimerization domain fused to Env did not enhance anti-Env antibody titers.[46] Using different masking approaches than those employed here and more completely shielding exposed scaffold surfaces may be more effective at antigen-specific immunofocusing.

Antigenic competition determines immunodominance patterns for complex immunogens.[85,90] Subdominant antibody responses arise when BCR access is occluded and/or low frequency B cells or those with low-affinity BCRs cannot compete for expansion within GCs.[91–93] Here, we showed that co-delivery of excess I53-50 scaffold with RBD-I53-50 immunogens suppressed immunodominant antigen-specific antibody responses, but co-delivery of excess heterologous I53_dn5 scaffold with RBD-I53-50 immunogens did not suppress antigen-specific antibody responses. These data suggest that immunodominant antibody responses (e.g., RBD-specific) are suppressed when subdominant (e.g., scaffold) epitopes are increased in abundance, are no longer physically linked to immunodominant epitopes, and/or are more accessible to BCRs. Co-delivered excess scaffold could also outcompete antigen-specific B cells for the limited T cell help available for GC reactions, and consequently suppress antigen-specific responses.[83] Therefore, for nanoparticle immunogens in which scaffold- and antigen-specific responses are on a roughly equal footing, anti-scaffold responses may impede antigen-specific responses, similar to the anti-ferritin responses that were recently reported to detract from Env-specific neutralization titers for an Env-ferritin immunogen.[34] Our data also imply that protein nanoparticle immunogens with reduced antigen valency, in which some of the potential antigen-bearing sites are left vacant, could suffer from anti-scaffold responses suppressing antigen-specific responses. We note that RBD-I53-50 particles with 50% RBD valency elicited in mice similar anti-spike antibody titers but slightly higher anti-scaffold titers than the 100% RBD valency groups,[9] potentially due to the 2-fold higher scaffold dose and increased

accessibility of the scaffold to BCRs. Similar results were observed with a series of I53-50 nanoparticles displaying prefusion RSV F at 33%, 67%, and 100% valency.[8] Overall, we have shown that protein nanoparticle scaffolds are a potential source of antigenic competition, which is an important consideration when designing complex immunogens.

In summary, our results inform the design of protein nanoparticle immunogens. We show that while the nanoparticle scaffold can indeed compete with antigen-specific immune responses under certain conditions, physically masking a scaffold that multivalently presents immunodominant antigen holds limited utility for enhancing antigen-specific antibody responses. Improving immunogenicity through antigen design, modulating epitope focusing through antigen presentation geometry and co-display of multiple antigenic variants, and enhancing cell-mediated immunity by engineering improved vaccine trafficking and uptake are likely more fruitful approaches for further enhancing the performance of protein nanoparticle vaccines.

### Limitations of the study

This study has several limitations that can be addressed in future work. First, we did not attempt to mask the entire scaffold surface. The improved antigen-specific responses obtained from highly dense display of *Plasmodium falciparum* CSP in R21 particles[86] and SARS-CoV-2 RBD in β-annulus particles[88] that nearly completely shields the underlying scaffolds suggest that complete scaffold masking may be beneficial. Another important question for future studies is whether scaffold masking may be more beneficial when displaying a weakly immunogenic antigen that is subdominant to the scaffold, such as HIV-1 Env.[94,95] Furthermore, our experiments involving Env-I53-50 immunogens indicate that future studies would benefit from using an animal model such as rabbits or NHPs that have longer and more human-like HCDR3s (~15 amino acids in length) to better engage Env epitopes than mice, which have shorter HCDR3s (~11–12 amino acids in length) that restrict humoral responses to Env.[96,97] Moreover, the same antigen immunogenicity and antigen vs. scaffold immunodominance hierarchies we observed here may not be recapitulated in animals that have different immune repertoires than BALB/c mice.

## STAR★METHODS

Detailed methods are provided in the online version of this paper and include the following:

- KEY RESOURCES TABLE
- RESOURCE AVAILABILITY
  - Lead contact
  - Materials availability
  - Data and code availability
- EXPERIMENTAL MODEL AND SUBJECT DETAILS
  - Cell lines
  - Mice
- METHOD DETAILS
  - Computational design of glycosylated proteins
  - Gene synthesis and vector construction
  - Microbial protein expression and purification
  - Mammalian protein expression and purification
  - PEG-maleimide to HS-protein coupling
  - *In vitro* nanoparticle assembly and purification
  - Negative-stain electron microscopy
  - Antigenic characterization
  - Dynamic light scattering
  - Endotoxin measurements
  - Mouse immunizations and sera collection
  - Serum antibody ELISA
  - Serum antibody avidity/chaotropic ELISA
  - Ni-NTA-capture ELISA
  - Sera immunodepletion
  - Reporter-based microneutralization assay
  - Pseudovirus production
  - Pseudovirus neutralization
  - Tetramer production
  - Mouse immunization, cell enrichment, and flow cytometry
  - Statistical analysis

## SUPPLEMENTAL INFORMATION

Supplemental information can be found online at https://doi.org/10.1016/j.xcrm.2022.100780.

## ACKNOWLEDGMENTS

The authors gratefully acknowledge Jared Adolf-Bryfogle and Jason Labonte for helpful discussions on modeling glycans with Rosetta, Liz Soberg for assistance with animal studies, and Ratika Krishnamurty for program management. This study was supported by the Bill & Melinda Gates Foundation (OPP1156262 and INV-002022), the Defense Threat Reduction Agency (HDTRA1-18-1-0001 to N.P.K.) a generous gift from the Audacious Project, the National Institute of Allergy and Infectious Diseases (DP1AI158186 and HHSN272201700059C to D.V.), a Pew Biomedical Scholars Award (D.V.), an Investigators in the Pathogenesis of Infectious Disease Award from the Burroughs Wellcome Fund (D.V.), Fast Grants (D.V.), and the National Science Foundation Graduate Research Fellowship Program under Grant No. DGE-1762114 (L.S.). A.A. is supported by amfAR Mathilde Krim Fellowship in Biomedical Research #110182-69-RKVA. D.V. is an Investigator of the Howard Hughes Medical Institute. R.W.S. is a recipient of a Vici fellowship from the Netherlands Organization for Scientific Research (NWO). We thank Hideki Tani (University of Toyama) for providing the reagents necessary for preparing VSV pseudotyped viruses.

## AUTHOR CONTRIBUTIONS

Conceptualization: J.C.K. and N.P.K.; design: J.C.K., P.J.M.B., A.A., A.B.W., R.W.S., and N.P.K.; formal analysis: J.C.K., L.S., S.B.B., A.C.W., K.R.S., and N.P.K.; resources: J.C.K., M.N.P., L.S., M.B., S.B.B., A.C.W., K.R.S., S.C., M.M., D.P., M.A., C.S., M.J., A.B., D.E., R.R., B.F., S.W., M.M., K.S., P.J.M.B., M.K., D.V., M.P., R.W.S., and N.P.K.; writing (original draft): J.C.K. and N.P.K.; writing (review & editing): all authors; visualization: J.C.K. and N.P.K.; supervision: D.V., A.B.W., M.K., M.P., R.W.S., and N.P.K.; funding acquisition: D.V., M.K., M.P., R.W.S., and N.P.K.

## DECLARATION OF INTERESTS

J.C.K. and N.P.K. are named as inventors on a patent application filed by the University of Washington based on the glycosylated protein nanoparticles presented in this paper. N.P.K. is a co-founder, shareholder, paid consultant, and chair of the scientific advisory board of Icosavax, Inc. The King lab has received unrelated sponsored research agreements from Pfizer and GSK.

CellPress
OPEN ACCESS

The Veesler lab has received an unrelated sponsored research agreement from Vir Biotechnology.

## INCLUSION AND DIVERSITY

We support inclusive, diverse, and equitable conduct of research.

Received: February 28, 2022
Revised: May 27, 2022
Accepted: September 22, 2022
Published: September 26, 2022

## REFERENCES

1. Adolf-Bryfogle, J., Labonte, J.W., Kraft, J.C., Shapovalov, M., Raemisch, S., Lütteke, T., DiMaio, F., Bahl, C.D., Pallesen, J., King, N.P., et al. (2021). Growing glycans in rosetta: accurate de novo glycan modeling, density fitting, and rational sequon design. Preprint at bioRxiv. https://doi.org/10.1101/2021.09.27.462000.
2. Weissman, D., Ni, H., Scales, D., Dude, A., Capodici, J., McGibney, K., Abdool, A., Isaacs, S.N., Cannon, G., and Karikó, K. (2000). HIV gag mRNA transfection of dendritic cells (DC) delivers encoded antigen to MHC class I and II molecules, causes DC maturation, and induces a potent human in vitro primary immune response. J. Immunol. *165*, 4710–4717.
3. Pardi, N., Hogan, M.J., Porter, F.W., and Weissman, D. (2018). mRNA vaccines - a new era in vaccinology. Nat. Rev. Drug Discov. *17*, 261–279.
4. Gordon, D.M., McGovern, T.W., Krzych, U., Cohen, J.C., Schneider, I., LaChance, R., Heppner, D.G., Yuan, G., Hollingdale, M., and Slaoui, M. (1995). Safety, immunogenicity, and efficacy of a recombinantly produced Plasmodium falciparum circumsporozoite protein-hepatitis B surface antigen subunit vaccine. J. Infect. Dis. *171*, 1576–1585.
5. Kanekiyo, M., Wei, C.-J., Yassine, H.M., McTamney, P.M., Boyington, J.C., Whittle, J.R.R., Rao, S.S., Kong, W.-P., Wang, L., and Nabel, G.J. (2013). Self-assembling influenza nanoparticle vaccines elicit broadly neutralizing H1N1 antibodies. Nature *499*, 102–106.
6. Jardine, J.G., Ota, T., Sok, D., Pauthner, M., Kulp, D.W., Kalyuzhniy, O., Skog, P.D., Thinnes, T.C., Bhullar, D., Briney, B., et al. (2015). HIV-1 VACCINES. Priming a broadly neutralizing antibody response to HIV-1 using a germline-targeting immunogen. Science *349*, 156–161.
7. López-Sagaseta, J., Malito, E., Rappuoli, R., and Bottomley, M.J. (2016). Self-assembling protein nanoparticles in the design of vaccines. Comput. Struct. Biotechnol. J. *14*, 58–68.
8. Marcandalli, J., Fiala, B., Ols, S., Perotti, M., de van der Schueren, W., Snijder, J., Hodge, E., Benhaim, M., Ravichandran, R., Carter, L., et al. (2019). Induction of potent neutralizing antibody responses by a designed protein nanoparticle vaccine for respiratory syncytial virus. Cell *176*, 1420–1431.e17.
9. Walls, A.C., Fiala, B., Schäfer, A., Wrenn, S., Pham, M.N., Murphy, M., Tse, L.V., Shehata, L., O'Connor, M.A., Chen, C., et al. (2020). Elicitation of potent neutralizing antibody responses by designed protein nanoparticle vaccines for SARS-CoV-2. Cell *183*, 1367–1382.e17.
10. Boyoglu-Barnum, S., Ellis, D., Gillespie, R.A., Hutchinson, G.B., Park, Y.-J., Moin, S.M., Acton, O.J., Ravichandran, R., Murphy, M., Pettie, D., et al. (2021). Quadrivalent influenza nanoparticle vaccines induce broad protection. Nature *592*, 623–628.
11. Walls, A.C., Miranda, M.C., Schäfer, A., Pham, M.N., Greaney, A., Arunachalam, P.S., Navarro, M.-J., Tortorici, M.A., Rogers, K., O'Connor, M.A., et al. (2021). Elicitation of broadly protective sarbecovirus immunity by receptor-binding domain nanoparticle vaccines. Cell *184*, 5432–5447.e16.
12. Burton, D.R. (2002). Antibodies, viruses and vaccines. Nat. Rev. Immunol. *2*, 706–713.
13. Rappuoli, R., Bottomley, M.J., D'Oro, U., Finco, O., and De Gregorio, E. (2016). Reverse vaccinology 2.0: human immunology instructs vaccine antigen design. J. Exp. Med. *213*, 469–481.
14. Sanders, R.W., Vesanen, M., Schuelke, N., Master, A., Schiffner, L., Kalyanaraman, R., Paluch, M., Berkhout, B., Maddon, P.J., Olson, W.C., et al. (2002). Stabilization of the soluble, cleaved, trimeric form of the envelope glycoprotein complex of human immunodeficiency virus type 1. J. Virol. *76*, 8875–8889.
15. Wu, X., Yang, Z.-Y., Li, Y., Hogerkorp, C.-M., Schief, W.R., Seaman, M.S., Zhou, T., Schmidt, S.D., Wu, L., Xu, L., et al. (2010). Rational design of envelope identifies broadly neutralizing human monoclonal antibodies to HIV-1. Science *329*, 856–861.
16. Jardine, J., Julien, J.-P., Menis, S., Ota, T., Kalyuzhniy, O., McGuire, A., Sok, D., Huang, P.-S., MacPherson, S., Jones, M., et al. (2013). Rational HIV immunogen design to target specific germline B cell receptors. Science *340*, 711–716.
17. McLellan, J.S., Chen, M., Joyce, M.G., Sastry, M., Stewart-Jones, G.B.E., Yang, Y., Zhang, B., Chen, L., Srivatsan, S., Zheng, A., et al. (2013). Structure-based design of a fusion glycoprotein vaccine for respiratory syncytial virus. Science *342*, 592–598.
18. Yassine, H.M., Boyington, J.C., McTamney, P.M., Wei, C.-J., Kanekiyo, M., Kong, W.-P., Gallagher, J.R., Wang, L., Zhang, Y., Joyce, M.G., et al. (2015). Hemagglutinin-stem nanoparticles generate heterosubtypic influenza protection. Nat. Med. *21*, 1065–1070.
19. Pallesen, J., Wang, N., Corbett, K.S., Wrapp, D., Kirchdoerfer, R.N., Turner, H.L., Cottrell, C.A., Becker, M.M., Wang, L., Shi, W., et al. (2017). Immunogenicity and structures of a rationally designed prefusion MERS-CoV spike antigen. Proc. Natl. Acad. Sci. USA *114*, E7348–E7357.
20. Hsieh, C.-L., Goldsmith, J.A., Schaub, J.M., DiVenere, A.M., Kuo, H.-C., Javanmardi, K., Le, K.C., Wrapp, D., Lee, A.G., Liu, Y., et al. (2020). Structure-based design of prefusion-stabilized SARS-CoV-2 spikes. Science *369*, 1501–1505.
21. Ellis, D., Brunette, N., Crawford, K.H.D., Walls, A.C., Pham, M.N., Chen, C., Herpoldt, K.-L., Fiala, B., Murphy, M., Pettie, D., et al. (2021). Stabilization of the SARS-CoV-2 spike receptor-binding domain using deep mutational scanning and structure-based design. Front. Immunol. *12*, 710263.
22. Polack, F.P., Thomas, S.J., Kitchin, N., Absalon, J., Gurtman, A., Lockhart, S., Perez, J.L., Pérez Marc, G., Moreira, E.D., Zerbini, C., et al. (2020). Safety and efficacy of the BNT162b2 mRNA covid-19 vaccine. N. Engl. J. Med. *383*, 2603–2615.
23. Keech, C., Albert, G., Cho, I., Robertson, A., Reed, P., Neal, S., Plested, J.S., Zhu, M., Cloney-Clark, S., Zhou, H., et al. (2020). Phase 1-2 trial of a SARS-CoV-2 recombinant spike protein nanoparticle vaccine. N. Engl. J. Med. *383*, 2320–2332.
24. Baden, L.R., El Sahly, H.M., Essink, B., Kotloff, K., Frey, S., Novak, R., Diemert, D., Spector, S.A., Rouphael, N., Creech, C.B., et al. (2021). Efficacy and safety of the mRNA-1273 SARS-CoV-2 vaccine. N. Engl. J. Med. *384*, 403–416.
25. Tokatlian, T., Read, B.J., Jones, C.A., Kulp, D.W., Menis, S., Chang, J.Y.H., Steichen, J.M., Kumari, S., Allen, J.D., Dane, E.L., et al. (2019). Innate immune recognition of glycans targets HIV nanoparticle immunogens to germinal centers. Science *363*, 649–654.
26. Kato, Y., Abbott, R.K., Freeman, B.L., Haupt, S., Groschel, B., Silva, M., Menis, S., Irvine, D.J., Schief, W.R., and Crotty, S. (2020). Multifaceted effects of antigen valency on B cell response composition and differentiation in vivo. Immunity *53*, 548–563.e8.
27. King, N.P., Bale, J.B., Sheffler, W., McNamara, D.E., Gonen, S., Gonen, T., Yeates, T.O., and Baker, D. (2014). Accurate design of co-assembling multi-component protein nanomaterials. Nature *510*, 103–108.
28. Bale, J.B., Park, R.U., Liu, Y., Gonen, S., Gonen, T., Cascio, D., King, N.P., Yeates, T.O., and Baker, D. (2015). Structure of a designed

tetrahedral protein assembly variant engineered to have improved soluble expression. Protein Sci. *24*, 1695–1701.

29. Bale, J.B., Gonen, S., Liu, Y., Sheffler, W., Ellis, D., Thomas, C., Cascio, D., Yeates, T.O., Gonen, T., King, N.P., and Baker, D. (2016). Accurate design of megadalton-scale two-component icosahedral protein complexes. Science *353*, 389–394.
30. Ueda, G., Antanasijevic, A., Fallas, J.A., Sheffler, W., Copps, J., Ellis, D., Hutchinson, G.B., Moyer, A., Yasmeen, A., Tsybovsky, Y., et al. (2020). Tailored design of protein nanoparticle scaffolds for multivalent presentation of viral glycoprotein antigens. Elife *9*, e57659.
31. Brouwer, P.J.M., Brinkkemper, M., Maisonnasse, P., Dereuddre-Bosquet, N., Grobben, M., Claireaux, M., de Gast, M., Marlin, R., Chesnais, V., Diry, S., et al. (2021). Two-component spike nanoparticle vaccine protects macaques from SARS-CoV-2 infection. Cell *184*, 1188–1200.e19.
32. NCT05007951 A Phase III Study to Assess the Immunogenicity and Safety of SK SARS-CoV-2 Recombinant Nanoparticle Vaccine Adjuvanted with AS03 (GBP510) in Adults Aged 18 Years and Older. https://www.clinicaltrials.gov/ct2/show/NCT05007951.
33. Brouwer, P.J.M., Antanasijevic, A., Berndsen, Z., Yasmeen, A., Fiala, B., Bijl, T.P.L., Bontjer, I., Bale, J.B., Sheffler, W., Allen, J.D., et al. (2019). Enhancing and shaping the immunogenicity of native-like HIV-1 envelope trimers with a two-component protein nanoparticle. Nat. Commun. *10*, 4272.
34. Sliepen, K., Schermer, E., Bontjer, I., Burger, J.A., Lévai, R.F., Mundsperger, P., Brouwer, P.J.M., Tolazzi, M., Farsang, A., Katinger, D., et al. (2021). Interplay of diverse adjuvants and nanoparticle presentation of native-like HIV-1 envelope trimers. NPJ Vaccines *6*, 134.
35. Kelly, H.G., Tan, H.-X., Juno, J.A., Esterbauer, R., Ju, Y., Jiang, W., Wimmer, V.C., Duckworth, B.C., Groom, J.R., Caruso, F., et al. (2020). Self-assembling influenza nanoparticle vaccines drive extended germinal center activity and memory B cell maturation. JCI Insight *5*, 136653.
36. Arunachalam, P.S., Walls, A.C., Golden, N., Atyeo, C., Fischinger, S., Li, C., Aye, P., Navarro, M.J., Lai, L., Edara, V.V., et al. (2021). Adjuvanting a subunit COVID-19 vaccine to induce protective immunity. Nature *594*, 253–258.
37. Aide, P., Dobaño, C., Sacarlal, J., Aponte, J.J., Mandomando, I., Guinovart, C., Bassat, Q., Renom, M., Puyol, L., Macete, E., et al. (2011). Four year immunogenicity of the RTS, S/AS02(A) malaria vaccine in Mozambican children during a phase IIb trial. Vaccine *29*, 6059–6067.
38. Schödel, F., Wirtz, R., Peterson, D., Hughes, J., Warren, R., Sadoff, J., and Milich, D. (1994). Immunity to malaria elicited by hybrid hepatitis B virus core particles carrying circumsporozoite protein epitopes. J. Exp. Med. *180*, 1037–1046.
39. Schödel, F., Peterson, D., Hughes, J., Wirtz, R., and Milich, D. (1996). Hybrid hepatitis B virus core antigen as a vaccine carrier moiety: I. presentation of foreign epitopes. J. Biotechnol. *44*, 91–96.
40. Lell, B., Agnandji, S., von Glasenapp, I., Haertle, S., Oyakhiromen, S., Issifou, S., Vekemans, J., Leach, A., Lievens, M., Dubois, M.-C., et al. (2009). A randomized trial assessing the safety and immunogenicity of AS01 and AS02 adjuvanted RTS, S malaria vaccine candidates in children in Gabon. PLoS One *4*, e7611.
41. Kanekiyo, M., Ellis, D., and King, N.P. (2019). New vaccine design and delivery technologies. J. Infect. Dis. *219*, S88–S96.
42. Irvine, D.J., and Read, B.J. (2020). Shaping humoral immunity to vaccines through antigen-displaying nanoparticles. Curr. Opin. Immunol. *65*, 1–6.
43. Garrity, R.R., Rimmelzwaan, G., Minassian, A., Tsai, W.P., Lin, G., de Jong, J.J., Goudsmit, J., and Nara, P.L. (1997). Refocusing neutralizing antibody response by targeted dampening of an immunodominant epitope. J. Immunol. *159*, 279–289.
44. Pantophlet, R., Wilson, I.A., and Burton, D.R. (2003). Hyperglycosylated mutants of human immunodeficiency virus (HIV) type 1 monomeric gp120 as novel antigens for HIV vaccine design. J. Virol. *77*, 5889–5901.
45. Selvarajah, S., Puffer, B., Pantophlet, R., Law, M., Doms, R.W., and Burton, D.R. (2005). Comparing antigenicity and immunogenicity of engineered gp120. J. Virol. *79*, 12148–12163.
46. Sliepen, K., van Montfort, T., Melchers, M., Isik, G., and Sanders, R.W. (2015). Immunosilencing a highly immunogenic protein trimerization domain. J. Biol. Chem. *290*, 7436–7442.
47. Duan, H., Chen, X., Boyington, J.C., Cheng, C., Zhang, Y., Jafari, A.J., Stephens, T., Tsybovsky, Y., Kalyuzhniy, O., Zhao, P., et al. (2018). Glycan masking focuses immune responses to the HIV-1 CD4-binding site and enhances elicitation of VRC01-class precursor antibodies. Immunity *49*, 301–311.e5.
48. Bajic, G., Maron, M.J., Adachi, Y., Onodera, T., McCarthy, K.R., McGee, C.E., Sempowski, G.D., Takahashi, Y., Kelsoe, G., Kuraoka, M., and Schmidt, A.G. (2019). Influenza antigen engineering focuses immune responses to a subdominant but broadly protective viral epitope. Cell Host Microbe *25*, 827–835.e6.
49. Thornlow, D.N., Macintyre, A.N., Oguin, T.H., Karlsson, A.B., Stover, E.L., Lynch, H.E., Sempowski, G.D., and Schmidt, A.G. (2021). Altering the immunogenicity of hemagglutinin immunogens by hyperglycosylation and disulfide stabilization. Front. Immunol. *12*, 737973.
50. Eggink, D., Goff, P.H., and Palese, P. (2014). Guiding the immune response against influenza virus hemagglutinin toward the conserved stalk domain by hyperglycosylation of the globular head domain. J. Virol. *88*, 699–704.
51. Sok, D., Briney, B., Jardine, J.G., Kulp, D.W., Menis, S., Pauthner, M., Wood, A., Lee, E.-C., Le, K.M., Jones, M., et al. (2016). Priming HIV-1 broadly neutralizing antibody precursors in human Ig loci transgenic mice. Science *353*, 1557–1560.
52. Ringe, R.P., Ozorowski, G., Rantalainen, K., Struwe, W.B., Matthews, K., Torres, J.L., Yasmeen, A., Cottrell, C.A., Ketas, T.J., LaBranche, C.C., et al. (2017). Reducing V3 antigenicity and immunogenicity on soluble, native-like HIV-1 Env SOSIP trimers. J. Virol. *91*, 006777.
53. Ringe, R.P., Pugach, P., Cottrell, C.A., LaBranche, C.C., Seabright, G.E., Ketas, T.J., Ozorowski, G., Kumar, S., Schorcht, A., van Gils, M.J., et al. (2019). Closing and opening holes in the glycan shield of HIV-1 envelope glycoprotein SOSIP trimers can redirect the neutralizing antibody response to the newly unmasked epitopes. J. Virol. *93*, 016566–e1718.
54. Swanson, K.A., Rainho-Tomko, J.N., Williams, Z.P., Lanza, L., Peredelchuk, M., Kishko, M., Pavot, V., Alamares-Sapuay, J., Adhikarla, H., Gupta, S., et al. (2020). A respiratory syncytial virus (RSV) F protein nanoparticle vaccine focuses antibody responses to a conserved neutralization domain. Sci. Immunol. *5*, eaba6466.
55. Boyoglu-Barnum, S., Hutchinson, G.B., Boyington, J.C., Moin, S.M., Gillespie, R.A., Tsybovsky, Y., Stephens, T., Vaile, J.R., Lederhofer, J., Corbett, K.S., et al. (2020). Glycan repositioning of influenza hemagglutinin stem facilitates the elicitation of protective cross-group antibody responses. Nat. Commun. *11*, 791.
56. Weidenbacher, P.A., and Kim, P.S. (2019). Protect, modify, deprotect (PMD): a strategy for creating vaccines to elicit antibodies targeting a specific epitope. Proc. Natl. Acad. Sci. USA *116*, 9947–9952.
57. Tytgat, H.L.P., Lin, C.-W., Levasseur, M.D., Tomek, M.B., Rutschmann, C., Mock, J., Liebscher, N., Terasaka, N., Azuma, Y., Wetter, M., et al. (2019). Cytoplasmic glycoengineering enables biosynthesis of nanoscale glycoprotein assemblies. Nat. Commun. *10*, 5403.
58. Raja, K.S., Wang, Q., Gonzalez, M.J., Manchester, M., Johnson, J.E., and Finn, M.G. (2003). Hybrid virus-polymer materials. 1. Synthesis and properties of PEG-decorated cowpea mosaic virus. Biomacromolecules *4*, 472–476.

59. Steinmetz, N.F., and Manchester, M. (2009). PEGylated viral nanoparticles for biomedicine: the impact of PEG chain length on VNP cell interactions in vitro and ex vivo. Biomacromolecules *10*, 784–792.

60. O'Riordan, C.R., Lachapelle, A., Delgado, C., Parkes, V., Wadsworth, S.C., Smith, A.E., and Francis, G.E. (1999). PEGylation of adenovirus with retention of infectivity and protection from neutralizing antibody in vitro and in vivo. Hum. Gene Ther. *10*, 1349–1358.

61. Eto, Y., Yoshioka, Y., Ishida, T., Yao, X., Morishige, T., Narimatsu, S., Mizuguchi, H., Mukai, Y., Okada, N., Kiwada, H., and Nakagawa, S. (2010). Optimized PEGylated adenovirus vector reduces the anti-vector humoral immune response against adenovirus and induces a therapeutic effect against metastatic lung cancer. Biol. Pharm. Bull. *33*, 1540–1544.

62. Kim, P.-H., Sohn, J.-H., Choi, J.-W., Jung, Y., Kim, S.W., Haam, S., and Yun, C.-O. (2011). Active targeting and safety profile of PEG-modified adenovirus conjugated with herceptin. Biomaterials *32*, 2314–2326.

63. Vannucci, L., Falvo, E., Fornara, M., Di Micco, P., Benada, O., Krizan, J., Svoboda, J., Hulikova-Capkova, K., Morea, V., Boffi, A., and Ceci, P. (2012). Selective targeting of melanoma by PEG-masked protein-based multifunctional nanoparticles. Int. J. Nanomed. *7*, 1489–1509.

64. Molino, N.M., Bilotkach, K., Fraser, D.A., Ren, D., and Wang, S.-W. (2012). Complement activation and cell uptake responses toward polymer-functionalized protein nanocapsules. Biomacromolecules *13*, 974–981.

65. Crooke, S.N., Zheng, J., Ganewatta, M.S., Guldberg, S.M., Reineke, T.M., and Finn, M.G. (2019). Immunological properties of protein–polymer nanoparticles. ACS Appl. Bio Mater. *2*, 93–103.

66. NCT04896086 first-in-human clinical trial of a mosaic quadrivalent influenza vaccine compared with a licensed inactivated seasonal QIV, in Healthy Adults. https://clinicaltrials.gov/ct2/show/NCT04896086.

67. Schlapschy, M., Binder, U., Börger, C., Theobald, I., Wachinger, K., Kisling, S., Haller, D., and Skerra, A. (2013). PASylation: a biological alternative to PEGylation for extending the plasma half-life of pharmaceutically active proteins. Protein Eng. Des. Sel. *26*, 489–501.

68. Fleishman, S.J., Leaver-Fay, A., Corn, J.E., Strauch, E.-M., Khare, S.D., Koga, N., Ashworth, J., Murphy, P., Richter, F., Lemmon, G., et al. (2011). RosettaScripts: a scripting language interface to the Rosetta macromolecular modeling suite. PLoS One *6*, e20161.

69. Leman, J.K., Weitzner, B.D., Lewis, S.M., Adolf-Bryfogle, J., Alam, N., Alford, R.F., Aprahamian, M., Baker, D., Barlow, K.A., Barth, P., et al. (2020). Macromolecular modeling and design in Rosetta: recent methods and frameworks. Nat. Methods *17*, 665–680.

70. Goodson, R.J., and Katre, N.V. (1990). Site-directed pegylation of recombinant interleukin-2 at its glycosylation site. Biotechnology *8*, 343–346.

71. Schellenberger, V., Wang, C.-W., Geething, N.C., Spink, B.J., Campbell, A., To, W., Scholle, M.D., Yin, Y., Yao, Y., Bogin, O., et al. (2009). A recombinant polypeptide extends the in vivo half-life of peptides and proteins in a tunable manner. Nat. Biotechnol. *27*, 1186–1190.

72. Zaman, R., Islam, R.A., Ibnat, N., Othman, I., Zaini, A., Lee, C.Y., and Chowdhury, E.H. (2019). Current strategies in extending half-lives of therapeutic proteins. J. Contr. Release *301*, 176–189.

73. Falvo, E., Tremante, E., Arcovito, A., Papi, M., Elad, N., Boffi, A., Morea, V., Conti, G., Toffoli, G., Fracasso, G., et al. (2016). Improved doxorubicin encapsulation and pharmacokinetics of ferritin-fusion protein nanocarriers bearing proline, serine, and alanine elements. Biomacromolecules *17*, 514–522.

74. Lee, N.K., Lee, E.J., Kim, S., Nam, G.-H., Kih, M., Hong, Y., Jeong, C., Yang, Y., Byun, Y., and Kim, I.-S. (2017). Ferritin nanocage with intrinsically disordered proteins and affibody: a platform for tumor targeting with extended pharmacokinetics. J. Contr. Release *267*, 172–180.

75. Luginbuhl, K.M., Schaal, J.L., Umstead, B., Mastria, E.M., Li, X., Banskota, S., Arnold, S., Feinglos, M., D'Alessio, D., and Chilkoti, A. (2017). One-week glucose control via zero-order release kinetics from an injectable depot of glucagon-like peptide-1 fused to a thermosensitive biopolymer. Nat. Biomed. Eng. *1*, 0078.

76. Mesin, L., Ersching, J., and Victora, G.D. (2016). Germinal center B cell dynamics. Immunity *45*, 471–482.

77. Johansson, B.E., Bucher, D.J., and Kilbourne, E.D. (1989). Purified influenza virus hemagglutinin and neuraminidase are equivalent in stimulation of antibody response but induce contrasting types of immunity to infection. J. Virol. *63*, 1239–1246.

78. Sliepen, K., Han, B.W., Bontjer, I., Mooij, P., Garces, F., Behrens, A.-J., Rantalainen, K., Kumar, S., Sarkar, A., Brouwer, P.J.M., et al. (2019). Structure and immunogenicity of a stabilized HIV-1 envelope trimer based on a group-M consensus sequence. Nat. Commun. *10*, 2355.

79. Schorcht, A., van den Kerkhof, T.L.G.M., Cottrell, C.A., Allen, J.D., Torres, J.L., Behrens, A.-J., Schermer, E.E., Burger, J.A., de Taeye, S.W., Torrents de la Peña, A., et al. (2020). Neutralizing antibody responses induced by HIV-1 envelope glycoprotein SOSIP trimers derived from elite neutralizers. J. Virol. *94*, e01214–e01220.

80. Klinke, S., Zylberman, V., Bonomi, H.R., Haase, I., Guimarães, B.G., Braden, B.C., Bacher, A., Fischer, M., and Goldbaum, F.A. (2007). Structural and kinetic properties of lumazine synthase isoenzymes in the order Rhizobiales. J. Mol. Biol. *373*, 664–680.

81. Struwe, W.B., Chertova, E., Allen, J.D., Seabright, G.E., Watanabe, Y., Harvey, D.J., Medina-Ramirez, M., Roser, J.D., Smith, R., Westcott, D., et al. (2018). Site-specific glycosylation of virion-derived HIV-1 Env is mimicked by a soluble trimeric immunogen. Cell Rep. *24*, 1958–1966.e5.

82. Read, B.J., Won, L., Kraft, J.C., Sappington, I., Aung, A., Wu, S., Bals, J., Chen, C., Lee, K.K., Lingwood, D., et al. (2022). Mannose-binding lectin and complement mediate follicular localization and enhanced immunogenicity of diverse protein nanoparticle immunogens. Cell Rep. *38*, 110217.

83. Woodruff, M.C., Kim, E.H., Luo, W., and Pulendran, B. (2018). B cell competition for restricted T cell help suppresses rare-epitope responses. Cell Rep. *25*, 321–327.e3.

84. Lempp, F.A., Soriaga, L.B., Montiel-Ruiz, M., Benigni, F., Noack, J., Park, Y.-J., Bianchi, S., Walls, A.C., Bowen, J.E., Zhou, J., et al. (2021). Lectins enhance SARS-CoV-2 infection and influence neutralizing antibodies. Nature *598*, 342–347.

85. Johansson, B.E., Moran, T.M., and Kilbourne, E.D. (1987). Antigen-presenting B cells and helper T cells cooperatively mediate intravirionic antigenic competition between influenza A virus surface glycoproteins. Proc. Natl. Acad. Sci. USA *84*, 6869–6873.

86. Collins, K.A., Snaith, R., Cottingham, M.G., Gilbert, S.C., and Hill, A.V.S. (2017). Enhancing protective immunity to malaria with a highly immunogenic virus-like particle vaccine. Sci. Rep. *7*, 46621.

87. Datoo, M.S., Natama, M.H., Somé, A., Traoré, O., Rouamba, T., Bellamy, D., Yameogo, P., Valia, D., Tegneri, M., Ouedraogo, F., et al. (2021). Efficacy of a low-dose candidate malaria vaccine, R21 in adjuvant Matrix-M, with seasonal administration to children in Burkina Faso: a randomised controlled trial. Lancet *397*, 1809–1818.

88. Lainšček, D., Fink, T., Forstnerič, V., Hafner-Bratkovič, I., Orehek, S., Strmšek, Ž., Manček-Keber, M., Pečan, P., Esih, H., Malenšek, Š., et al. (2021). A nanoscaffolded spike-rbd vaccine provides protection against sars-cov-2 with minimal anti-scaffold response. Vaccines *9*, 431.

89. Chackerian, B. (2007). Virus-like particles: flexible platforms for vaccine development. Expert Rev. Vaccines *6*, 381–390.

90. Brody, N.I., and Siskind, G.W. (1969). Studies on antigenic competition. J. Exp. Med. *130*, 821–832.

91. Abbott, R.K., Lee, J.H., Menis, S., Skog, P., Rossi, M., Ota, T., Kulp, D.W., Bhullar, D., Kalyuzhniy, O., Havenar-Daughton, C., et al. (2018). Precursor frequency and affinity determine B cell competitive fitness in germinal centers, tested with germline-targeting HIV vaccine immunogens. Immunity *48*, 133–146.e6.

92. Dosenovic, P., Kara, E.E., Pettersson, A.-K., McGuire, A.T., Gray, M., Hartweger, H., Thientosapol, E.S., Stamatatos, L., and Nussenzweig, M.C. (2018). Anti-HIV-1 B cell responses are dependent on B cell precursor frequency and antigen-binding affinity. Proc. Natl. Acad. Sci. USA *115*, 4743–4748.

93. Abbott, R.K., and Crotty, S. (2020). Factors in B cell competition and immunodominance. Immunol. Rev. *296*, 120–131.

94. Pantophlet, R., and Burton, D.R. (2006). GP120: target for neutralizing HIV-1 antibodies. Annu. Rev. Immunol. *24*, 739–769.

95. Klasse, P.J., Ozorowski, G., Sanders, R.W., and Moore, J.P. (2020). Env exceptionalism: why are HIV-1 Env glycoproteins atypical immunogens? Cell Host Microbe *27*, 507–518.

96. Lavinder, J.J., Hoi, K.H., Reddy, S.T., Wine, Y., and Georgiou, G. (2014). Systematic characterization and comparative analysis of the rabbit immunoglobulin repertoire. PLoS One *9*, e101322.

97. McCoy, L.E., van Gils, M.J., Ozorowski, G., Messmer, T., Briney, B., Voss, J.E., Kulp, D.W., Macauley, M.S., Sok, D., Pauthner, M., et al. (2016). Holes in the glycan shield of the native HIV envelope are a target of trimer-elicited neutralizing antibodies. Cell Rep. *16*, 2327–2338.

98. Creanga, A., Gillespie, R.A., Fisher, B.E., Andrews, S.F., Lederhofer, J., Yap, C., Hatch, L., Stephens, T., Tsybovsky, Y., Crank, M.C., et al. (2021). A comprehensive influenza reporter virus panel for high-throughput deep profiling of neutralizing antibodies. Nat. Commun. *12*, 1722.

99. Goddard, T.D., Huang, C.C., Meng, E.C., Pettersen, E.F., Couch, G.S., Morris, J.H., and Ferrin, T.E. (2018). UCSF ChimeraX: meeting modern challenges in visualization and analysis. Protein Sci. *27*, 14–25.

100. Murray, A.N., Chen, W., Antonopoulos, A., Hanson, S.R., Wiseman, R.L., Dell, A., Haslam, S.M., Powers, D.L., Powers, E.T., and Kelly, J.W. (2015). Enhanced aromatic sequons increase oligosaccharyltransferase glycosylation efficiency and glycan homogeneity. Chem. Biol. *22*, 1052–1062.

101. Huang, Y.-W., Yang, H.-I., Wu, Y.-T., Hsu, T.-L., Lin, T.-W., Kelly, J.W., and Wong, C.-H. (2017). Residues comprising the enhanced aromatic sequon influence protein N-glycosylation efficiency. J. Am. Chem. Soc. *139*, 12947–12955.

102. Tiller, T., Meffre, E., Yurasov, S., Tsuiji, M., Nussenzweig, M.C., and Wardemann, H. (2008). Efficient generation of monoclonal antibodies from single human B cells by single cell RT-PCR and expression vector cloning. J. Immunol. Methods *329*, 112–124.

103. Langowski, M.D., Khan, F.A., Bitzer, A.A., Genito, C.J., Schrader, A.J., Martin, M.L., Soto, K., Zou, X., Hadiwidjojo, S., Beck, Z., et al. (2020). Optimization of a Plasmodium falciparum circumsporozoite protein repeat vaccine using the tobacco mosaic virus platform. Proc. Natl. Acad. Sci. USA *117*, 3114–3122.

104. Bloom, J.D., Gong, L.I., and Baltimore, D. (2010). Permissive secondary mutations enable the evolution of influenza oseltamivir resistance. Science *328*, 1272–1275.

105. Crawford, K.H.D., Eguia, R., Dingens, A.S., Loes, A.N., Malone, K.D., Wolf, C.R., Chu, H.Y., Tortorici, M.A., Veesler, D., Murphy, M., et al. (2020). Protocol and reagents for pseudotyping lentiviral particles with SARS-CoV-2 spike protein for neutralization assays. Viruses *12*, 513.

106. McCallum, M., De Marco, A., Lempp, F.A., Tortorici, M.A., Pinto, D., Walls, A.C., Beltramello, M., Chen, A., Liu, Z., Zatta, F., et al. (2021). N-terminal domain antigenic mapping reveals a site of vulnerability for SARS-CoV-2. Cell *184*, 2332–2347.e16.

107. Sauer, M.M., Tortorici, M.A., Park, Y.-J., Walls, A.C., Homad, L., Acton, O.J., Bowen, J.E., Wang, C., Xiong, X., de van der Schueren, W., et al. (2021). Structural basis for broad coronavirus neutralization. Nat. Struct. Mol. Biol. *28*, 478–486.

CellPress
OPEN ACCESS

## STAR★METHODS

### KEY RESOURCES TABLE

| REAGENT or RESOURCE | SOURCE | IDENTIFIER |
|---|---|---|
| Antibodies | | |
| Horse anti-mouse HRP | Cell Signaling Technology | Cat# 7076S RRID:AB_330924 |
| Mouse anti-myc mAb | Cell Signaling Technology | Cat# 2276S RRID:AB_331783 |
| Goat anti-mouse IgG+IgM | Jackson ImmunoResearch | Cat# 115-005-068 RRID:AB_2338453 |
| Mouse IgG1 lambda isotype control | BD Biosciences | Cat# 553485 RRID:AB_479649 |
| Anti-mouse Fc Block | BD Biosciences | Cat# 553142 RRID:AB_394657 |
| Anti-mouse B220 BUV737 | BD Biosciences | Cat# 612838 RRID:AB_2738813 |
| Anti-mouse CD3 PerCP-Cy5.5 | BD Biosciences | Cat# 551163 RRID:AB_394082 |
| Anti-mouse CD138 BV650 | BD Biosciences | Cat# 564068 RRID:AB_2738574 |
| Anti-mouse CD38 Alexa Fluor 700 | Thermo Fisher Scientific | Cat# 56-0381-82 RRID:AB_657740 |
| Anti-mouse GL7 ef450 | Thermo Fisher Scientific | Cat# 48-5902-82 RRID:AB_10870775 |
| Anti-mouse IgM BV786 | BD Biosciences | Cat# 743328 RRID:AB_2741429 |
| Anti-mouse IgD BUV395 | BD Biosciences | Cat# 565988 RRID:AB_2737433 |
| Anti-mouse CD73 PE-Cy7 | Thermo Fisher Scientific | Cat# 25-0731-82 RRID:AB_10853348 |
| Anti-mouse CD80 BV605 | BD Biosciences | Cat# 563052 RRID:AB_273795 |
| Biological samples | | |
| BALB/c mice | Jackson Laboratory | Cat# 000651 |
| Chemicals, peptides, and recombinant proteins | | |
| AddaVax adjuvant | InvivoGen | Cat# vac-adx-10 |
| ABTS | ThermoFisher | Cat# 37615 |
| TMB | SeraCare | Cat# 5120-0083 |
| EZ-Link Sulfo-NHS-LC Biotinylation Kit | ThermoFisher | Cat# 21435 |
| Streptavidin-APC | Agilent | Cat# PJ27S-1 |
| Streptavidin-PE | Agilent | Cat# PJRS25-1 |
| Anti-PE Micro-Beads | Miltenyi Biotec | Cat# 130-048-801 |
| Anti-APC Micro-Beads | Miltenyi Biotec | Cat# 130-090-855 |
| DyLight 755 Antibody Labeling Kit | ThermoFisher | Cat# 84538 |
| Alexa Fluor 647 Protein Labeling Kit | ThermoFisher | Cat# A20173 |
| Experimental models: Cell lines | | |
| Expi293F | ThermoFisher | Cat# A14527 |
| MDCK-SIAT-PB1 | Creanga et al.[98] | N/A |
| Recombinant DNA | | |
| See Table S1 for amino acid sequences. | | |
| Software and algorithms | | |
| UCSF ChimeraX | Goddard et al.[99] | https://www.rbvi.ucsf.edu/chimerax/ |
| Prism | GraphPad | https://www.graphpad.com/scientific-software/prism/ |
| FlowJo v10 | FlowJo | https://www.flowjo.com |
| RosettaScripts “sugarcoat” code | Adolf-Bryfogle et al.[1] | N/A |
| Other | | |
| EM supplies 300 mesh grids | Ted Pella | Cat# 01843-F |
| Filter paper | Cytiva | Cat# 1004047 |
| Uranyl formate | SPI Chem | Cat# 02545-AA |
| Superdex 200 Increase SEC column | Cytiva | Cat# 28-9909-44 |
| Superose 6 Increase SEC column | Cytiva | Cat# 29091596 |

(*Continued on next page*)

| Continued | | |
|---|---|---|
| REAGENT or RESOURCE | SOURCE | IDENTIFIER |
| Talon resin | TaKaRa | Cat# 635652 |
| Excel resin | Cytiva | Cat# 17371203 |
| Isoflurane | USP Patterson | Cat# 07-893-1389 |
| EndoSafe LAL Test Cartridges | Charles River Labs | Cat# PTS20005F |
| Lemo21(DE3) | New England BioLabs | Cat# C2528J |
| Isopropyl-B-D-thiogalactoside (IPTG) | Sigma-Aldrich | Cat# I6758 |
| Kanamycin Sulfate | Sigma-Aldrich | Cat# K1876 |
| HisTrap FF | Cytiva | Cat#17525501 |

## RESOURCE AVAILABILITY

### Lead contact

Further information and requests for resources and reagents should be directed to and will be fulfilled by the Lead Contact, Neil King (neil@ipd.uw.edu).

### Materials availability

All reagents will be made available on request after completion of a Materials Transfer Agreement.

### Data and code availability

- All data supporting the findings of this study are found within the paper and its supplemental information, and are available from the lead contact upon request.
- Code used for this study has been reported elsewhere,[1] and the XML code for designing predicted N-linked glycosylation sites (PNGS) into proteins is provided as Supplementary Material.
- Any additional information required to reanalyze the data reported in this paper is available from the lead contact upon request.

## EXPERIMENTAL MODEL AND SUBJECT DETAILS

### Cell lines

Expi293F cells are derived from the HEK293F cell line (Life Technologies). Expi293F cells were grown in Expi293 Expression Medium (Life Technologies), cultured at 36.5°C with 8% $CO_2$ and shaking at 150 rpm HEK293T/17 is a female human embryonic kidney cell line (ATCC). VeroE6-TMPRSS2 cells are an African Green monkey Kidney cell line expressing TMPRSS2.[84] Adherent cells were cultured at 37°C with 5% $CO_2$ in flasks with DMEM +10% FBS (Hyclone) + 1% penicillin-streptomycin. Adherent cells were not tested for mycoplasma contamination nor authenticated.

### Mice

Female BALB/c mice (Stock # 000,651, BALB/c cByJ mice) four weeks old were obtained from Jackson Laboratory, Bar Harbor, Maine, and maintained at the Comparative Medicine Facility at the University of Washington, Seattle, WA, accredited by the American Association for the Accreditation of Laboratory Animal Care International (AAALAC). Animal procedures were performed under the approvals of the Institutional Animal Care and Use Committee (IACUC) of the University of Washington, Seattle, WA.

## METHOD DETAILS

### Computational design of glycosylated proteins

Detailed methods and code are reported elsewhere.[1] Briefly, all possible residues on the outward facing surfaces of the I53_dn5A pentamer and I53_dn5B trimer when assembled into I53_dn5 nanoparticles were manually selected as candidate locations for designing in an NxT/S PNGS. Next, the CreateGlycanSequonMover in Rosetta was used to sequentially knock-in a single NxT/S sequon at these selected locations and obtain calculated energies of the new protein structure using the Rosetta score function. Both typical and enhanced sequons, which include an aromatic amino acid in the N-2 position to potentially increase glycosylation efficiency,[100,101] were attempted at each position. Protein structures were first scored by Rosetta without a model glycan tree present to eliminate any potential interference of the glycan atoms. To filter out bad designs, outputs with a “total_energy” of >500 and an RMSD >0.25 Å and >0.40 Å compared to the original I53_dn5A and I53_dn5B scaffolds, respectively, were discarded. The re-designed protein structures that passed this filtering step were then glycosylated using the SimpleGlycosylateMover with a model tri-antennary Man9 N-linked glycan, modeled using the GlycanTreeModeler, and scored by Rosetta. A second round of filtering

was performed using the same criteria as above. After proteins with a single PNGS were experimentally screened for expression and glycan occupancy (see below), combinations of PNGS were designed using the same computational pipeline. The XML file for this combinatorial selection is provided as Supplementary Material. The recently reported I53-50A_4gly subunit[82] was used to generate the glycosylated RSV F-I53-50 immunogen shown in Figures S2L–S2O.

## Gene synthesis and vector construction

For each design that resulted from the above computational pipeline, the final construct contained an N-terminal signal peptide derived from bovine prolactin (MDSKGSSQKGSRLLLLLVVSNLLLPQGVLA) and C-terminal myc and hexahistidine tags (LEEQKLISEEDLHHHHHH). These constructs and others used in this study were cloned by GenScript into the pCMV/R (VRC 8400) mammalian expression vector using the restriction sites Xba1 and AvrII. Preparation of plasmids for expression of the following proteins have been previously described: I53_dn5A pentamer and I53_dn5B trimer,[30] I53-50B.4PT1 pentamer,[29] influenza H1MI15 fusion to I53_dn5B trimer,[10] HIV-1 ConM Env fusion to I53-50A trimer,[33] HIV-1 AMC009 Env trimer,[78] RSV DS-Cav1 fusion to I53-50A trimer,[8] SARS-CoV-2 RBD fusion to I53-50A trimer,[9] and SARS-CoV-2 Spike HexaPro trimer.[20] HIV-1 AMC009 Env trimer was fused to I53-50A trimers as described in.[33] The amino acid sequences for all proteins used in this study are provided in Table S1.

## Microbial protein expression and purification

The nanoparticle components I53-50A and I53-50B.4.PT1,[29] and I53_dn5A and I53_dn5B,[30] were expressed in Lemo21(DE3) (NEB) in LB (10 g Tryptone, 5 g Yeast Extract, 10 g NaCl) and grown in 2 L baffled shake flasks. Cells were grown at 37°C to an OD600–0.8, and then induced with 1 mM IPTG. Expression temperature was reduced to 18°C and the cells were shaken for ~16 h. The cells were harvested and lysed by microfluidization using a Microfluidics M110P at 18,000 psi in 50 mM Tris, 500 mM NaCl, 30 mM imidazole, 1 mM PMSF, (with 0.75% CHAPS only for I53-50 proteins). Lysates were clarified by centrifugation at 24,000 g for 30 min and applied to a 2.6 × 10 cm Ni Sepharose 6 FF column (Cytiva) for purification by IMAC on an AKTA Avant150 FPLC system (Cytiva). Protein of interest was eluted over a linear gradient of 30 mM–500 mM imidazole in a background of 50 mM Tris pH 8, 500 mM NaCl, (with 0.75% CHAPS only for I53-50 proteins) buffer. Peak fractions were pooled, concentrated in 10K MWCO centrifugal filters (Millipore), sterile filtered (0.22 μm) and applied to a Superdex 200 Increase 10/300 SEC column (Cytiva) using 50 mM Tris pH 8, 500 mM NaCl, (with 0.75% CHAPS only for I53-50 proteins) buffer. After sizing, bacterial-derived components were tested to confirm low levels of endotoxin before using for nanoparticle assembly.

## Mammalian protein expression and purification

Small-scale 2.0 mL cultures of Expi293F cells were grown in suspension to a density of $3.0\times10^6$ cells per mL and transiently transfected using PEI-MAX (Polyscience) and cultivated for 5 days in Expi293F expression medium (Life Technologies) at 37°C, 70% humidity, 8% CO2, and rotating at 150 rpm. Supernatants were clarified by centrifugation (5 min at 4000 rcf), PDADMAC solution was added to a final concentration of 0.0375% (Sigma Aldrich, #409014), and a final spin was performed (5 min at 4000 rcf). Supernatants were concentrated using a 5 kDa MWCO spin filter (Sartorius) to a final volume of ~50 μL. These concentrated supernatants were then assessed for protein expression by Western blot using an anti-myc mouse primary antibody and an anti-mouse HRP-conjugated goat secondary antibody. Glycan occupancy for each protein design was assessed by increased molecular weight gel shifts on the Western blots compared to the unglycosylated parent protein.

For large-scale protein expression, 800 mL cultures of Expi293F cells were transiently transfected and cultivated for 5 days as described above. Proteins were purified from clarified supernatants via a batch bind method where Talon cobalt affinity resin (Takara) was added to supernatants and allowed to incubate for 15 min with gentle shaking. Resin was isolated using 0.2 μm vacuum filtration and transferred to a gravity column, where it was washed with 20 mM Tris pH 8.0, 300 mM NaCl, and protein was eluted with 3 column volumes of 20 mM Tris pH 8.0, 300 mM NaCl, 300 mM imidazole. This batch bind process was repeated a second time on the supernatant flow-through from the filtration step. Eluate with protein was concentrated to ~2 mL using a 30 kDa MWCO Amicon concentrator (Millipore Sigma). The concentrated sample was sterile filtered (0.2 μm) and applied to a Superdex 200 Increase 10/300 SEC column (Cytiva) using 25 mM Tris pH 8.0, 150 mM NaCl, 0.75% CHAPS, 5% glycerol buffer.

Env-I53-50A constructs together with a plasmid expressing furin were transfected into Expi293F cells using PEI-MAX and cultured for 6 days. Furin was added to ensure optimal furin-mediated cleavage of Env (ConM-I53-50A:furin ratio was 3:1, AMC009-I53-50A:furin ratio was 2:1). Cells were spun down and supernatants filtered through a 0.2 μm Steritop filter. Env-I53-50A proteins were purified by running the clarified supernatant over a PGT145 bNAb-affinity chromatography column. Eluted proteins were concentrated using vivaspin 100 kDa spin columns. Concentrated proteins were subsequently applied to a Superose 6 increase 10/300 GL column (Cytiva) to remove aggregated proteins using a buffer of 25mM Tris pH 8.0, 125 mM NaCl, 5% glycerol. I53-50B.4PT1 was added in a 1:1 ratio and incubated at 4°C overnight. Assembled particles were again applied to a Superose 6 increase 10/300 GL column (GE healthcare) to remove unassembled components. Particles were buffer-exchanged into PBS with 250 mM sucrose by dialysis at 4°C overnight, followed by a second dialysis step of 4 h, using a Slide-A-Lyzer MINI dialysis device (20 kDa cutoff, ThermoFisher Scientific). The 250 mM sucrose was added to increase recovery after freeze-thawing.

### PEG-maleimide to HS-protein coupling

Protein with reduced unpaired cysteines was first purified by size exclusion chromatography (SEC) using buffer that contained 1 mM TCEP, and then SEC-purified again to exchange buffer with HEPES coupling buffer (pH 7.4, 20 mM HEPES, 150 mM NaCl, 1 mM EDTA, 0.75% CHAPS). Using a freshly prepared 10 mM PEG-maleimide solution in HEPES coupling buffer, a 1.0 mL maleimide-thiol coupling reaction was prepared at 5:1 PEG:Cys (mol/mol) and a 50 μM final protein concentration. This reaction was incubated with rocking at ambient temperature for 3 h, then overnight at 4°C. The reaction was quenched by adding reduced glutathione (GSH) to 2 mM. Unreacted PEG was removed using SEC.

### *In vitro* nanoparticle assembly and purification

The protein concentration of individual nanoparticle components (e.g., I53_dn5A pentamer and I53_dn5B trimer, or I53-50A trimer and I53-50B.4PT1 pentamer) was determined by measuring 280 nm absorbance using a UV/vis spectrophotometer (Agilent Cary 8454) and estimated extinction coefficients.[8] Particle assembly was performed by adding equimolar amounts of trimer and pentamer components to reach a final protein concentration of 20 μM (10 μM for each individual component) and resting on ice for at least 30 min. Assembled particles were sterile filtered (0.2 μm) immediately before SEC purification using a Superose 6 Increase 10/300 GL column or a HiLoad 26/600 Superdex 200 pg column for the RBD-I53-50 nanoparticle immunogen.

### Negative-stain electron microscopy

A sample volume of 3 μL at a concentration of 70 μg/mL protein in 50 mM Tris pH 8, 150 mM NaCl, 5% v/v glycerol was applied to a freshly glow-discharged 300-mesh copper grid (Ted Pella) and incubated on the grid for 1 min. The grid was then dipped in a 40 μL droplet of water, excess liquid was blotted away with filter paper (Whatman), the grid was dipped into 3 μL of 0.75% w/v uranyl formate stain, stain was immediately blotted off with filter paper, then the grid was dipped again into another 3 μL of stain and incubated for ~30 seconds. Finally, the stain was blotted away and the grids were allowed to dry for 1 minute prior to storage or imaging. Prepared grids were imaged in a Talos model L120C transmission electron microscope using a Gatan camera at 57,000×.

### Antigenic characterization

ELISA was used to measure binding of HA-foldon, HA-1na0C3int2, HA-ferritin, HA-I53_dn5, HA-I53-dn5_ABgly, and HA-I53_dn5_2C2kPEG to monoclonal antibodies CR9114 and 5J8 using the ELISA method described below. Monoclonal antibodies were serially diluted from 300 to 0.5 ng/mL.

### Dynamic light scattering

Dynamic light scattering (DLS) was used to measure the hydrodynamic diameter of nanoparticle immunogens on a DynaPro NanoStar instrument (Wyatt Technologies). 2 μL of 0.1 mg/mL protein was applied to a quartz cuvette to obtain intensity measurements from 10 acquisitions of 10 s each. Increased viscosity due to 5% glycerol in the buffer was accounted for by the software.

### Endotoxin measurements

Endotoxin levels in immunogen samples were measured using the EndoSafe Nexgen-MCS System (Charles River). Samples were diluted 1:100 in Endotoxin-free LAL reagent water, and applied into wells of an EndoSafe LAL reagent cartridge. Endotoxin content was analyzed using Charles River EndoScan-V software, which automatically back-calculates for the 1:100 dilution factor. Endotoxin values reported as EU/mL were converted to EU/mg based on protein concentration obtained by UV-Vis measurements. All endotoxin values were <100 EU/mg.

### Mouse immunizations and sera collection

Mice were inoculated with 0.9 μg HA and/or 0.6 μg I53_dn5 scaffold (1.2 μg I53_dn5 scaffold for the HA-I53_dn5_ABgly group due to 50% HA valency) for the scaffold masking experiments (Figures 2 and S2); $7.24 \times 10^{-5}$ μmol antigen (equal to 5 μg HIV-1 Env) and $1.21 \times 10^{-6}$ μmol (3 μg) I53-50 scaffold for the antigen immunodominance experiment (Figures 3, 4, S4, and S5); and the indicated doses for RBD, HIV-1 ConM, and I53-50 in Figures 5 and S6. Prior to inoculation, immunogen suspensions were gently mixed 1:1 (vol/vol) with AddaVax adjuvant (Invivogen, San Diego, CA). Mice were injected intramuscularly into the gastrocnemius muscle of each hind leg using a 27-gauge needle with 50 μL per injection site (100 μL total) of immunogen under isoflurane anesthesia. For sera collection, mice were bled via submental venous puncture 2 weeks following each inoculation. Serum was isolated from hematocrit via centrifugation at 2,000 g for 10 min, and stored at -80°C until use.

### Serum antibody ELISA

The protocol was adapted from Tiller et al.[102] First, protein or goat anti-mouse IgG+IgM (Jackson ImmunoResearch, 115-005-068) was incubated for 1 h on 96-well Nunc MaxiSorp plates (Thermo Scientific) (2.0 μg/mL, 50 μL per well). Then, 200 μL of Tris-Buffered Saline Tween (TBST: 25 mM Tris pH 8.0, 150 mM NaCl, 0.05% (v/v) Tween 20) with 2% (w/v) BSA was added to each well and incubated for 1 h. Plates were washed 3× in TBST using a robotic plate washer (BioTek). Then, 50 μL of serum dilutions starting at 1:100 and serially diluting 5-fold seven times using TBST with 2% (w/v) BSA (8 total dilutions) were added to each well and incubated for 1 h. In wells with anti-mouse IgG capture antibody, mouse IgG lambda control (BD Pharminogen, 553,485) was serially diluted from 500 to

0.5 ng/mL in TBST in triplicate and 50 μL of each dilution incubated for 1 h. After washing plates 3× with TBST, 50 μL of anti-mouse HRP-conjugated goat secondary antibody (CellSignaling Technology) diluted 1:2,000 in TBST with 2% (w/v) BSA was incubated in each well for 1 h. Following a final 3× TBST plate wash, 100 μL of ABTS (2,2′-Azinobis [3-ethylbenzothiazoline-6-sulfonic acid]-diammonium salt, Thermo Scientific) or TMB (3,3′,5′,5-tetramethylbenzidine, SeraCare) was added to each well and rested for 30 or 2 min, respectively. TMB was quenched with 100 μL of 1 N HCl. Absorbance at 405 or 450 nm, respectively, was immediately collected for each well on a SpectraMax M5 plate reader (Molecular Devices). All steps were performed at ambient temperature. Data were plotted in Prism (GraphPad) to determine AUC values. A logarithmic equation fit to the linear portion of the sigmoidal curve of the mouse IgG control was used to calculate concentration (mg/mL) of IgG in mouse sera for anti-I53_dn5 and anti-HA titers.

### Serum antibody avidity/chaotropic ELISA

The protocol was adapted from Langowski et al.[103] First, recombinant I53_dn5 nanoparticle or H1 MI15-foldon protein was incubated for 1 h on 96-well Nunc MaxiSorp plates (Thermo Scientific) (2.0 μg/mL, 50 μL per well). Then, 200 μL of Tris-Buffered Saline Tween (TBST: 25 mM Tris pH 8.0, 150 mM NaCl, 0.05% (v/v) Tween 20) with 2% (w/v) BSA was added to each well and incubated for 1 hr. Plates were washed 3× in TBST using a robotic plate washer (BioTek). Then, 50 μL of a 1:2,500 serum dilution in TBST with 2% (w/v) BSA was added to each well and incubated for 1 h. To test for avidity, 50 μL of 2 M sodium thiocyanate (NaSCN) or PBS (control) was added to wells for 15 min. After washing plates 3× with TBST, 50 μL of anti-mouse HRP-conjugated goat secondary antibody (CellSignaling Technology) diluted 1:2,000 in TBST with 2% (w/v) BSA was incubated in each well for 1 hr. Following a final 3× TBST plate wash, 100 μL of TMB (SeraCare) was added to each well and rested for 2 min before quenching with 100 μL of 1 N HCl. Absorbance at 450 nm was immediately collected for each well on a SpectraMax M5 plate reader (Molecular Devices). All steps were performed at ambient temperature. Percentage OD450 in the corresponding NaSCN/PBS wells were used to determine the avidity index.

### Ni-NTA-capture ELISA

The protocol was adapted from Brouwer et al.[33] First, 50 μL of 6.5 nM His-tagged protein per well was incubated for 1 h in 96-well Ni-NTA plates (Qiagen). Then, 200 μL of Tris-Buffered Saline Tween (TBST: 25 mM Tris pH 8.0, 150 mM NaCl, 0.05% v/v Tween 20) with 2% (w/v) BSA was added to each well and incubated for 1 h. Plates were washed 3× in TBST using a robotic plate washer (BioTek). Then, 50 μL of serum dilutions starting at 1:100 and serially diluting 5-fold seven times using TBST with 2% (w/v) BSA (8 total dilutions) were added to each well and incubated for 1 h. After washing plates 3× with TBST, 50 μL of anti-mouse HRP-conjugated goat secondary antibody (CellSignaling Technology) diluted 1:2,000 in TBST with 2% (w/v) BSA was incubated in each well for 1 h. Following a final 3× TBST plate wash, 100 μL of TMB (SeraCare) was added to each well and rested for 2 min, then 100 μL of 1 N HCl was added to each well to quench the reaction. Absorbance at 450 nm was immediately collected for each well on a SpectraMax M5 plate reader (Molecular Devices). Data were plotted in Prism (GraphPad) to determine AUC values. All steps were performed at ambient temperature.

### Sera immunodepletion

Depletion antigen (I53_dn5) was added to reach a final concentration of 0.3 mg/mL in the starting 1:100 serum dilution used in ELISA and incubated for 15 min at room temperature. Then, serial dilutions and the ELISA procedure was performed as described above.

### Reporter-based microneutralization assay

Reporter viruses were prepared as previously described.[98] In brief, H1N1 virus was made with a modified PB1 segment expressing the TdKatushka reporter gene (R3ΔPB1) and propagated in MDCK-SIAT-PB1 cells, while H5N1 reporter virus was made with a modified HA segment expressing the reporter (R3ΔHA) and produced in cells stably expressing H5 HA. Virus stocks were stored at −80°C. Mouse sera were treated with receptor destroying enzyme (RDE II; Denka Seiken) and heat-inactivated before use in neutralization assays. Immune sera was serially diluted and incubated for 1 h at 37°C with pre-titrated virus. Serum-virus mixtures were then transferred to 96-well plates (PerkinElmer), and $1.0\times10^4$ MDCK-SIAT1-PB1 cells[98,104] were added into each well. After overnight incubation at 37°C, the number of fluorescent cells in each well was counted automatically using a Celigo image cytometer (Nexcelom Biosciences). IC50 values, defined as the serum dilution or antibody concentration that gives 50% reduction in virus-infected cells, were calculated from neutralization curves using a four-parameter nonlinear regression model and plotted with GraphPad Prism.

### Pseudovirus production

D614G SARS-CoV-2 S[105] pseudotyped vesicular stomatitis viruses (VSVs) were prepared as described previously.[106,107] Briefly, 293T cells in DMEM supplemented with 10% FBS, 1% PenStrep seeded in 10-cm dishes were transfected with the plasmid encoding for the S glycoprotein using lipofectamine 2000 (Life Technologies) following manufacturer's indications. One day post-transfection, cells were infected with VSV(G*ΔG-luciferase) and after 2 h were washed five times with DMEM before adding medium supplemented with anti-VSV-G antibody (I1- mouse hybridoma supernatant, CRL- 2700, ATCC). Virus pseudotypes were harvested 18-24 h post-inoculation, clarified by centrifugation at 2,500 × g for 5 min, filtered through a 0.45 μm cut off membrane, concentrated 10 times with a 30 kDa cut off membrane, aliquoted and stored at −80°C.

### Pseudovirus neutralization

VeroE6-TMPRSS2 cells[84] were cultured in DMEM with 10% FBS (Hyclone), 1% PenStrep, and 8 μg/mL puromycin with 5% $CO_2$ in a 37°C incubator (Caron-VWR). One day prior to infection, 96-well plates were plated with 20,000 cells. The following day, cells were checked to be at 80% confluence. In an empty half-area 96-well plate a 1:3 serial dilution of sera was made in DMEM and then diluted pseudovirus was added to the serial dilution and incubated at room temperature for 30-60 min. After incubation, the sera-virus mixture was added to the cells at 37°C and 2 hours post-infection, 40 μL 20% FBS-2% PenStrep DMEM was added. After 17-20 hours 40 μL/well of One-Glo-EX substrate (Promega) was added to the cells and incubated in the dark for 5-10 min prior to reading on a BioTek plate reader. Measurements were done in at least duplicate. Relative luciferase units were plotted and normalized in Prism (GraphPad). Nonlinear regression of log(inhibitor) versus normalized response was used to determine IC50 values from curve fits.

### Tetramer production

Recombinant I53_dn5A pentamer, I53_dn5B trimer, and H1 MI15 hemagglutinin trimer were biotinylated using the EZ-Link Sulfo-NHS-LC Biotinylation Kit (ThermoFisher). Biotinylated protein was then incubated with differing amounts of streptavidin-PE (Prozyme) and probed with SA-AF680 (Invitrogen) to determine the ratio of biotin to streptavidin at which there was excess biotin available for SA-AF680 to bind. This ratio was used to determine the concentration of biotinylated protein, allowing for calculation of the amount of SA-PE required to create a 6:1 molar ratio of protein protomer to SA-PE. Biotinylated HA was incubated with SA-APC for 30 min at room temperature and purified on a Superose 6 Increase 10/300 GL size exclusion column (Cytiva), and the tetramer fraction was centrifuged in a 100 kDa molecular weight cutoff Amicon Ultra filter (Millipore). The tetramer concentration was determined by measuring the absorbance of APC at 650 nm I53_dn5A and I53_dn5B proteins were biotinylated and tetramerized with SA-PE in the same manner, and the concentration was determined by measuring the absorbance of PE at 565 nm. The APC decoy reagent was generated by conjugating SA-APC to Dylight 755 using a DyLight 755 antibody labeling kit (ThermoFisher), washing and removing unbound DyLight 755, and incubating with excess of an irrelevant biotinylated His-tagged protein. The PE decoy was generated in the same manner, by conjugating SA-PE to Alexa Fluor 647 with an AF647 antibody labeling kit (ThermoFisher).

### Mouse immunization, cell enrichment, and flow cytometry

For phenotyping B cells, 6-week old female BALB/c mice, three per dosing group, were immunized intramuscularly with 50 μL per injection site of immunogen formulations mixed 1:1 (vol/vol) with AddaVax adjuvant on day 0. All experimental mice were euthanized for harvesting of inguinal and popliteal lymph nodes on day 11. The experiment was repeated twice. Popliteal and inguinal lymph nodes were collected and pooled for individual mice. Cell suspensions were prepared by mashing lymph nodes and filtering through 100 μm Nitex mesh. Cells were resuspended in PBS containing 2% FBS and Fc block (2.4G2), and were incubated with 10 nM decoy tetramers at room temperature for 20 min. I53_dn5A-PE tetramer and HA-APC tetramer, or I53_dn5B-PE tetramer and HA-APC tetramer, were added at a concentration of 10 nM and incubated on ice for 20 min. Cells were washed, incubated with anti-PE and anti-APC magnetic beads on ice for 30 min, then passed over magnetized LS columns (Miltenyi Biotec). Bound B cells were stained with anti-mouse B220 (BUV737), CD3 (PerCP-Cy5.5), CD138 (BV650), CD38 (Alexa Fluor 700), GL7 (eFluor 450), IgM (BV786), IgD (BUV395), CD73 (PE-Cy7), and CD80 (BV605) on ice for 20 min. Cells were run on a Cytek Aurora and analyzed using FlowJo software (Treestar). Cell counts were determined using Accucheck cell counting beads.

### Statistical analysis

Multi-group comparisons were performed using the Brown-Forsythe one-way ANOVA test and Dunnett's T3 post hoc analysis in Prism 9 (GraphPad) unless mentioned otherwise. All correlations were two-tailed Spearman's correlations based on ranks. Differences were considered significant when p values were less than 0.05.

**Cell Reports Medicine, Volume *3***

## Supplemental information

# Antigen- and scaffold-specific antibody responses to protein nanoparticle immunogens

**John C. Kraft, Minh N. Pham, Laila Shehata, Mitch Brinkkemper, Seyhan Boyoglu-Barnum, Kaitlin R. Sprouse, Alexandra C. Walls, Suna Cheng, Mike Murphy, Deleah Pettie, Maggie Ahlrichs, Claire Sydeman, Max Johnson, Alyssa Blackstone, Daniel Ellis, Rashmi Ravichandran, Brooke Fiala, Samuel Wrenn, Marcos Miranda, Kwinten Sliepen, Philip J.M. Brouwer, Aleksandar Antanasijevic, David Veesler, Andrew B. Ward, Masaru Kanekiyo, Marion Pepper, Rogier W. Sanders, and Neil P. King**

Cell Reports Medicine, Volume *3*

# Supplemental information

# Antigen- and scaffold-specific antibody responses to protein nanoparticle immunogens

**John C. Kraft, Minh N. Pham, Laila Shehata, Mitch Brinkkemper, Seyhan Boyoglu-Barnum, Kaitlin R. Sprouse, Alexandra C. Walls, Suna Cheng, Mike Murphy, Deleah Pettie, Maggie Ahlrichs, Claire Sydeman, Max Johnson, Alyssa Blackstone, Daniel Ellis, Rashmi Ravichandran, Brooke Fiala, Samuel Wrenn, Marcos Miranda, Kwinten Sliepen, Philip J.M. Brouwer, Aleksandar Antanasijevic, David Veesler, Andrew B. Ward, Masaru Kanekiyo, Marion Pepper, Rogier W. Sanders, and Neil P. King**

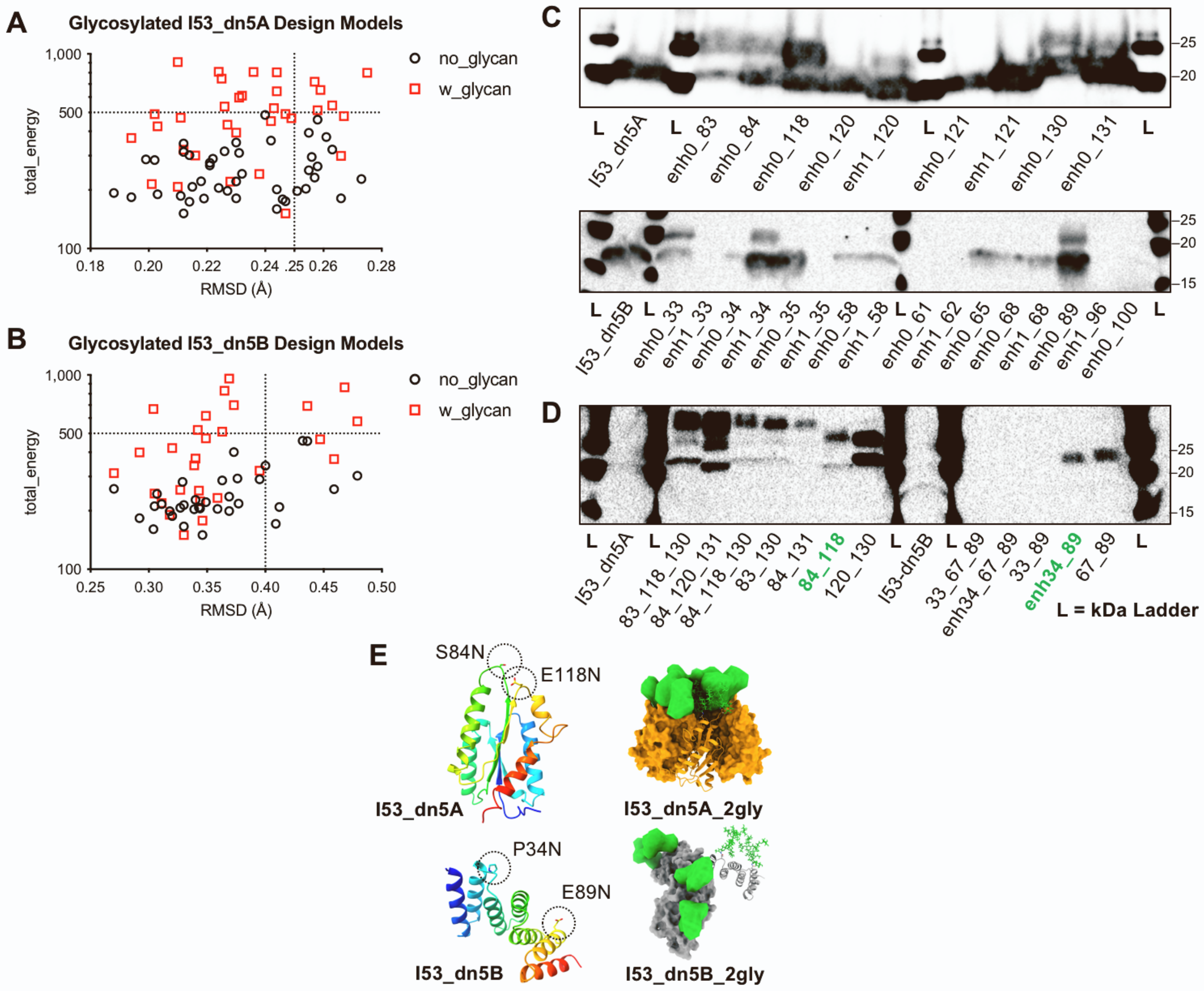


**Figure S1. Design of Glycosylated I53_dn5 Nanoparticle Scaffolds, Related to Figure 1**

(A and B) Rosetta total_energy vs. backbone (Cα) root mean square deviation (RMSD, Å) for design models of glycosylated I53_dn5A pentamers (A) and I53_dn5B trimers (B). Dotted lines indicate filter cut-offs for selection of designs to experimentally test for protein expression and glycosylation.

(C and D) Reducing western blots of concentrated cell supernatants for single PNGS variants (C) and combination PNGS variants (D) for glycosylated I53_dn5A pentamer and I53_dn5B trimer designs, detected using a mouse anti-myc tag primary mAb and a horse anti-mouse HRP-coupled secondary mAb. Numbers indicate the amino acid residue where an Asn was inserted. enh0, typical (non-enhanced) N-linked sequon; enh1, enhanced N-linked sequon. Glycosylated I53_dn5A and I53_dn5B variants carried forward for nanoparticle immunogen assembly and *in vivo* testing are indicated in green. L, molecular weight ladder.

(E) Schematic representations of glycosylated I53_dn5A and I53_dn5B showing the Asn insert locations on each protomer (left) and the glycosylated oligomers (right; glycans in green, I53_dn5A in orange, and I53_dn5B in gray; a single subunit of each oligomer shown as cartoon for detail).

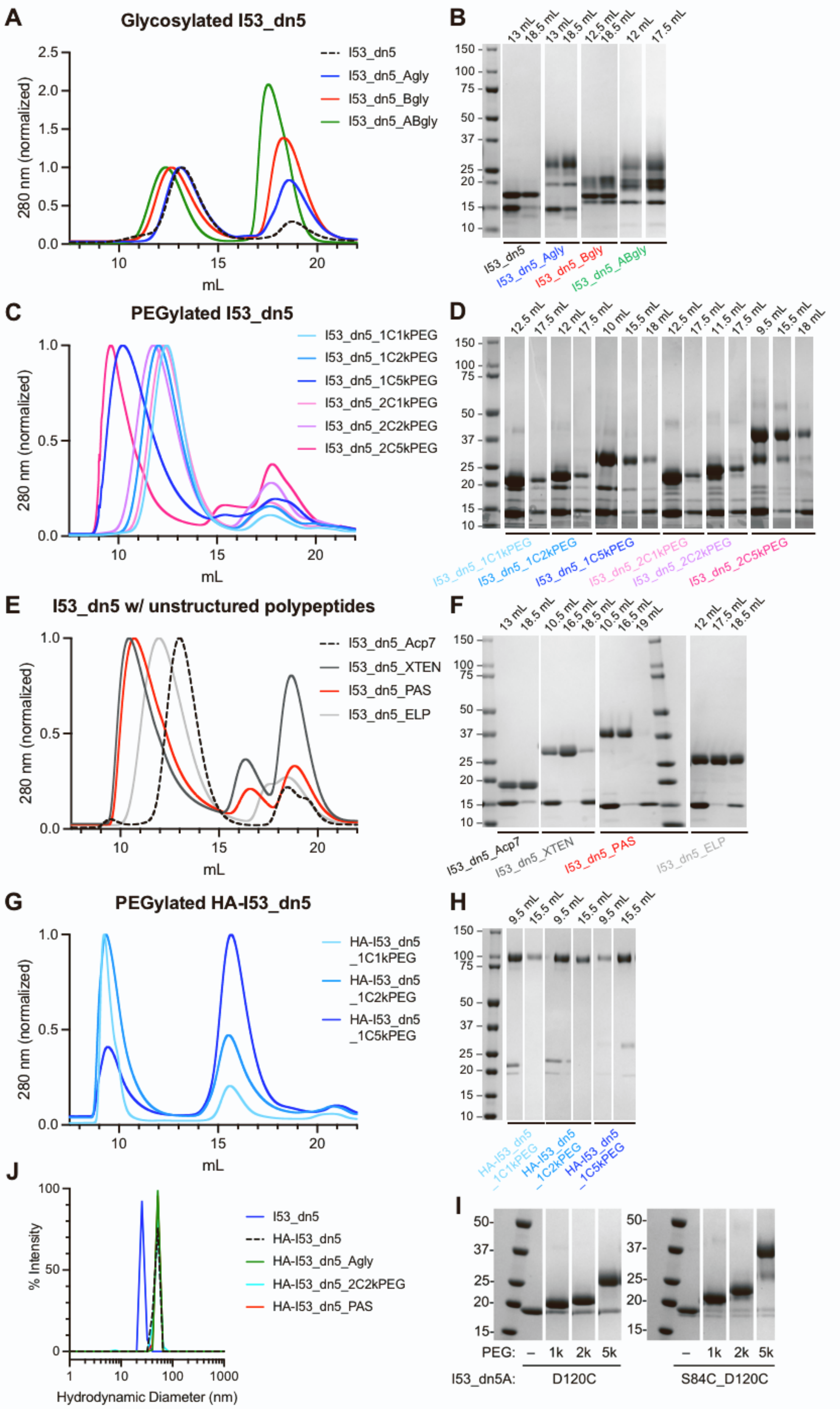
A
Glycosylated I53_dn5
280 nm (normalized)
mL
I53_dn5
I53_dn5_Agly
I53_dn5_Bgly
I53_dn5_ABgly
B
C
PEGylated I53_dn5
I53_dn5_1C1kPEG
I53_dn5_1C2kPEG
I53_dn5_1C5kPEG
I53_dn5_2C1kPEG
I53_dn5_2C2kPEG
I53_dn5_2C5kPEG
D
E
I53_dn5 w/ unstructured polypeptides
I53_dn5_Acp7
I53_dn5_XTEN
I53_dn5_PAS
I53_dn5_ELP
F
G
PEGylated HA-I53_dn5
HA-I53_dn5 _1C1kPEG
HA-I53_dn5 _1C2kPEG
HA-I53_dn5 _1C5kPEG
H
J
% Intensity
Hydrodynamic Diameter (nm)
HA-I53_dn5
HA-I53_dn5_Agly
HA-I53_dn5_2C2kPEG
HA-I53_dn5_PAS
I
PEG:
I53_dn5A:
D120C
S84C_D120C

**Figure S2. Characterization of Glycosylated, PEGylated, and PASylated I53_dn5 Nanoparticle Scaffolds, Related to Figure 1**

(A, C, and E) SEC purification of the I53_dn5 scaffold masked with glycans (A), PEG (C), or unstructured polypeptides (E) after *in vitro* assembly using a Superose 6 Increase 10/300 GL column. The nanoparticles elute at 9-15 mL and residual, unassembled components elute at larger volumes. In addition to the peak shifts being consistent with the molecular weight of the masking agent, in most cases modest effects on the *in vitro* assembly efficiency were also observed.

(B, D, and F) Reducing SDS-PAGE of SEC-purified I53_dn5 scaffold masked with glycans (D), PEG (D), or unstructured polypeptides (F) and residual, unassembled components. The presence of more unassembled components in the 18.5 mL peak for I53_dn5_Bgly compared to I53_dn5_Agly indicates that the I53_dn5B_2gly component has the lower nanoparticle assembly efficiency (A and B). Similarly, 5 kDa PEG, XTEN, and PAS polypeptides all have larger amounts of unassembled components in the 15-20 mL elution volumes compared to the smaller 1 and 2 kDa PEG and ELP polypeptide, indicating that these larger masking agents impeded nanoparticle assembly efficiency the most (C-F). From the SDS-PAGE presented in panel (D), we estimate PEG conjugation efficiency was >90% in all cases.

(G) SEC purification of PEGylated HA-I53_dn5 nanoparticle immunogens after *in vitro* assembly using a Superose 6 Increase 10/300 GL column. The nanoparticle immunogen elutes at the void volume. Residual, unassembled components elute around 15-18 mL. Note the declining *in vitro* assembly efficiency as the PEG molecular weight increases, suggesting larger PEG sterically hinders nanoparticle assembly when HA is fused to the I53_dn5B trimer.

(H) Reducing SDS-PAGE of SEC-purified PEGylated HA-I53_dn5 nanoparticle immunogens and residual, unassembled components. Only excess HA-I53_dn5B trimer was detected in the residual, unassembled component peak for both HA-I53_dn5_1C1kPEG and HA-I53_dn5_1C2kPEG immunogens, confirming complete nanoparticle assembly. However, both HA-I53_dn5B trimer and I53_dn5A_1C5kPEG pentamer were present in the 15.5 mL unassembled component peak for HA-I53_dn5_1C5kPEG immunogen, indicating that 5 kDa PEG on the I53_dn5A pentamer impeded efficient nanoparticle assembly.

(I) Reducing SDS-PAGE of I53_dn5A_D120C and I53_dn5A_S84C_D120C pentamers coupled to 1, 2, or 5 kDa PEG. Note the larger molecular weight shifts when PEG is coupled to I53_dn5A_S84C_D120C compared to I53_dn5A_D120C due to the presence of two unpaired cysteines (10 vs. 5 cysteines per pentamer, respectively). We estimate PEG conjugation efficiency was >90% in all cases.

(J) Dynamic light scattering of SEC-purified nanoparticle immunogens, including unmodified I53_dn5.

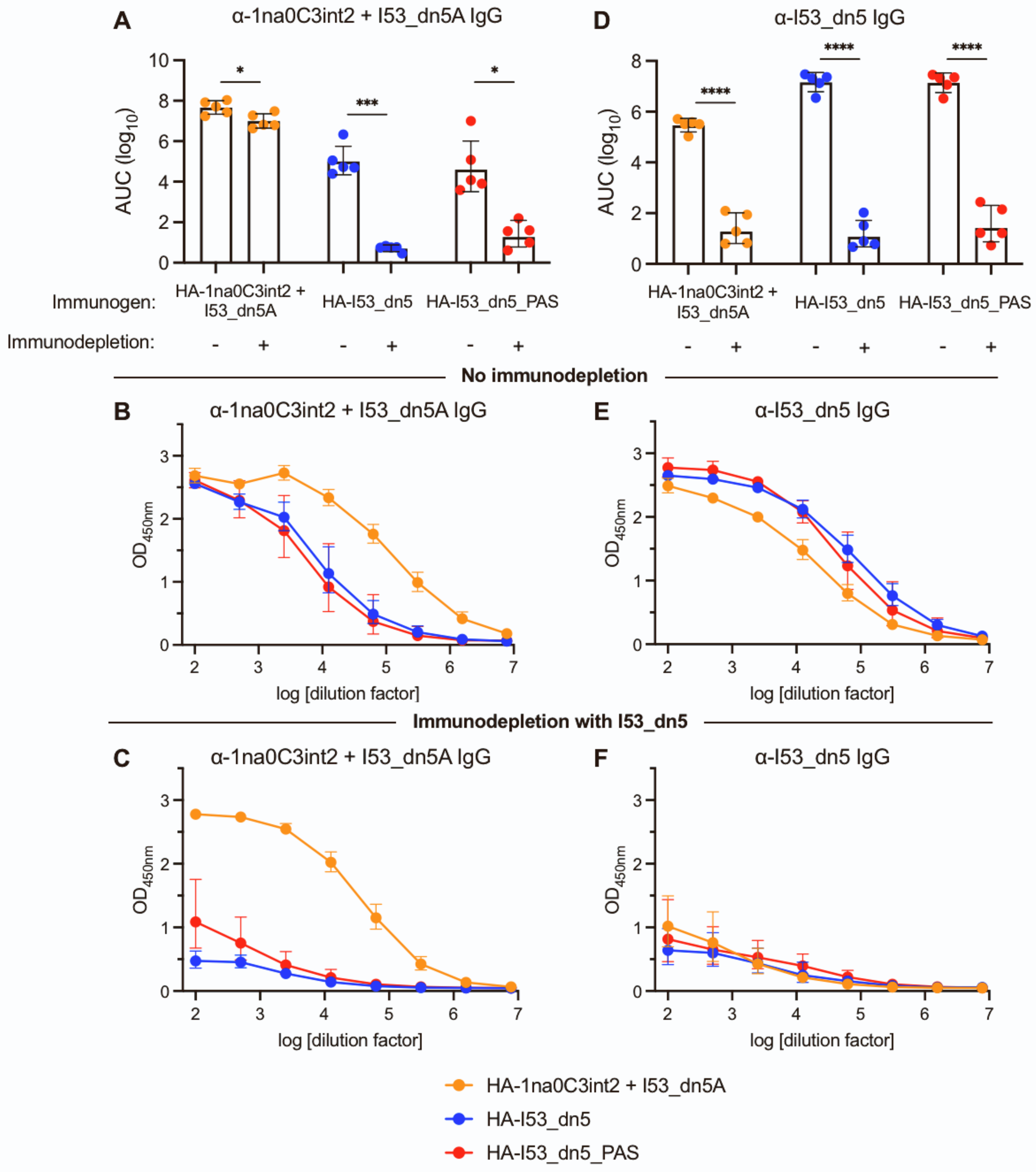

A
α-1na0C3int2 + I53_dn5A IgG
AUC (log10)
*
***
*
D
α-I53_dn5 IgG
****
****
****
Immunogen:
HA-1na0C3int2 + I53_dn5A
HA-I53_dn5
HA-I53_dn5_PAS
Immunodepletion:
- + - + - +
No immunodepletion
B
α-1na0C3int2 + I53_dn5A IgG
E
α-I53_dn5 IgG
OD450nm
log [dilution factor]
Immunodepletion with I53_dn5
C
α-1na0C3int2 + I53_dn5A IgG
F
α-I53_dn5 IgG
HA-1na0C3int2 + I53_dn5A
HA-I53_dn5
HA-I53_dn5_PAS

**Figure S3. HA-I53_dn5 and HA-I53_dn5_PAS Nanoparticle Immunogens Elicit Minimal Antibodies Against Epitopes on the Interior Surface of the Nanoparticle Scaffold, Related to Figure 2**

(A-F) Post-2nd boost (week 10) anti-unassembled HA-I53_dn5 nanoparticle components (HA-1na0C3int2 trimer + I53_dn5A pentamer) (A-C) and anti-I53_dn5 nanoparticle (D-F) serum IgG binding titers in BALB/c mice, measured by ELISA and plotted as the area under the curve (AUC) (A and D) for each serum dilution series (B, C, E, and F). Week 10 sera from mice immunized with either HA-1na0C3int2 + I53_dn5A unassembled components, HA-I53_dn5 nanoparticles, or HA-I53_dn5_PAS nanoparticles were analyzed for antibody titer against unassembled HA-I53_dn5 nanoparticle components (HA-1na0C3int2 trimer + I53_dn5A pentamer) (A-C) or I53_dn5 nanoparticles (D-F) before (B and E) or after (C and F) immunodepletion with I53_dn5. (A and D) Each symbol represents an individual animal and the geometric mean AUC and the geometric mean standard deviation from each group is indicated by the bar and error bar, respectively (N=5 mice/group).

*P* values between groups were determined by Brown-Forsythe and Welch one-way ANOVA test, with Dunnett's T3 multiple comparisons test. *$p < 0.05$; **$p < 0.01$; ***$p < 0.001$; ****$p < 0.0001$.

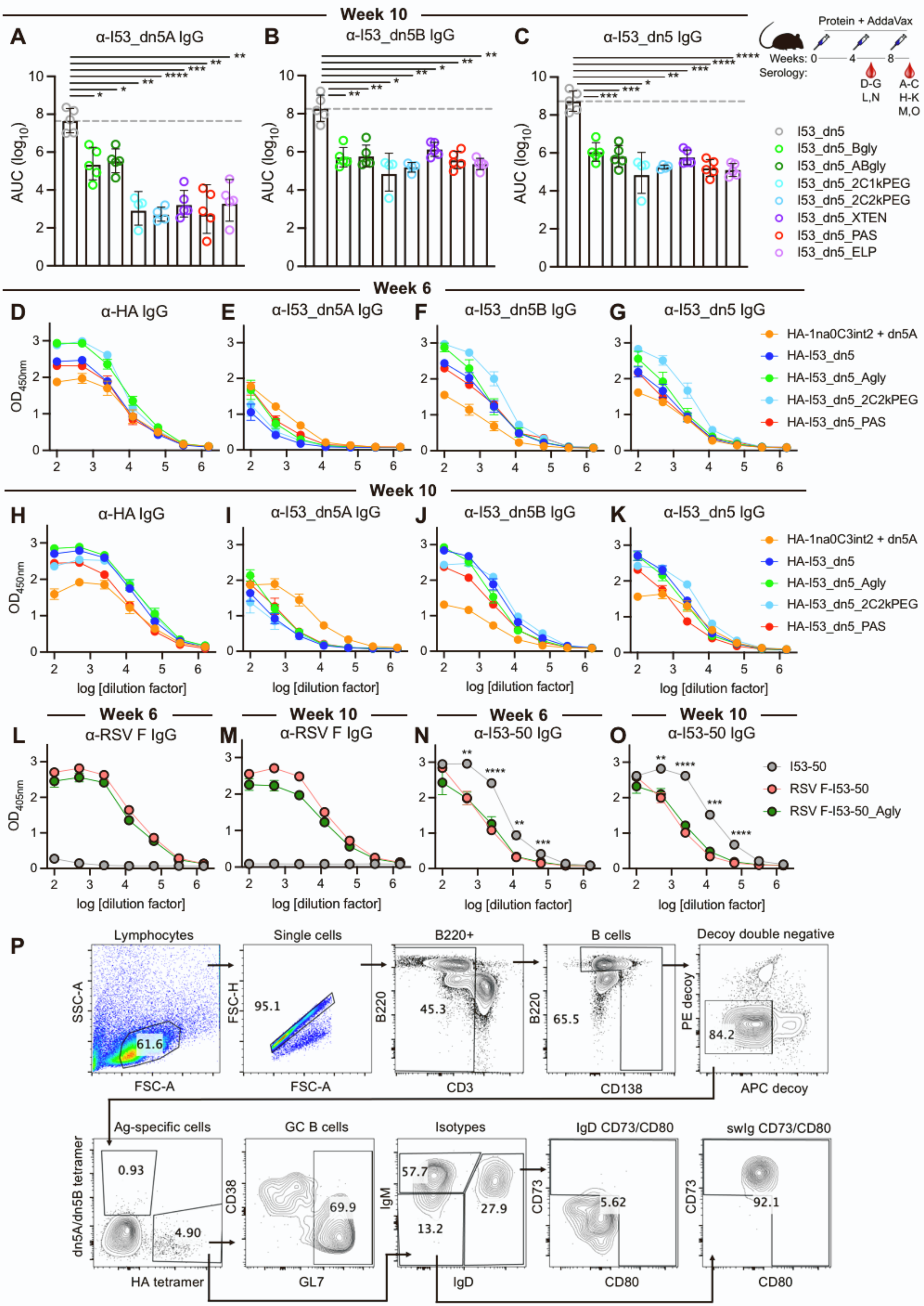

Week 10
A
α-I53_dn5A IgG
B
α-I53_dn5B IgG
C
α-I53_dn5 IgG
AUC (log10)
Protein + AddaVax
Weeks: 0
4
8
Serology:
D-G
L,N
A-C
H-K
M,O
I53_dn5
I53_dn5_Bgly
I53_dn5_ABgly
I53_dn5_2C1kPEG
I53_dn5_2C2kPEG
I53_dn5_XTEN
I53_dn5_PAS
I53_dn5_ELP
Week 6
D
α-HA IgG
E
α-I53_dn5A IgG
F
α-I53_dn5B IgG
G
α-I53_dn5 IgG
OD450nm
HA-1na0C3int2 + dn5A
HA-I53_dn5
HA-I53_dn5_Agly
HA-I53_dn5_2C2kPEG
HA-I53_dn5_PAS
Week 10
H
α-HA IgG
I
α-I53_dn5A IgG
J
α-I53_dn5B IgG
K
α-I53_dn5 IgG
log [dilution factor]
Week 6
L
α-RSV F IgG
Week 10
M
α-RSV F IgG
Week 6
N
α-I53-50 IgG
Week 10
O
α-I53-50 IgG
OD405nm
I53-50
RSV F-I53-50
RSV F-I53-50_Agly
P
Lymphocytes
SSC-A
FSC-A
61.6
Single cells
FSC-H
95.1
B220+
B220
CD3
45.3
B cells
CD138
65.5
Decoy double negative
PE decoy
APC decoy
84.2
Ag-specific cells
dn5A/dn5B tetramer
HA tetramer
0.93
4.90
GC B cells
CD38
GL7
69.9
Isotypes
IgM
IgD
57.7
27.9
13.2
IgD CD73/CD80
CD73
CD80
5.62
swIg CD73/CD80
92.1

**Figure S4. Masking the I53_dn5 Scaffold Reduces Scaffold-specific Antibody Responses when no Glycoprotein Antigen is Present, but Scaffold Masking does not Enhance Antigen-specific Responses when I53_dn5 and I53-50 Scaffolds Display a Glycoprotein Antigen, Related to Figure 2**

(A-C) Post-2$^{nd}$ boost (week 10) anti-I53_dn5A pentamer (A), anti-I53_dn5B trimer (B), and anti-I53_dn5 nanoparticle (C) serum IgG binding titers in BALB/c mice, measured by ELISA and plotted as the area under the curve (AUC) for each serum dilution series. Each symbol represents an individual animal and the geometric mean AUC and the geometric mean standard deviation from each group is indicated by the bar and error bar, respectively (N=5 mice/group). The gray dashed line represents levels for the unmodified I53_dn5 nanoparticle for comparison. The inset depicts the study timeline and the blood collection time point that each data panel represents. *P* values between groups were determined by Brown-Forsythe and Welch one-way ANOVA test, with Dunnett's T3 multiple comparisons test. *p < 0.05; **p < 0.01; ***p < 0.001; ****p < 0.0001.

(D-K) Post-1$^{st}$ boost (week 6) (D-G) and post-2$^{nd}$ boost (week 10) (H-K) anti-H1MI15 influenza hemagglutinin (D and H), anti-I53_dn5A pentamer (E and I), anti-I53_dn5B trimer (F and J), and anti-I53_dn5 nanoparticle (G and K) serum IgG ELISA curves in BALB/c mice. Each symbol represents the geometric mean absorbance at 450 nm +/- geometric mean SD (N=5 mice/group).

(L-O) Post-1$^{st}$ boost (week 6) (L and N) and post-2$^{nd}$ boost (week 10) (M and O) anti-DS-Cav1 RSV F protein (L and M) and anti-I53-50 nanoparticle (N and O) IgG ELISA curves in BALB/c mice. Each symbol represents the geometric mean absorbance at 450 nm +/- geometric mean SD (N=5 mice/group). *P* values between the 405 nm absorption values for I53-50 and RSV F-I53-50 at the indicated serum dilutions were determined by unpaired *t* tests. *p < 0.05; **p < 0.01; ***p < 0.001; ****p < 0.0001.

(P) Representative gating strategy for evaluating I53_dn5A-, I53_dn5B-, and HA-specific B cells, germinal center (GC) precursors and B cells ($CD38^{+/-}GL7^{+}$), and B cell isotypes. Top row, gating strategy for measuring numbers of live, non-doublet B cells. Bottom row, representative data from a mouse immunized with HA-I53_dn5 formulated with AddaVax. $HA^{+}CD38^{+/-}GL7^{+}$ cells that did not bind decoys were counted as antigen-specific GC precursors and B cells. GC precursors and B cells were further analyzed to characterize B cell receptor isotypes.

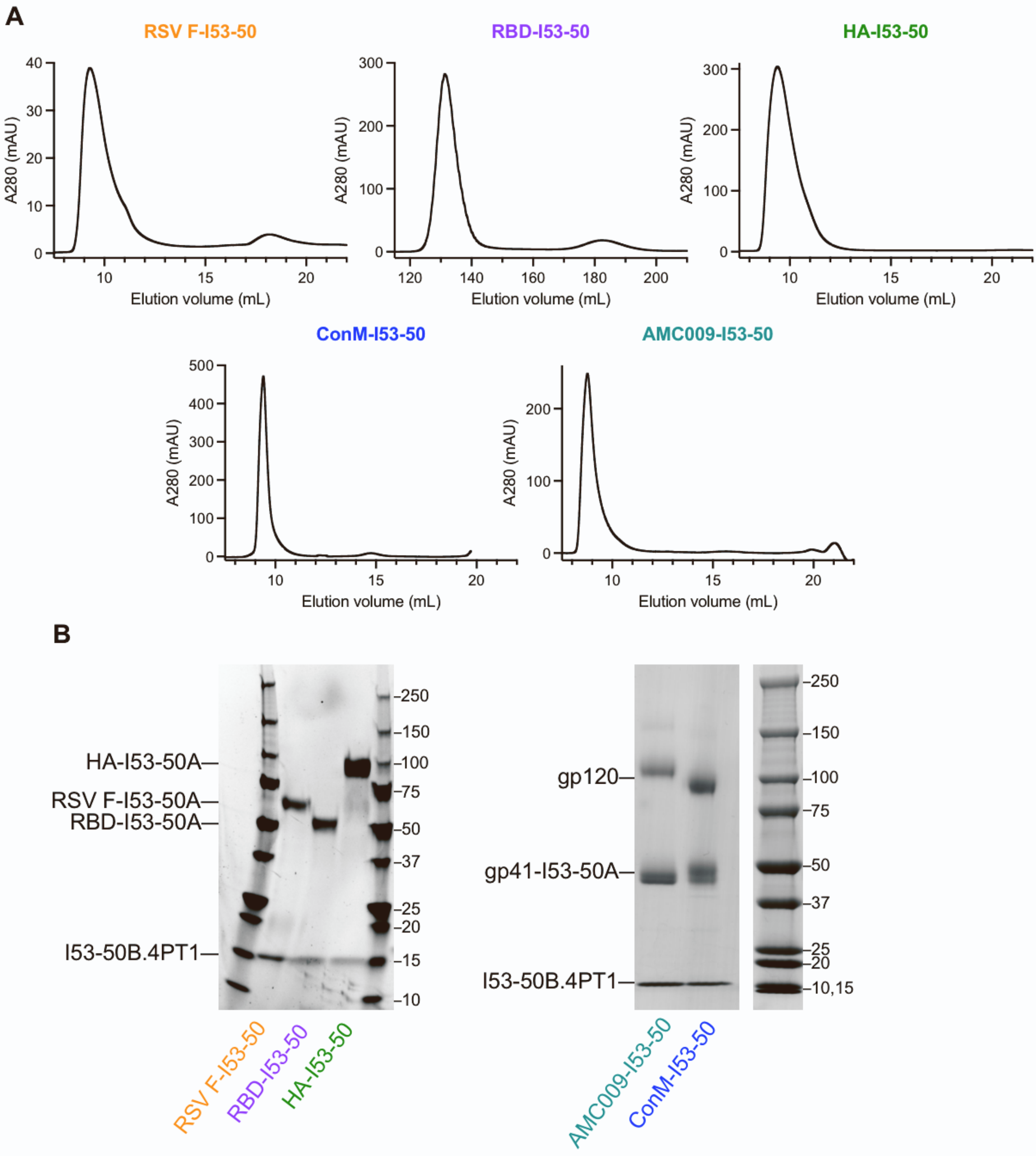


**Figure S5. SEC Purification and SDS-PAGE of I53-50-based Nanoparticle Immunogens, Related to Figure 3**
(A and B) SEC chromatograms from purification of the RSV F-I53-50, RBD-I53-50, HA-I53-50, ConM-I53-50, and AMC009-I53-50 nanoparticle immunogens after *in vitro* assembly using a HiLoad 26/600 Superdex 200 pg column for RBD-I53-50 and a Superose 6 Increase 10/300 GL column for the other nanoparticle immunogens (A), and SDS-PAGE of these nanoparticle immunogens after SEC purification (B).

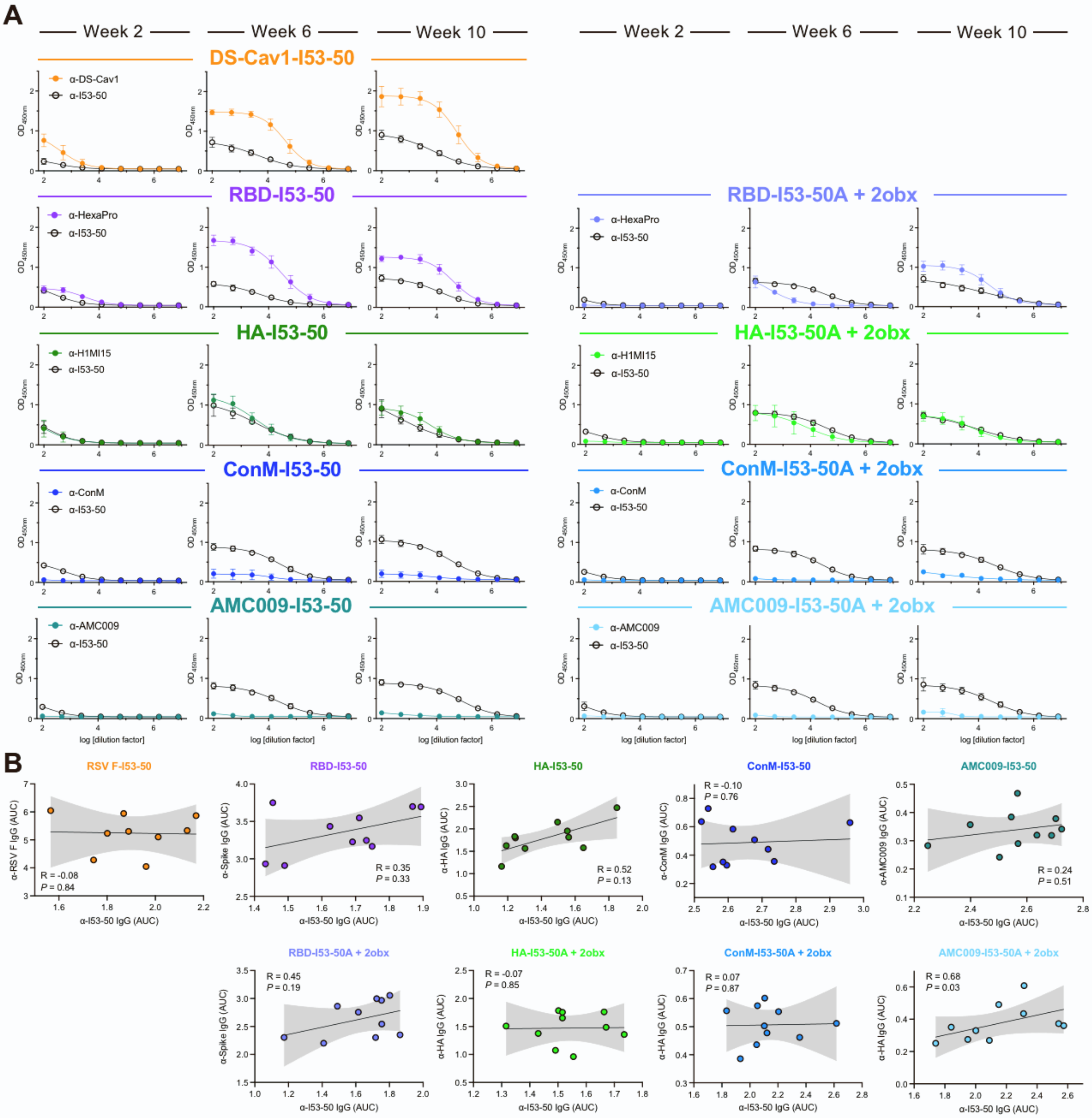


**Figure S6. Individual ELISA Curves for a Series of Different I53-50-based Nanoparticle Immunogens and Non-assembling Controls, Related to Figure 3**

(A) Post-prime (week 2), post-1st boost (week 6), and post-2nd boost (week 10) antigen-specific (solid colored circles) and anti-I53-50 nanoparticle (open black circles) serum IgG ELISA curves in BALB/c mice. Antigen-specific IgG titers were measured by Ni-NTA-capture ELISA. Each symbol represents the geometric mean absorbance at 450 nm +/- geometric mean SD (N=10 mice/group). Immunogen labels are listed at the top of their respective ELISA plots.

(B) Spearman's correlations between post-2nd boost (week 10) anti-antigen and anti-I53-50 scaffold serum IgG titers (AUC) for each individual immunogen. Shaded areas represent 95% confidence intervals of the plotted linear regression line. Each symbol represents a mouse (N=10 per immunogen).

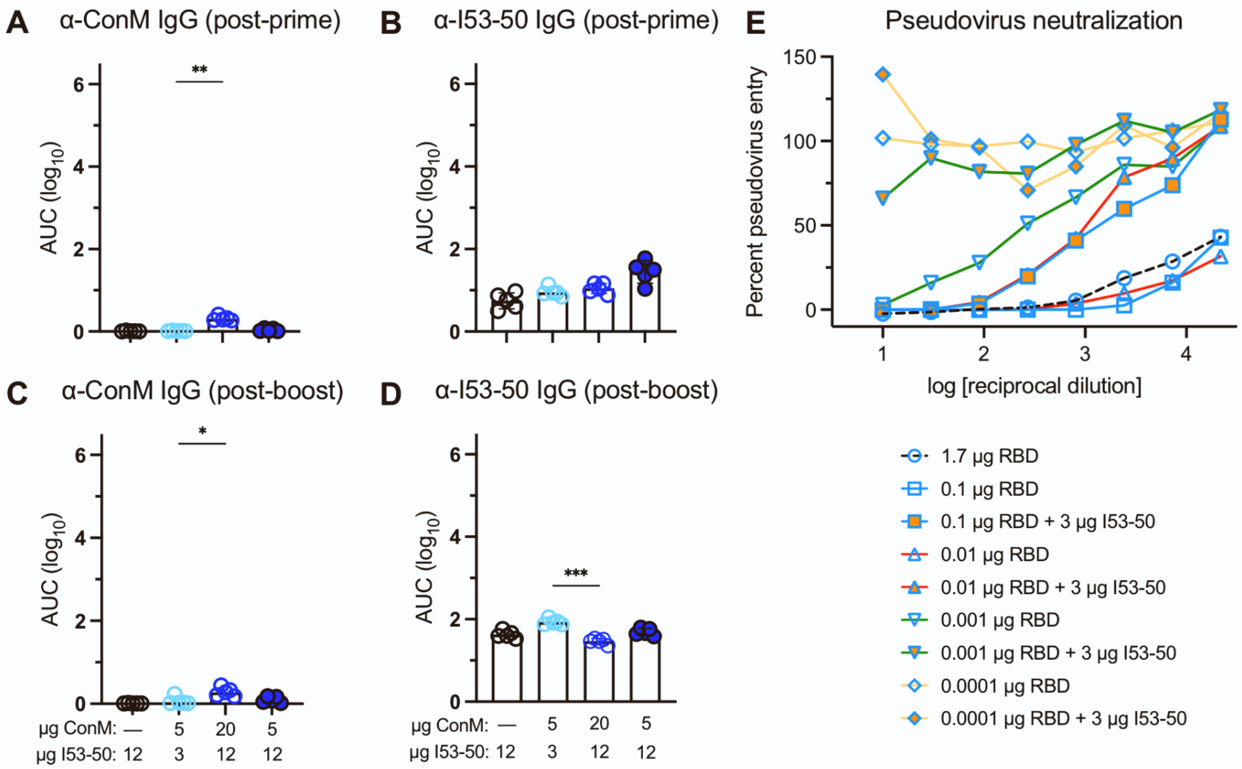


**Figure S7. Anti-ConM and Anti-I53-50 Antibody Responses at Different ConM-I53-50 Doses and with Co-delivered Excess I53-50 Scaffold, Related to Figure 5**

(A-D) Post-prime (week 2) (A and B) and post-boost (week 5) (C and D) anti-HIV-1 Env (ConM) (A and C) and anti-I53-50 scaffold (B and D) serum IgG binding titers in BALB/c mice immunized with the protein doses indicated at the bottom, measured by ELISA and plotted as the area under the curve (AUC) for each serum dilution series. Each symbol represents an individual animal and the geometric mean AUC from each group is indicated by the bar (N=5 mice/group). *P* values between groups were determined by Brown-Forsythe and Welch one-way ANOVA test, with Dunnett's T3 multiple comparisons test. *p < 0.05; **p < 0.01; ***p < 0.001; ****p < 0.0001.

(E) Percent pseudovirus entry curves used for calculating reciprocal $IC_{50}$ titer ($log_{10}$) values in Figure 5J. Each symbol represents the arithmetic mean of N=5 mice/group.

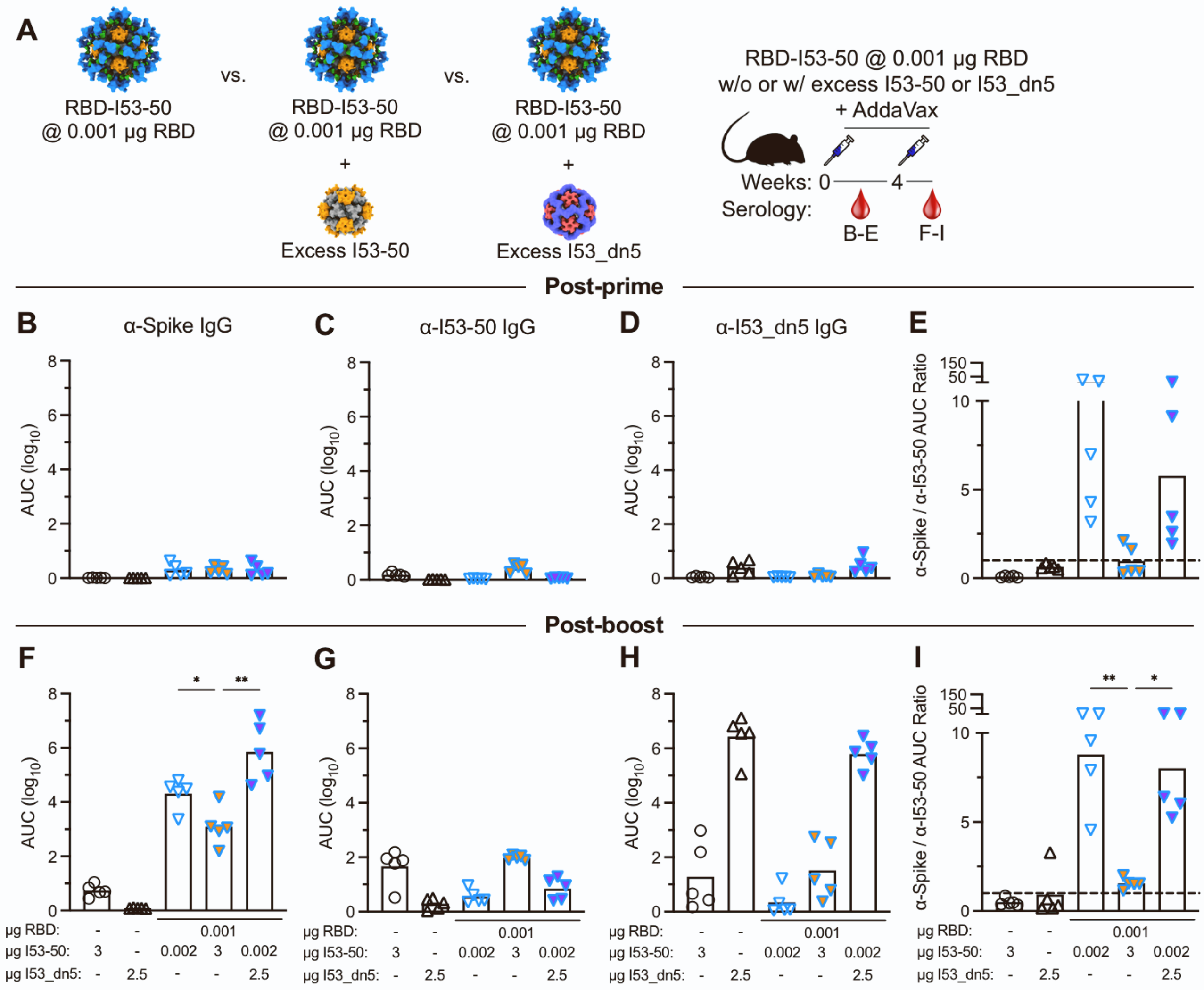


**Figure S8. Heterologous Scaffold-specific Antibody Responses do not Competitively Inhibit Antigen-specific Responses when Heterologous Scaffold is Provided in Excess, Related to Figure 5**

(A) (Left) Schematic representation of the homologous I53-50 and heterologous I53_dn5 scaffold competition experimental design where mice were immunized with the RBD-I53-50 nanoparticle immunogen at an RBD dose of 0.001 µg in the absence of excess scaffold, or co-delivered with excess homologous I53-50 or heterologous I53_dn5 nanoparticle scaffold. (Right) Schematic depicting the study timeline and blood collection time points that each data panel represents.

(B-I) Post-prime (week 2) (B-E) and post-boost (week 6) (F-I) anti-SARS-CoV-2 Spike (B and F), anti-I53-50 scaffold (C and G), and anti-I53_dn5 scaffold serum IgG binding titers in BALB/c mice immunized with the protein doses indicated at the bottom of F-I, measured by ELISA and plotted as the area under the curve (AUC) for each serum dilution series. Each symbol represents an individual animal and the geometric mean AUC from each group is indicated by the bar (N=5 mice/group).

(E and I) Ratio of the post-prime (week 2) (E) and post-boost (week 6) (I) Spike-specific to I53-50 scaffold-specific binding antibody titers (AUC). The black dashed line indicates a ratio of 1.

*P* values between groups were determined by Brown-Forsythe and Welch one-way ANOVA test, with Dunnett's T3 multiple comparisons test. *p < 0.05; **p < 0.01; ***p < 0.001; ****p < 0.0001.

## Table S1. Amino acid sequences for proteins used in this study, Related to STAR Methods

### Non-antigen-bearing nanoparticle components

>I53_dn5A pentamer
MGKYDGSKLRIGILHARWNAEIILALVLGALKRLQEFGVKRENIIIETVPGSFELPYGSKLFVEKQKRLGKPLDAIIPIGVLIKGSTMHFEY
ICDSTTHQLMKLNFELGIPVIFGVLTCLTDEQAEARAGLIEGKMHNHGEDWGAAAVEMATKFNLEHHHHHH

>I53_dn5A_1cys pentamer
MGKYDGSKLRIGILHARGNAEIILALVLGALKRLQEFGVKRENIIIETVPGSFELPYGSKLFVEKQKRLGKPLDAIIPIGVLIRGSTPHFDY
IADSTTHQLMKLNFELGIPVIFGVITADTCEQAEARAGLIEGKMHNHGEDWGAAAVEMATKFNGGWELQLEGSHHHHHH

>I53_dn5A_2cys pentamer
MGKYDGSKLRIGILHARGNAEIILALVLGALKRLQEFGVKRENIIIETVPGSFELPYGSKLFVEKQKRLGKPLDAIIPIGVLIRGCTPHFDY
IADSTTHQLMKLNFELGIPVIFGVITADTCEQAEARAGLIEGKMHNHGEDWGAAAVEMATKFNGGWELQLEGSHHHHHH

>I53_dn5Acp7_ELP1 pentamer
MGSHHHHHHGSDEQAEERAGTKAGNHGEDWGAAAVEMATKFNGSGGSGKYDGSKLRIGILHARGNAEIILELVLGALKRLQEFGVKRENIII
ETVPGSFELPYGSKLFVEKQKRLGKPLDAIIPIGVLIRGSTAHFDYIADSTTHQLMKLNFELGIPVIFGVLTTESGGSVPGAGVPGVGVPGV
GVPGAGVPGVGVPGAGVPGVGVPGAGVPGVGVPGVGVPGAGVPGVG

>I53_dn5Acp7_ELP2 pentamer
MGSHHHHHHGSDEQAEERAGTKAGNHGEDWGAAAVEMATKFNGSGGSGKYDGSKLRIGILHARGNAEIILELVLGALKRLQEFGVKRENIII
ETVPGSFELPYGSKLFVEKQKRLGKPLDAIIPIGVLIRGSTAHFDYIADSTTHQLMKLNFELGIPVIFGVLTTESGGSVPGAGVPGAGVPGA
GVPGAGVPGAGVPGAGVPGAGVPGAGVPGAGVPGAGVPGAGVPGAG

>I53_dn5Acp7_PAS pentamer
MGSHHHHHHGSDEQAEERAGTKAGNHGEDWGAAAVEMATKFNGSGGSGKYDGSKLRIGILHARGNAEIILELVLGALKRLQEFGVKRENIII
ETVPGSFELPYGSKLFVEKQKRLGKPLDAIIPIGVLIRGSTAHFDYIADSTTHQLMKLNFELGIPVIFGVLTTESGGSASPAAPAPASPAAP
APSAPAAASPAAPAPASPAAPAPSAPAAASPAAPAPASPAAPAPSAPAA

>I53_dn5A pentamer (I53_dn5A.2, optimized for mammalian cell secretion)
MDSKGSSQKGSRLLLLLVVSNLLLPQGVLAKYDGSKLRIGILHARGNAEIILELVLGALKRLQEFGVKRENIIIETVPGSFELPYGSKLFVE
KQKRLGKPLDAIIPIGVLIRGSTAHFDYIADSTTHQLMKLNFELGIPVIFGVLTTESDEQAEERAGTKAGNHGEDWGAAAVEMATKFNLEEQ
KLISEEDLHHHHHH

>I53_dn5A_2gly pentamer
MDSKGSSQKGSRLLLLLVVSNLLLPQGVLAKYDGSKLRIGILHARGNAEIILELVLGALKRLQEFGVKRENIIIETVPGSFELPYGSKLFVE
KQKRLGKPLDAIIPIGVLIRGNDTHFDYIADSTTHQLMKLNFELGIPVIFGVLTTNSTEQAEERAGTKAGNHGEDWGAAAVEMATKFNLEEQ
KLISEEDLHHHHHH

>I53_dn5B trimer
MEEAELAYLLGELAYKLGEYRIAIRAYRIALKRDPNNAEAWYNLGNAYYKQGRYREAIEYYQKALELDPNNAEAWYNLGNAYYERGEYEEAI
EYYRKALRLDPNNADAMQNLLNAKMREEGGWELQGSLEHHHHHH

>I53_dn5B_2gly trimer
MDSKGSSQKGSRLLLLLVVSNLLLPQGVLAEEAELAYLLGELAYKLGEYRIAIRAYRIALKYDNLTAEAWYNLGNAYYKQGRYREAIEYYQK
ALELDPNNAEAWYNLGNAYYERGEYENATEYYRKALRLDPNNADAMQNLLNAKMREELEEQKLISEEDLHHHHHH

>I53-50A trimer

MKMEELFKKHKIVAVLRANSVEEAIEKAVAVFAGGVHLIEITFTVPDADTVIKALSVLKEKGAIIGAGTVTSVEQCRKAVESGAEFIVSPHL
DEEISQFCKEKGVFYMPGVMTPTELVKAMKLGHTILKLFPGEVVGPQFVKAMKGPFPNVKFVPTGGVNLDNVCEWFKAGVLAVGVGSALVKG
TPDEVREKAKAFVEKIRGCLEEQKLISEEDLHHHHHH

>I53-50A_4gly trimer
MDSKGSSQKGSRLLLLLVVSNLLLPQGVLAEELFKKHKIVAVLRANSVEEAIEKAVAVFAGGVHLIEITFTVPNATTVIKALSVLKEKGAII
GAGTVTSVEYANETVESGAEFIVSPHLDEEISNFTKEKGVFYMPGVMTPTELVKAMKLGHTILKLFPGEVVGPQFVKAMKGPFHNATFVPTG
GVNLDNVCEWFKAGVLAVGVGSALVKGTPDEVREKAKAFVEKIRGCLEEQKLISEEDLHHHHHH

>I53-50B.4PT1 pentamer
MNQHSHKDHETVRIAVVRARWHAEIVDACVSAFEAAMRDIGGDRFAVDVFDVPGAYEIPLHARTLAETGR
YGAVLGTAFVVNGGIYRHEFVASAVINGMMNVQLNTGVPVLSAVLTPHNYDKSKAHTLLFLALFAVKGME
AARACVEILAAREKIAAGSLEHHHHHH

>2obx pentamer
MNQHSHKDYETVRIAVVRARWHADIVDQCVSAFEAEMADIGGDRFAVDVFDVPGAYEIPLHARTLAETGR
YGAVLGTAFVVNGGIYRHEFVASAVIDGMMNVQLSTGVPVLSAVLTPHNYHDSAEHHRFFFEHFTVKGKE
AARACVEILAAREKIAAGSLEHHHHHH

**Antigen-bearing nanoparticle components and ELISA antigens**

>H1MI15-I53_dn5B trimer (A/Michigan/45/2015 HA 1-676 Y98F no lkr dn5B.SA.WELQ-H)
MKAILVVLLYTFTTANADTLCIGYHANNSTDTVDTVLEKNVTVTHSVNLLEDKHNGKLCKLRGVAPLHLGKCNIAGWILGNPECESLSTASS
WSYIVETSNSDNGTCFPGDFINYEELREQLSSVSSFERFEIFPKTSSWPNHDSNKGVTAACPHAGAKSFYKNLIWLVKKGNSYPKLNQSYIN
DKGKEVLVLWGIHHPSTTADQQSLYQNADAYVFVGTSRYSKKFKPEIATRPKVRDQEGRMNYYWTLVEPGDKITFEATGNLVVPRYAFTMER
NAGSGIIISDTPVHDCNTTCQTPEGAINTSLPFQNIHPITIGKCPKYVKSTKLRLATGLRNVPSIQSRGLFGAIAGFIEGGWTGMVDGWYGY
HHQNEQGSGYAADLKSTQNAIDKITNKVNSVIEKMNTQFTAVGKEFNHLEKRIENLNKKVDDGFLDIWTYNAELLVLLENERTLDYHDSNVK
NLYEKVRNQLKNNAKEIGNGCFEFYHKCDNTCMESVKNGTYDYPKYSEEAKLNREKIDGVSAEEAELAYLLGELAYKLGEYRIAIRAYRIAL
KRDPNNAEAWYNLGNAYYKQGRYREAIEYYQKALELDPNNAEAWYNLGNAYYERGEYEEAIEYYRKALRLDPNNADAMQNLLNAKMREEGGW
ELQHHHHHH

>H1MI15-I53-50A trimer (A/Michigan/45/2015 HA 1-676 Y98F)
MDSKGSSQKGSRLLLLLVVSNLLLPQGVLADTLCIGYHANNSTDTVDTVLEKNVTVTHSVNLLEDKHNGKLCKLRGVAPLHLGKCNIAGWIL
GNPECESLSTASSWSYIVETSNSDNGTCFPGDFINYEELREQLSSVSSFERFEIFPKTSSWPNHDSNKGVTAACPHAGAKSFYKNLIWLVKK
GNSYPKLNQSYINDKGKEVLVLWGIHHPSTTADQQSLYQNADAYVFVGTSRYSKKFKPEIATRPKVRDQEGRMNYYWTLVEPGDKITFEATG
NLVVPRYAFTMERNAGSGIIISDTPVHDCNTTCQTPEGAINTSLPFQNIHPITIGKCPKYVKSTKLRLATGLRNVPSIQSRGLFGAIAGFIE
GGWTGMVDGWYGYHHQNEQGSGYAADLKSTQNAIDKITNKVNSVIEKMNTQFTAVGKEFNHLEKRIENLNKKVDDGFLDIWTYNAELLVLLE
NERTLDYHDSNVKNLYEKVRNQLKNNAKEIGNGCFEFYHKCDNTCMESVKNGTYDYPKYSEEAKLNREKIDGVSAGSGGSGGSGGSGGSEKA
AKAEEEAARKMEELFKKHKIVAVLRANSVEEAIEKAVAVFAGGVHLIEITFTVPDADTVIKALSVLKEKGAIIGAGTVTSVEQCRKAVESGAE
FIVSPHLDEEISQFCKEKGVFYMPGVMTPTELVKAMKLGHTILKLFPGEVVGPQFVKAMKGPFPNVKFVPTGGVNLDNVCEWFKAGVLAVGV
GSALVKGTPDEVREKAKAFVEKIRGCTELEEQKLISEEDLHHHHHH

>H1MI15-foldon trimer (A/Michigan/45/2015 HA 1-676 Y98F FAH)
MDSKGSSQKGSRLLLLLVVSNLLLPQGVLADTLCIGYHANNSTDTVDTVLEKNVTVTHSVNLLEDKHNGKLCKLRGVAPLHLGKCNIAGWIL
GNPECESLSTASSWSYIVETSNSDNGTCFPGDFINYEELREQLSSVSSFERFEIFPKTSSWPNHDSNKGVTAACPHAGAKSFYKNLIWLVKK
GNSYPKLNQSYINDKGKEVLVLWGIHHPSTTADQQSLYQNADAYVFVGTSRYSKKFKPEIATRPKVRDQEGRMNYYWTLVEPGDKITFEATG
NLVVPRYAFTMERNAGSGIIISDTPVHDCNTTCQTPEGAINTSLPFQNIHPITIGKCPKYVKSTKLRLATGLRNVPSIQSRGLFGAIAGFIE
GGWTGMVDGWYGYHHQNEQGSGYAADLKSTQNAIDKITNKVNSVIEKMNTQFTAVGKEFNHLEKRIENLNKKVDDGFLDIWTYNAELLVLLE
NERTLDYHDSNVKNLYEKVRNQLKNNAKEIGNGCFEFYHKCDNTCMESVKNGTYDYPKYSEEAKLNREKIDGVGSGYIPEAPRDGQAYVRKD
GEWVLLSTFLGSGLNDIFEAQKIEWHEGHHHHHH

>H1MI15-1na0C3_int2 trimer (A/Michigan/45/2015 HA 1-676 Y98F no lkr 1na0C3_int2.SA.WELQ-H)
MKAILVVLLYTFTTANADTLCIGYHANNSTDTVDTVLEKNVTVTHSVNLLEDKHNGKLCKLRGVAPLHLGKCNIAGWILGNPECESLSTASS
WSYIVETSNSDNGTCFPGDFINYEELREQLSSVSSFERFEIFPKTSSWPNHDSNKGVTAACPHAGAKSFYKNLIWLVKKGNSYPKLNQSYIN
DKGKEVLVLWGIHHPSTTADQQSLYQNADAYVFVGTSRYSKKFKPEIATRPKVRDQEGRMNYYWTLVEPGDKITFEATGNLVVPRYAFTMER
NAGSGIIISDTPVHDCNTTCQTPEGAINTSLPFQNIHPITIGKCPKYVKSTKLRLATGLRNVPSIQSRGLFGAIAGFIEGGWTGMVDGWYGY
HHQNEQGSGYAADLKSTQNAIDKITNKVNSVIEKMNTQFTAVGKEFNHLEKRIENLNKKVDDGFLDIWTYNAELLVLLENERTLDYHDSNVK
NLYEKVRNQLKNNAKEIGNGCFEFYHKCDNTCMESVKNGTYDYPKYSEEAKLNREKIDGVSAEEAELAYLLGELAYKLGEYRIAIRAYRIAL
KRDPNNAEAWYNLGNAYYKQGDYDEAIEYYQKALELDPNNAEAWYNLGNAYYKQGDYDEAIEYYQKALELDPNNAEAKQNLGNAKQKQGGGW
ELQHHHHHH

>SARS-CoV-2_RBD-I53-50A trimer (16GS linker, using wild type RBD from Wuhan-Hu-1)
MGILPSPGMPALLSLVSLLSVLLMGCVAETGTRFPNITNLCPFGEVFNATRFASVYAWNRKRISNCVADYSVLYNSASFSTFKCYGVSPTKL
NDLCFTNVYADSFVIRGDEVRQIAPGQTGKIADYNYKLPDDFTGCVIAWNSNNLDSKVGGNYNYLYRLFRKSNLKPFERDISTEIYQAGSTP
CNGVEGFNCYFPLQSYGFQPTNGVGYQPYRVVVLSFELLHAPATVCGPKKSTGGSGGSGSGGSGGSGSEKAAKAEEAARKMEELFKKHKIVA
VLRANSVEEAIEKAVAVFAGGVHLIEITFTVPDADTVIKALSVLKEKGAIIGAGTVTSVEQARKAVESGAEFIVSPHLDEEISQFAKEKGVF
YMPGVMTPTELVKAMKLGHTILKLFPGEVVGPQFVKAMKGPFPNVKFVPTGGVNLDNVAEWFKAGVLAVGVGSALVKGTPDEVREKAKAFVE
KIRGATEGGSHHHHHHHH

>SARS-CoV-2_SpikeHexaPro-foldon trimer
MFVFLVLLPLVSSQCVNLTTRTQLPPAYTNSFTRGVYYPDKVFRSSVLHSTQDLFLPFFSNVTWFHAIHVSGTNGTKRFDNPVLPFNDGVYF
ASTEKSNIIRGWIFGTTLDSKTQSLLIVNNATNVVIKVCEFQFCNDPFLGVYYHKNNKSWMESEFRVYSSANNCTFEYVSQPFLMDLEGKQG
NFKNLREFVFKNIDGYFKIYSKHTPINLVRDLPQGFSALEPLVDLPIGINITRFQTLLALHRSYLTPGDSSSGWTAGAAAYYVGYLQPRTFL
LKYNENGTITDAVDCALDPLSETKCTLKSFTVEKGIYQTSNFRVQPTESIVRFPNITNLCPFGEVFNATRFASVYAWNRKRISNCVADYSVL
YNSASFSTFKCYGVSPTKLNDLCFTNVYADSFVIRGDEVRQIAPGQTGKIADYNYKLPDDFTGCVIAWNSNNLDSKVGGNYNYLYRLFRKSN
LKPFERDISTEIYQAGSTPCNGVEGFNCYFPLQSYGFQPTNGVGYQPYRVVVLSFELLHAPATVCGPKKSTNLVKNKCVNFNFNGLTGTGVL
TESNKKFLPFQQFGRDIADTTDAVRDPQTLEILDITPCSFGGVSVITPGTNTSNQVAVLYQDVNCTEVPVAIHADQLTPTWRVYSTGSNVFQ
TRAGCLIGAEHVNNSYECDIPIGAGICASYQTQTNSPGSASSVASQSIIAYTMSLGAENSVAYSNNSIAIPTNFTISVTTEILPVSMTKTSV
DCTMYICGDSTECSNLLLQYGSFCTQLNRALTGIAVEQDKNTQEVFAQVKQIYKTPPIKDFGGFNFSQILPDPSKPSKRSPIEDLLFNKVTL
ADAGFIKQYGDCLGDIAARDLICAQKFNGLTVLPPLLTDEMIAQYTSALLAGTITSGWTFGAGPALQIPFPMQMAYRFNGIGVTQNVLYENQ
KLIANQFNSAIGKIQDSLSSTPSALGKLQDVVNQNAQALNTLVKQLSSNFGAISSVLNDILSRLDPPEAEVQIDRLITGRLQSLQTYVTQQL
IRAAEIRASANLAATKMSECVLGQSKRVDFCGKGYHLMSFPQSAPHGVVFLHVTYVPAQEKNFTTAPAICHDGKAHFPREGVFVSNGTHWFV
TQRNFYEPQIITTDNTFVSGNCDVVIGIVNNTVYDPLQPELDSFKEELDKYFKNHTSPDVDLGDISGINASVVNIQKEIDRLNEVAKNLNES
LIDLQELGKYEQGSGYIPEAPRDGQAYVRKDGEWVLLSTFLGRSLEVLFQGPGHHHHHHHHSAWSHPQFEKGGGSGGGGSGGSAWSHPQFEK

>RSV-F_DS-Cav1-I53-50A trimer
MELLILKANAITTILTAVTFCFASGQNITEEFYQSTCSAVSKGYLSALRTGWYTSVITIELSNIKENKCNGTDAKVKLIKQELDKYKNAVTE
LQLLMQSTPATNNRARRELPRFMNYTLNNAKKTNVTLSKKRKRRFLGFLLGVGSAIASGVAVCKVLHLEGEVNKIKSALLSTNKAVVSLSNG
VSVLTFKVLDLKNYIDKQLLPILNKQSCSISNIETVIEFQQKNNRLLEITREFSVNAGVTTPVSTYMLTNSELLSLINDMPITNDQKKLMSN
NVQIVRQQSYSIMCIIKEEVLAYVVQLPLYGVIDTPCWKLHTSPLCTTNTKEGSNICLTRTDRGWYCDNAGSVSFFPQAETCKVQSNRVFCD
TMNSLTLPSEVNLCNVDIFNPKYDCKIMTSKTDVSSSVITSLGAIVSCYGKTKCTASNKNRGIIKTFSNGCDYVSNKGVDTVSVGNTLYYVN
KQEGKSLYVKGEPIINFYDPLVFPSDEFDASISQVNEKINQSLAFIRKSDELLGSGGSGSGSGGSEKAAKAEEAARKMEELFKKHKIVAVLR
ANSVEEAIEKAVAVFAGGVHLIEITFTVPDADTVIKALSVLKEKGAIIGAGTVTSVEQCRKAVESGAEFIVSPHLDEEISQFCKEKGVFYMP
GVMTPTELVKAMKLGHTILKLFPGEVVGPQFVKAMKGPFPNVKFVPTGGVNLDNVCEWFKAGVLAVGVGSALVKGTPDEVREKAKAFVEKIR
GCTELEHHHHHH

>RSV-F_DS-Cav1-foldon trimer
MELLILKANAITTILTAVTFCFASGQNITEEFYQSTCSAVSKGYLSALRTGWYTSVITIELSNIKENKCNGTDAKVKLIKQELDKYKNAVTE
LQLLMQSTPATNNRARRELPRFMNYTLNNAKKTNVTLSKKRKRRFLGFLLGVGSAIASGVAVCKVLHLEGEVNKIKSALLSTNKAVVSLSNG
VSVLTFKVLDLKNYIDKQLLPILNKQSCSISNIETVIEFQQKNNRLLEITREFSVNAGVTTPVSTYMLTNSELLSLINDMPITNDQKKLMSN
NVQIVRQQSYSIMCIIKEEVLAYVVQLPLYGVIDTPCWKLHTSPLCTTNTKEGSNICLTRTDRGWYCDNAGSVSFFPQAETCKVQSNRVFCD
TMNSLTLPSEVNLCNVDIFNPKYDCKIMTSKTDVSSSVITSLGAIVSCYGKTKCTASNKNRGIIKTFSNGCDYVSNKGVDTVSVGNTLYYVN

KQEGKSLYVKGEPIINFYDPLVFPSDEFDASISQVNEKINQSLAFIRKSDELLSAIGGYIPEAPRDGQAYVRKDGEWVLLSTFLENLYFQSS
AWSHPQFEKGGGSGGGSGGSAWSHPQFEKGSGSGSGLNDIFEAQKIEWHEGSGSGSHHHHHHHH

>HIVenv(AMC009)-I53-50A trimer
ADKLWVTVYYGVPVWKDACTTLFCASDAKAYDTEKRNVWATHCCVPTDPNPQEVVLENVTENFNMWKNDMVEQMHEDIISLWDQSLKPCVKL
TPLCVTLNCTDYVGNATNASTTNATGGIGGTVERGEIKNCSFNITTSLRDKVQKEYALFYKLDIVPIDNDNTNNTYRLINCNTTVIKQACPK
VSFEPIPIHYCAPAGFAILKCNDKKFNGTGPCTNVSTVQCTHGIRPVVSTQLLLNGSLAEKEVIIRSQNFTNNAKVIIVQLNESVVINCTRP
NNNTVKSIHIAPGQWFYYTGAIIGDIRQAHCNISRVKWNNTLKQIATKLREQFKNKTIAFNQSSGGDPEIVMHSFNCGGEFFYCNTTQLFNS
TWNDTEVSNYTDITHITLPCRIKQIINMWQRVGQAMYAPPIRGQIRCSSNITGLLLTRDGGSNENKTSETETFRPAGGDMRDNWRSELYKYK
VVKIEPLGVAPTRCKRRVVQRRRRRRAVGAIGAVSLGFLGAAGSTMGAASMTLTVQARQLLSGIVQQQNNCLRAPECQQHMLKDTHWGIKQL
QARVLAVEHYLRDQQLLGIWGCSGKLICCTAVPWNNTWSNRSLDMIWNNMTWIEWEREIDNYTGLIYNLLEESQNQQEKNEQELLELDGSGG
SGGSGGSGGSEKAAKAEEAARKMEELFKKHKIVAVLRANSVEEAIEKAVAVFAGGVHLIEITFTVPDADTVIKALSVLKEKGAIIGAGTVTS
VEQCRKAVESGAEFIVSPHLDEEISQFCKEKGVFYMPGVMTPTELVKAMKLGHTILKLFPGEVVGPQFVKAMKGPFPNVKFVPTGGVNLDNV
CEWFKAGVLAVGVGSALVKGTPDEVREKAKAFVEKIRGCTE

>HIVenv(ConM)-I53-50A trimer
AENLWVTVYYGVPVWKDAETTLFCASDAKAYDTEKRNVWATHCCVPTDPNPQEIVLENVTENFNMWKNNMVEQMHTDIISLWDQSLKPCVKL
TPLCVTLNCTDVNATNNTTNNEEIKNCSFNITTELRDKKKKVYALFYKLDVVPIDDNNSYRLINCNTSAITQACPKVSFEPIPIHYCAPAGF
AILKCNDKKFNGTGPCKNVSTVQCTHGIKPVVSTQLLLNGSLAEEEIIIRSENITNNAKTIIVQLNESVEINCTRPNNNTRKSIRIGPGQWF
YATGDIIGDIRQAHCNISRTKWNKTLQQVAKKLREHFNKTIIFNPSSGGDLEITTHSFNCGGEFFYCNTSELFNSTWNGTNNTITLPCRIKQ
IINMWQRVGQAMYAPPIEGKIRCTSNITGLLLTRDGGNNNTETFRPGGGDMRDNWRSELYKYKVVKIEPLGVAPTRCKRRVVERRRRRRAVG
IGAVFLGFLGAAGSTMGAASMTLTVQARNLLSGIVQQQSNLLRAPECQQHLLQLTVWGIKQLQARVLAVERYLKDQQLLGIWGCSGKLICCT
NVPWNSSWSNKSQDEIWDNMTWMEWDKEINNYTDIIYSLIEESQNQQEKNEQELLALDGSGGSGGSGGSGGSEKAAKAEEAARKMEELFKKH
KIVAVLRANSVEEAIEKAVAVFAGGVHLIEITFTVPDADTVIKALSVLKEKGAIIGAGTVTSVEQCRKAVESGAEFIVSPHLDEEISQFCKE
KGVFYMPGVMTPTELVKAMKLGHTILKLFPGEVVGPQFVKAMKGPFPNVKFVPTGGVNLDNVCEWFKAGVLAVGVGSALVKGTPDEVREKAK
AFVEKIRGCTE

>HIVenv(AMC009)-8xHis trimer
ADKLWVTVYYGVPVWKDACTTLFCASDAKAYDTEKRNVWATHCCVPTDPNPQEVVLENVTENFNMWKNDMVEQMHEDIISLWDQSLKPCVKL
TPLCVTLNCTDYVGNATNASTTNATGGIGGTVERGEIKNCSFNITTSLRDKVQKEYALFYKLDIVPIDNDNTNNTYRLINCNTTVIKQACPK
VSFEPIPIHYCAPAGFAILKCNDKKFNGTGPCTNVSTVQCTHGIRPVVSTQLLLNGSLAEKEVIIRSQNFTNNAKVIIVQLNESVVINCTRP
NNNTVKSIHIAPGQWFYYTGAIIGDIRQAHCNISRVKWNNTLKQIATKLREQFKNKTIAFNQSSGGDPEIVMHSFNCGGEFFYCNTTQLFNS
TWNDTEVSNYTDITHITLPCRIKQIINMWQRVGQAMYAPPIRGQIRCSSNITGLLLTRDGGSNENKTSETETFRPAGGDMRDNWRSELYKYK
VVKIEPLGVAPTRCKRRVVQRRRRRRAVGAIGAVSLGFLGAAGSTMGAASMTLTVQARQLLSGIVQQQNNCLRAPECQQHMLKDTHWGIKQL
QARVLAVEHYLRDQQLLGIWGCSGKLICCTAVPWNNTWSNRSLDMIWNNMTWIEWEREIDNYTGLIYNLLEESQNQQEKNEQELLELDGSGS
GGSGHHHHHHHH

>HIVenv(ConM)-8xHis trimer
AENLWVTVYYGVPVWKDAETTLFCASDAKAYDTEKRNVWATHCCVPTDPNPQEIVLENVTENFNMWKNNMVEQMHTDIISLWDQSLKPCVKL
TPLCVTLNCTDVNATNNTTNNEEIKNCSFNITTELRDKKKKVYALFYKLDVVPIDDNNSYRLINCNTSAITQACPKVSFEPIPIHYCAPAGF
AILKCNDKKFNGTGPCKNVSTVQCTHGIKPVVSTQLLLNGSLAEEEIIIRSENITNNAKTIIVQLNESVEINCTRPNNNTRKSIRIGPGQWF
YATGDIIGDIRQAHCNISRTKWNKTLQQVAKKLREHFNKTIIFNPSSGGDLEITTHSFNCGGEFFYCNTSELFNSTWNGTNNTITLPCRIKQ
IINMWQRVGQAMYAPPIEGKIRCTSNITGLLLTRDGGNNNTETFRPGGGDMRDNWRSELYKYKVVKIEPLGVAPTRCKRRVVERRRRRRAVG
IGAVFLGFLGAAGSTMGAASMTLTVQARNLLSGIVQQQSNLLRAPECQQHLLQLTVWGIKQLQARVLAVERYLKDQQLLGIWGCSGKLICCT
NVPWNSSWSNKSQDEIWDNMTWMEWDKEINNYTDIIYSLIEESQNQQEKNEQELLALDGSGSGGSGHHHHHHHH

## Data S1. Rosetta XML code for protein glycosylation, Related to STAR Methods

```
<ROSETTASCRIPTS>
  <SCOREFXNS>
       <ScoreFunction name="sfx_clean" weights="beta" symmetric="0" /> //function to obtain a score
  </SCOREFXNS>
```

```
  <RESIDUE_SELECTORS>
       <Index name="select_i_enh0" resnums="%%resi_enh0%%" /> //select the residue(s) to glycosylate (these
residues are a "non-enhanced" sequon)
              Not name="not_resis" selector="select" /> //all other residues not selected
       <Index name="select_i_enh1" resnums="%%resi_enh1%%" /> //select the residue(s) to glycosylate (these
residues are an "enhanced" sequon)
              Not name="not_resis" selector="select" /> //all other residues not selected
       Index name="select_i-2" resnums="%%enhresi%%" /> //select the residue i-2 from the N-linked
glycosylation site
       Index name="select_i_score" resnums="%%iresi%%" /> //select Asn residue i that is N-liked
glycosylated to get its score
  </RESIDUE_SELECTORS>
  <FILTERS>
       EnergyPerResidue name="total_energy_per_res_filter_i" scorefxn="sfx_clean" energy_cutoff="10000"
resnums="%%iresi%%" /> // tests the energy of a particular residue, or interface, or whole protein, or a set
of residues; energy must be less than 10000
       EnergyPerResidue name="total_energy_per_res_filter_i-2" scorefxn="sfx_clean" energy_cutoff="10000"
resnums="%%enhresi%%" /> // tests the energy of a particular residue, or interface, or whole protein, or a
set of residues; energy must be less than 10000
       EnergyPerResidue name="fa_atr_per_res_filter" scorefxn="sfx_clean" score_type="fa_atr"
energy_cutoff="10000" resnums="%%resi%%" />
       EnergyPerResidue name="fa_rep_per_res_filter" scorefxn="sfx_clean" score_type="fa_rep"
energy_cutoff="10000" resnums="%%resi%%" />
       EnergyPerResidue name="fa_dun_per_res_filter" scorefxn="sfx_clean" score_type="fa_dun"
energy_cutoff="10000" resnums="%%resi%%" />
       EnergyPerResidue name="fa_elec_per_res_filter" scorefxn="sfx_clean" score_type="fa_elec"
energy_cutoff="10000" resnums="%%resi%%" />
  </FILTERS>

  <MOVERS>
       <CreateGlycanSequonMover name="create_motif_enh0" residue_selector="select_i_enh0"
basic_enhanced_n_sequon="0" design_x_positions="1" pack_neighbors="1" scorefxn="sfx_clean" />
       <CreateGlycanSequonMover name="create_motif_enh1" residue_selector="select_i_enh1"
basic_enhanced_n_sequon="1" design_x_positions="1" pack_neighbors="1" scorefxn="sfx_clean" />
       <SimpleGlycosylateMover name="glycosylate_enh0" residue_selector="select_i_enh0"
glycosylation="a-D-Manp-(1->3)-[a-D-Manp-(1->3)-[a-D-Manp-(1->6)]-a-D-Manp-(1->6)]-[b-d-GlcpNAc-(1->4)]-b-D-M
anp-(1->4)-b-D-GlcpNAc-(1->4)-[a-L-Fucp-(1->6)]-b-D-GlcpNAc-" strip_existing="1" />
       <SimpleGlycosylateMover name="glycosylate_enh1" residue_selector="select_i_enh1"
glycosylation="a-D-Manp-(1->3)-[a-D-Manp-(1->3)-[a-D-Manp-(1->6)]-a-D-Manp-(1->6)]-[b-d-GlcpNAc-(1->4)]-b-D-M
anp-(1->4)-b-D-GlcpNAc-(1->4)-[a-L-Fucp-(1->6)]-b-D-GlcpNAc-" strip_existing="1" />
       <SymMinMover name="bb_min" scorefxn="sfx_clean" bb="1" chi="1" jump="0"
type="lbfgs_armijo_nonmonotone" tolerance="0.005" max_iter="100" />
       <GlycanTreeModeler name="tree_modeler" quench_mode="false" rounds="1" layer_size="1" window_size="0"
hybrid_protocol="1" shear="1" use_gaussian_sampling="1" glycan_sampler_rounds="150" />
       GlycanSampler name="tree_sampler" kt="0" rounds="1" pack_distance="5.0" scorefxn="sfx_clean" />
       GlycanRelaxMover name="basic_relax" />
       GlycanTreeRelax name="tree_relax" quench_mode="false" rounds="1" layer_size="2" window_size="1"/>
  </MOVERS>

  <PROTOCOLS>
       // wiggle backbone to loosen up a bit
       <Add mover_name="bb_min" />

       // generate sequon (enhanced or not) and add glycan
       <Add mover_name="create_motif_enh0" />
       <Add mover_name="create_motif_enh1" />

       // wiggle backbone to loosen up a bit
       <Add mover_name="bb_min" />

       // filter to extract energy of residues
       Add filter_name="total_energy_per_res_filter_i" />
       Add filter_name="total_energy_per_res_filter_i-2" />
       Add filter_name="fa_atr_per_res_filter" />
```

```
       Add filter_name="fa_rep_per_res_filter" />
       Add filter_name="fa_dun_per_res_filter" />
       Add filter_name="fa_elec_per_res_filter" />

       // add glycan and model glycan
       <Add mover_name="glycosylate_enh0" />
       <Add mover_name="glycosylate_enh1" />
       <Add mover_name="tree_modeler" />
       Add mover_name="tree_sampler" />
       Add mover_name="basic_relax" />
       Add mover_name="tree_relax" />
  </PROTOCOLS>
</ROSETTASCRIPTS>
```
